# Molecular Networking and Cultivation Profiling Reveals Diverse Natural Product Classes from an Australian Soil-Derived Fungus *Aspergillus* sp. CMB-MRF324

**DOI:** 10.3390/molecules27249066

**Published:** 2022-12-19

**Authors:** Taizong Wu, Angela A. Salim, Paul V. Bernhardt, Robert J. Capon

**Affiliations:** 1 Institute for Molecular Bioscience, The University of Queensland, Brisbane, QLD 4072, Australia; 2 School of Chemistry and Molecular Bioscience, The University of Queensland, Brisbane, QLD 4072, Australia

**Keywords:** *Aspergillus*, Australian soil-derived fungus, aspergillamide, asterriquinone, aflaquinolone, aspulvin, aspulvinone, GNPS molecular networking, MATRIX cultivation

## Abstract

This study showcases the application of an integrated workflow of molecular networking chemical profiling (GNPS), together with miniaturized microbioreactor cultivation profiling (MATRIX) to successfully detect, dereplicate, prioritize, optimize the production, isolate, characterize, and identify a diverse selection of new chemically labile natural products from the Queensland sheep pasture soil-derived fungus *Aspergillus* sp. CMB-MRF324. More specifically, we report the new tryptamine enamino tripeptide aspergillamides E–F (**7**–**8**), dihydroquinoline-2-one aflaquinolones H–I (**11**–**12**), and prenylated phenylbutyrolactone aspulvinone Y (**14**), along with an array of known co-metabolites, including asterriquinones SU5228 (**9**) and CT5 (**10**), terrecyclic acid A (**13**), and aspulvinones N-CR (**15**), B (**16**), D (**17**), and H (**18**). Structure elucidation was achieved by a combination of detailed spectroscopic and chemical analysis, biosynthetic considerations, and in the case of **11,** an X-ray crystallographic analysis.

## 1. Introduction

During our ongoing investigations into the chemistry of Australian microbes, we have reported numerous new structurally diverse natural products from many genera of fungi obtained from geographically dispersed and varying substrates. For example, our discoveries from the genus *Aspergillus* include a 2003 account of the *depsi*-cyclohexapeptide aspergillicins A–E from a Tasmanian estuarine sediment-derived *Aspergillus carneus* MST-MF156 [1]; a 2005 account of the heterocyclic dipeptide aspergillazines A–E from a remote outback Queensland soil-derived *A. unilateralis* MST-F8675 [2]; a 2009 account of the *nor*-methyl fumiquinazoline cottoquinazoline A and cyclopentapeptide cotteslosins A–B from a Western Australian beach sand-derived *A. versicolor* MST-MF495 [3]; a 2014 account of the P-glycoprotein inhibitory diketomorpholine shornephine A from a Queensland intertidal marine sediment-derived *Aspergillus* sp. CMB-M081F [4]; and a 2017 account of the nitro *depsi*-tetrapeptide diketopiperazine waspergillamide A from a Queensland mud dauber wasp-derived *Aspergillus* sp. CMB-W031 [5].

**Figure 1:**
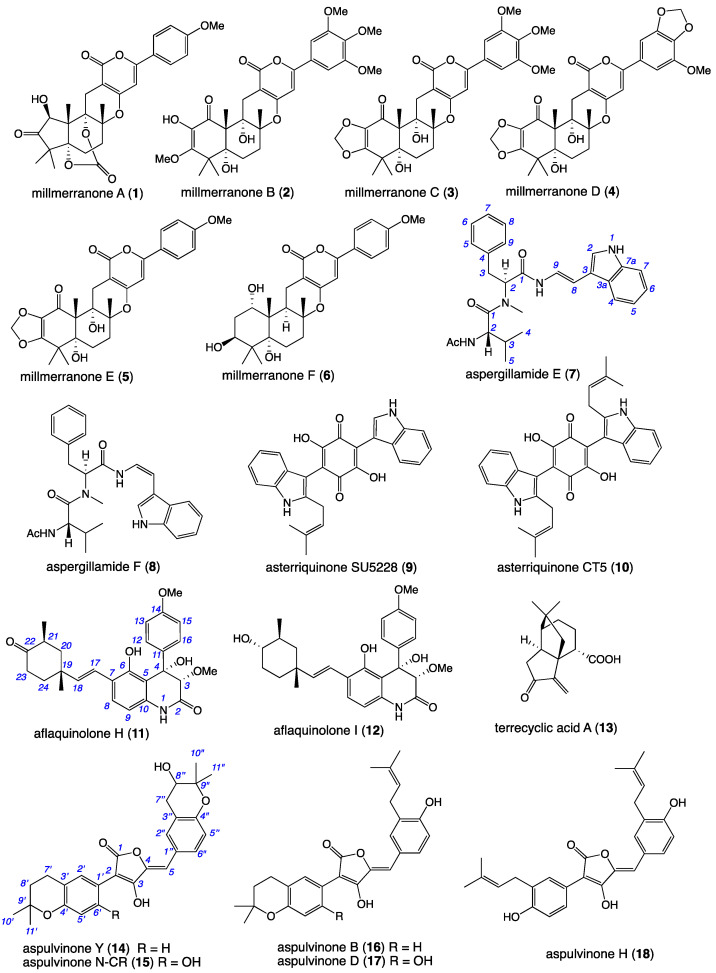
Natural products from *Aspergillus* sp. CMB-MRF324.

In recent years, these microbial biodiscovery efforts have been enhanced by the use of an integrated workflow consisting of global natural products social (GNPS) molecular networking [6] to detect, dereplicate, and prioritize, and a miniaturized microbioreactor methodology (MATRIX) [7] employing multiple different media under solid agar as well as static and shaken broth conditions to optimize the production of novel natural products. For example, in 2021 we reported a brown rice cultivation of a Queensland sheep pasture soil-derived *Aspergillus* sp. CMB-MRF324 yielding the acetylcholinesterase inhibitory meroterpene millmerranones A–F (**1**–**6**) (Figure 1), with millmerranone A being noteworthy as a rare example of a natural cyclic carbonate [8]. At that time, the GNPS analysis of a Sabouraud dextrose agar (SDA) cultivation of CMB-MRF324 revealed additional chemical diversity that was prioritized for future investigation.

This current report describes just such an investigation, where fractionation of the CMB-MRF324 SDA extract yielded new linear tripeptide aspergillamides E–F (**7**–**8**) as equilibrating *E/Z* amide rotamers incorporating a rare tryptamine enamine moiety, along with the known asterriquinones SU5228 (**9**) and CT5 (**10**) (Figure 1). In addition, we provide an account of a GNPS–MATRIX analysis of CMB-MRF324 that prompted discovery of the new dihydroquinoline-2-one aflaquinolones H (**11**) and I (**12**), the known terrecyclic acid A (**13**), and the new prenylated phenylbutyrolactone aspulvinone Y (**14**), and known aspulvinones N-CR (**15**), B (**16**), D (**17**) and H (**18**) (Figure 1). The detection, optimized production, isolation, and structure elucidation of **7**–**18** are outlined below.

## 2. Results and Discussion

**Figure 2:**
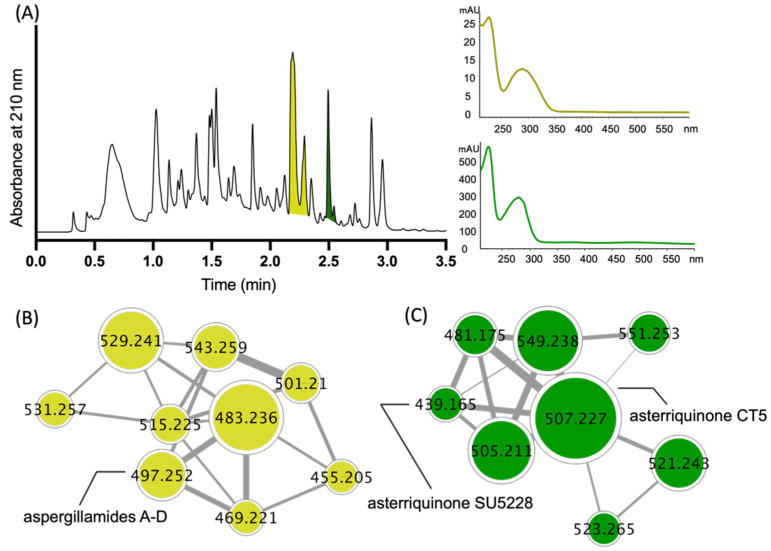
(**A**) UPLC-DAD (210 nm) chromatogram of the CMB-MRF324 SDA extract with UV-vis profiles for two key structure classes, and GNPS molecular families (clusters) for (**B**) aspergillamides and (**C**) asterriquinones. Highlights; aspergillamides (yellow); asterriquinones (green).

**Figure 3:**
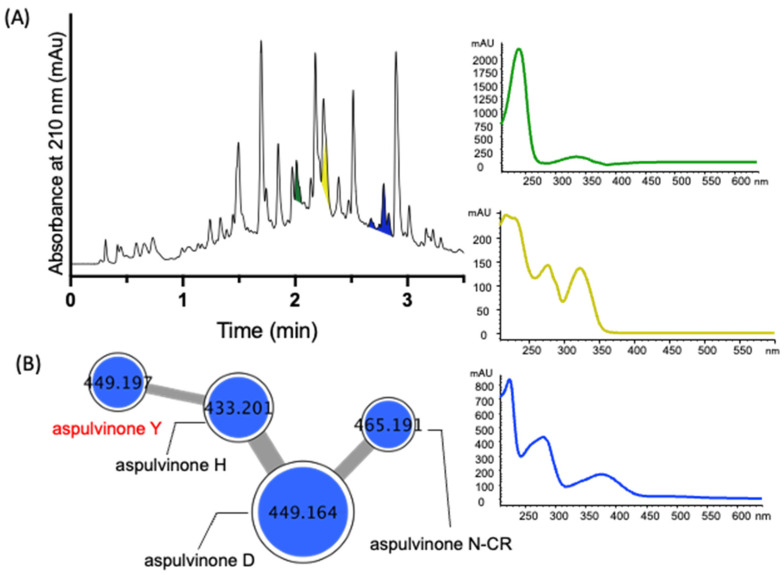
(**A**) UPLC-DAD (210 nm) chromatogram of an extract of a CMB-MRF324 brown rice cultivation with UV-vis profiles for three key structure classes and GNPS molecular families (clusters) for (**B**) aspulvinones. Highlights: aflaquinolones (yellow); terrecyclic acid A (green); aspulvinones (blue). Note—aflaquinolones and terrecyclic acid A do not form obvious GNPS molecular families.

A GNPS molecular network analysis of a selection of EtOAc extracts prepared from ×27 solid phase plate cultivations of pasture-derived fungi (SDA) and bacteria (ISP-2 agar) revealed two molecular families (clusters) unique to the fungus CMB-MRF324, both correlating with prominent peaks in the extract UPLC-DAD chromatogram (Figure 2A–C). Subsequent scaled-up SDA cultivation of CMB-MRF324 followed by extraction with EtOAc, solvent trituration and reversed phase chromatography (Figure S1) yielded the new tryptamine enamino tripeptide aspergillamides E–F (**7**–**8**) along with the known asterriquinones SU5228 (**9**) and CT5 (**10**) (Figure 1). Similarly, a GNPS based re-analysis of a brown rice cultivation of this same CMB-MRF324 fungus revealed a molecular family featuring potentially new chemistry, also evident in the UPLC-DAD chromatogram along with two other classes of metabolite (Figure 3A,B). Subsequent reversed phase and gel (LH-20) chromatography fractionation of a scaled up brown rice cultivation (Figure S2) yielded the new dihydroquinoline-2-one aflaquinolones H (**11**) and I (**12**) and prenylated phenylbutyrolactone aspulvinone Y (**14**), along with the known terrecyclic acid A (**13**) and aspulvinones N-CR (**15**), B (**16**), D (**17**), and H (**18**) (Figure 1). Structures for the known natural products **9** (Table S5 and Figures S19–S20) [9], **10** (Table S6 and Figures S21–S25) [10,11], **13** (Table S9 and Figures S40–S42) [12,13,14,15], **15** (Figures S48–S51) [16], **16** (Figures S52–S53) [17,18], **17** (Figures S54–S55) [17,18], and **18** (Figures S56–S57) [19] were established by spectroscopic analysis and comparison to data in the literature.

Of interest, although **10** possesses a plane-of-symmetry, this was not manifested in the 1D NMR data acquired in DMSO-*d*_6_ (Figure S21) or methanol-*d*_4_ (Figure S22), but was evident in the data acquired in CDCl_3_ (Figures S23 and S24). A possible explanation for this solvent effect (which has not been previously described) is that H-bonding in DMSO-*d*_6_ and methanol-*d*_4_ restricts/slows intramolecular rotations sufficiently to generate distinct and NMR detectable populations of atropisomeric stereoisomers, differentiating between a racemic mixture of P,P and M,M enantiomers on the one hand, and the *meso* P,M diastereomer on the other. As such, the apparent loss of symmetry in the NMR data is misleading and is perhaps better characterized as the detection of two separate isomer populations, each retaining a plane-of-symmetry. These phenomena suggest a need for caution when assessing the biological activity (and attempting any in silico modelling) of otherwise pure achiral (symmetric or asymmetric) compounds where H-bond mediated solvent effects (i.e., induced by aqueous assay media) can present as mixed populations of atropisomeric stereoisomers. An account of the structure elucidation of the new natural products **7**–**8**, **11**–**12,** and **14** is outlined below.

**Figure 4:**
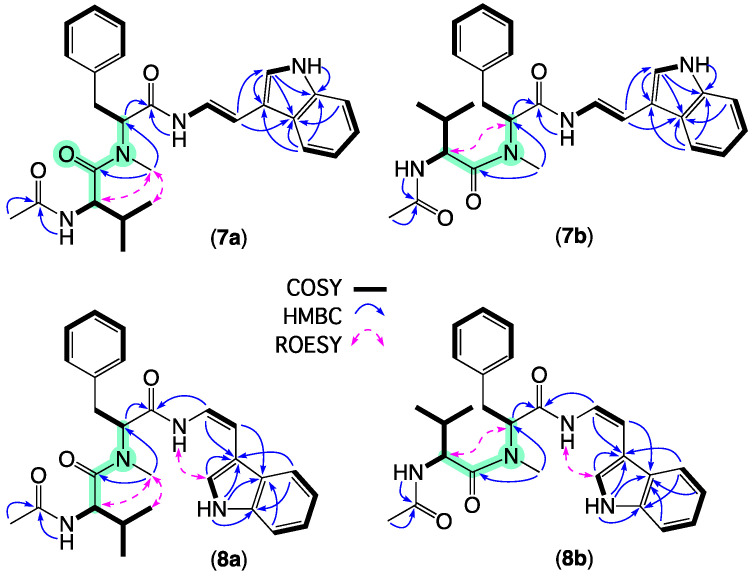
Selected 2D NMR (DMSO-*d*_6_) correlations for equilibrating *E* (**7a** and **8a**) and *Z* (**7b** and **8b**) amide rotamers of aspergillamides E–F (**7**–**8**).

HRESI(+)MS measurement on **7** returned a molecular formula (C_27_H_32_N_4_O_3_, Δmmu −1.9) requiring 14 double bond equivalents (DBE). The 1D NMR (DMSO-*d*_6_) data for **7** (Table 1 and Table 2, Tables S1 and S2 and Figure 4 and Figures S5–S10) revealed an isomeric mixture (ratio 1:0.6). Analysis of NMR resonances attributed to the major isomer **7a** revealed a mono substituted benzene, a C-2 mono substituted indole, an *E* 1,2-disubstituted double bond (*J_8,9_* 15 Hz), and three carbonyl carbons accounting for all DBE. Diagnostic 2D NMR correlations allowed assembly of the tripeptide scaffold for **7a**, with ROESY correlations between the terminal *N*-acetyl-valine and associated *N*-methyl amide moiety establishing **7a** as the *E* rotamer about the valine amide bond. Comparable analysis of the minor isomer **7b** confirmed the same planar structure with ROESY correlations between the phenylalanine and valine, a methines consistent with the *Z* rotamer about the valine amide bond. A Marfey’s analysis performed on **7** confirmed incorporation of l-valine and *N*-methyl-l-phenylalanine residues (Figure S4), permitting assignment of the structure for aspergillamide E (**7**) as shown.

HRESI(+)MS measurement on **8** returned a molecular formula (C_27_H_32_N_4_O_3_, Δmmu +2.5) isomeric with **7**. Comparison of the 1D and 2D NMR (DMSO-*d*_6_) data for **8** (Table 1 and Table 2, Tables S3 and S4 and Figure 4 and Figures S12–S17) with **7** revealed the same equilibrating major *E* (**8a**) and minor *Z* (**8b**) amide rotamers with key differences between **8** and **7** attributed to a *Z* enamino configuration (*J_8,9_* 9.5 Hz). A Marfey’s analysis performed on **8** confirmed the incorporation of l-valine and *N*-methyl-l-phenylalanine residues (Figure S4), permitting assignment of the structure for aspergillamide F (**8**) as shown. Alert to the potential for chemically reactive natural products to form handling artifacts [20], we determined that the *Z* geometric isomer **8** isomerizes on standing, under either acidic or neutral conditions, to the *E* isomer **7** and that this process is accelerated on heating. Nevertheless, chemical analysis of a fresh extract detected both **7** and **8**, confirming their status as natural products.

**Figure 5:**
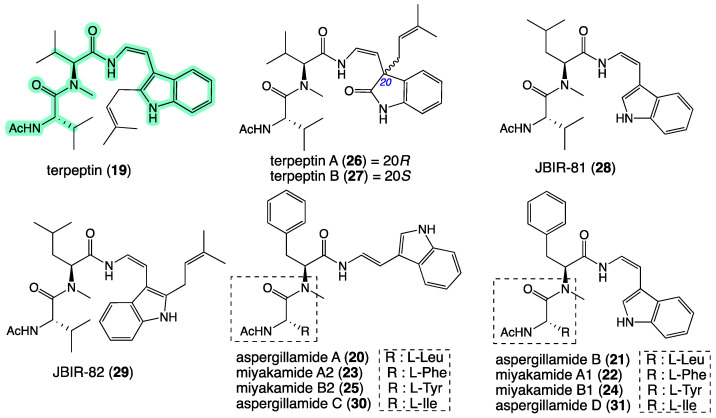
Known tryptamine enamino tripeptide natural products **19**–**31**. Highlighted: tryptamine enamino tripeptide scaffold (green).

**Figure 6:**
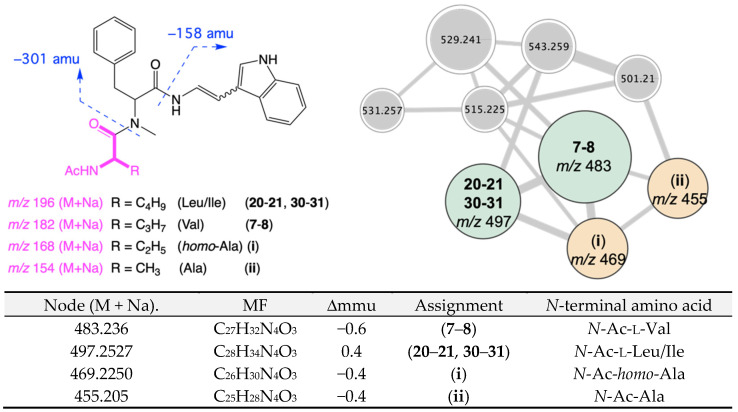
MS/MS fragmentation for nodes in the aspergillamide GNPS molecular family, revealing major aspergillamides E–F (**7**–**8**), possible minor known co-metabolite aspergillamides A–D (**20**–**21**, **30**–**31**) and putative new homologues, *homo*-Ala (**i**) and Ala (**ii**).

Tryptamine enamino tripeptides have appeared in the natural products literature on several occasions over the last 25 years, starting in 1997 with terpeptin (**19**) from a soil-derived *Aspergillus terreus* 95F-1 collected near Naha City, Okinawa Prefecture, Japan [21]; and progressing through 1998 with aspergillamides A–B (**20**–**21**) from a saline lake sediment-derived *Aspergillus* sp. CNC-120 collected in the Bahamas [22]; 2002 with miyakamides A_1_, A_2_, B_1,_ and B_2_ (**22**–**25**) from a fallen leaf-derived *Aspergillus flavus* Link var. *columnaris* FKI-0739 collected off Miyakojima Island, Japan [23]; 2008 with terpeptins A–B (**26**–**27**) from the mangrove plant (*Acanthus ilicifolius*) epiphyte *Aspergillus* sp. W–6 collected in Dongzhai Gang, China [24]; 2010 with JBIR-81 (**28**) and JBIR-82 (**29**) from the seaweed (*Sargassum* sp.) derived *Aspergillus* sp. SpD081030G1f1 collected off Ishigaki Island, Okinawa Prefecture, Japan [25]; and 2019 with aspergillamides C–D (**30**–**31**) from a marine sponge-derived *Aspergillus terreus* SCSIO 41008 collected off Guangdong Province, China [26] (Figure 5). It is noteworthy that all these reports were from fungi of the genus *Aspergillus*, albeit isolated from geographically diverse terrestrial and marine substrates. Encouraged by the relatively small number of reported members of this class of natural product, we returned our attention to the GNPS molecular network analysis and the MS/MS fragmentations that defined the aspergillamide molecular family. With diagnostic losses of 158 and 301 amu revealing the sodiated ion for the *N*-terminal amino acid, it was possible to detect nodes consistent with the known aspergillamides A–D (**20**–**21**, **30**–**31**), and for putative new very minor homologues, *homo*-Ala (**i**) and Ala (**ii**) (Figure 6 and Figures S58–S61).

**Figure 7:**
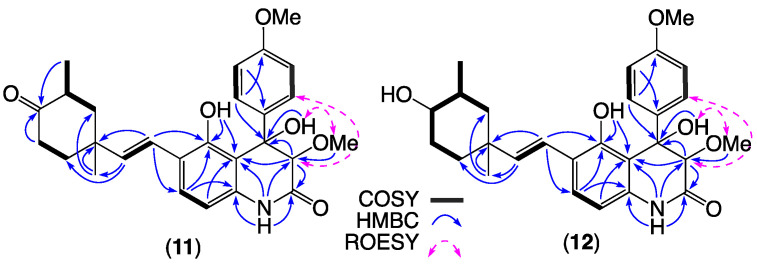
Selected 2D NMR (CDCl_3_) correlations for **11** and **12**.

**Figure 8:**
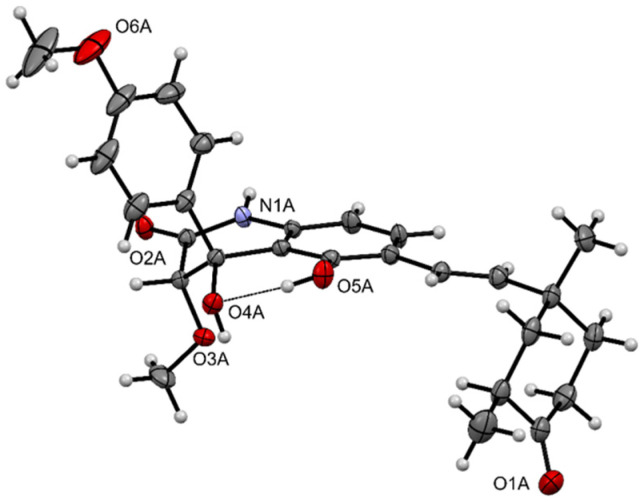
ORTEP view of the crystal structure of **11** (30% probability ellipsoids).

HRESI(+)MS measurement on **11** returned a molecular formula (C_27_H_31_NO_6_, Δmmu −2.0) requiring 13 DBE. The 1D NMR (CDCl_3_) data for **11** (Table 3 and Table S7, Figures S26–S31) revealed resonances for twelve *sp*^2^ carbons, of which eight could be attributed to two aromatic rings and four to an *E* 1,2-disubstituted double bond, an ester/amide, and a ketone carbonyl, accounting eleven DBE and requiring that **11** incorporate two additional rings. Diagnostic 2D NMR correlations permitted assembly of a planar structure (Figure 7) with a single crystal X-ray crystallographic analysis (Figure 8) unambiguously establishing the structure and absolute configuration of aflaquinolone H (**11**).

Remarkably, the crystal structure of **11** comprises six independent molecules in the asymmetric unit; molecule A is shown in Figure 8. All molecules adopt similar conformations with the only significant difference being the orientation of the 4-methoxy group on the phenyl ring which is found in one of two conformers related by a 180° rotation. An overlay of the six molecules is shown in the Supplementary Materials. A strong intramolecular H-bond (O5A-H…O4A 1.89 Å, 144.9°) corresponding to hydroxyl groups in the 4- and 6-positions of the 3, 4-dihydroquinolinone ring system is apparent in Figure 8, and this is present in all six molecules comprising the structure. This H-bond restrains the 4-hydroxy group (O4A) in an equatorial position, and this locks the confirmation of the bicyclic ring system leaving the *p*-methoxyphenyl substituent in an axial position. The absolute configuration of **11** was established by the Bijvoet analysis of Hooft and co-workers; *P*2 = 1.000, Hooft parameter *y* = −0.06(9) [27].

HRESI(+)MS measurement on **12** revealed a molecular formula (C_27_H_33_NO_6_, Δmmu −1.0), consistent with a hydrogenated analogue of **11**. Comparison of the NMR (CDCl_3_) data for **12** (Table 3 and Table S8, Figures S33–S38) with **11** showed a high level of similarity, with differences attributed to replacement of the C-22 ketone in **11** (*δ*_H_ 214.0) with a C-22/H-22 2°-OH in **12** (*δ*_H_ 3.12, ddd, *J* = 10.5, 10.5, 4.5 Hz, H-22; *δ*_C_ 77.3, C-22), further confirmed by diagnostic 2D NMR correlations (Figure 7). The β-facial axial orientation of H-22 was evident from large values for *J*_21,22_ and *J*_22,23a_ (10.5 and 10.5 Hz) which together with biogenetic considerations permitted assignment of the full structure for aflaquinolone I (**12**).

**Figure 9:**
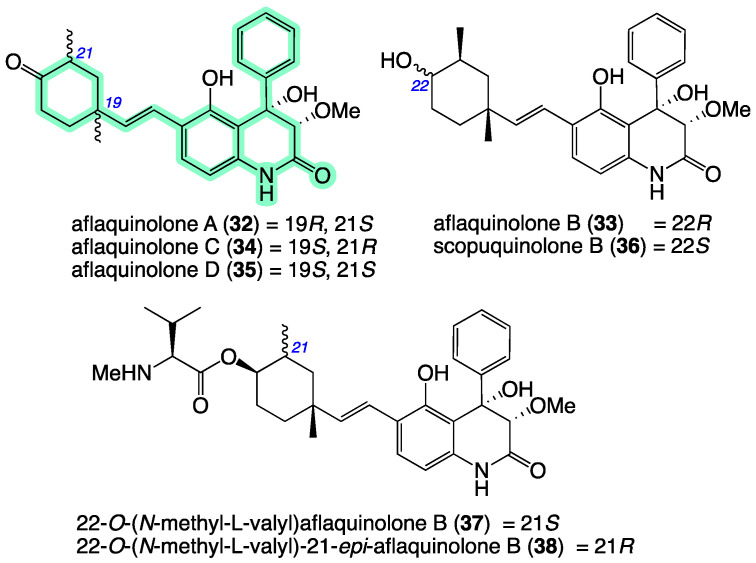
Structures for known aflaquinolones and analogues **32**–**38**. Highlighted: carbo/heterocyclic scaffold (green).

Aflaquinolones H–I (**11**–**12**) from *Aspergillus* sp. CMB-MRF324 bear a carbo/heterocyclic scaffold in common with a small number of known fungal metabolites: aflaquinolones A–D (**32**–**35**) reported in 2012 from the Hawaiian *Aspergillus* sp. NRRL 58570 and Korean *Aspergillus* sp. SF-5044 [28]; scopuquinolone B (**36**) reported in 2018 from a coral-derived *Scopulariopsis* sp. [29]; and 22-*O*-(*N*-methyl-L-valyl)aflaquinolone B (**37**) and 22-*O*-(*N*-methyl-L-valyl)-21-*epi*-aflaquinolone B (**37**) reported in 2014 from the South China Sea gorgonian-derived *Aspergillus* sp. XS-20090B15, as nM potent inhibitors of the respiratory syncytial virus (RSV) [30] (Figure 9).

**Figure 10:**
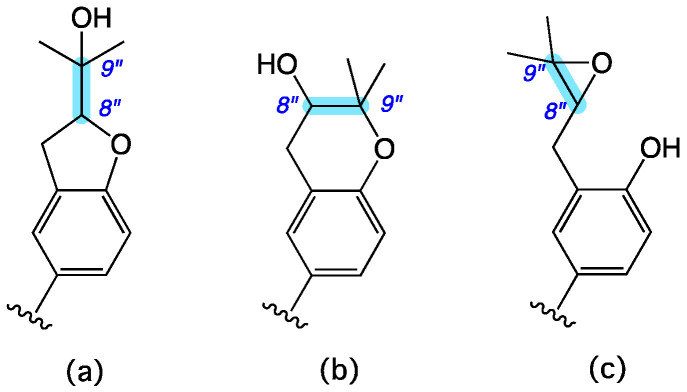
Isomeric substructures featuring a substituted (**a**) dihydrobenzofuran, (**b**) dihydrobenzopyran, and (**c**) epoxide. Highlight (blue) indicated key oxygenated residues.

**Figure 11:**
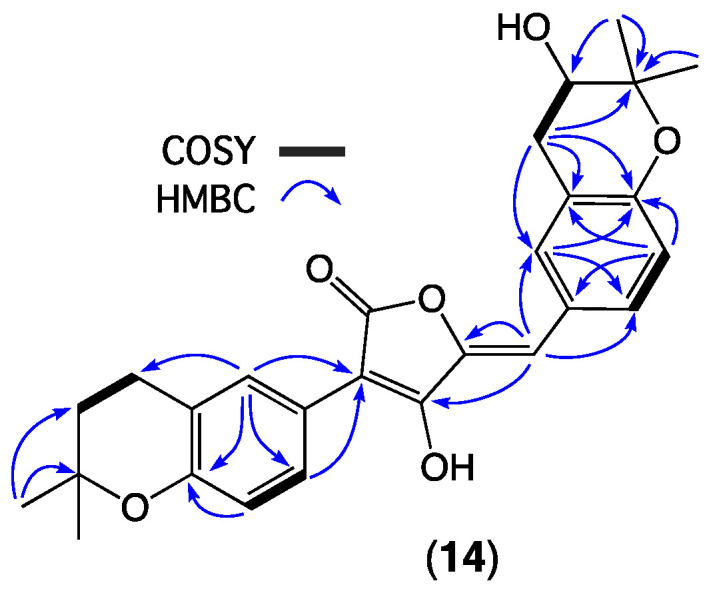
Selected 2D NMR (methanol-*d*_4_) correlations for **14**.

HRESI(+)MS measurement on **14** returned a molecular formula (C_27_H_28_O_6_, Δmmu −2.4) suggestive of an oxidized (+O) analogue of the co-metabolite aspulvinone B (**16**). Comparison of the 1D NMR (methanol-*d*_4_) data for **14** (Table 4 and Table S10, Figures S43–S46) with the 1D NMR (acetone-*d*_6_) data for **16** (Figure S52, Note—**16** proved insoluble in methanol) revealed a high level of similarity, with key differences attributed to replacement of the isoprene D^8″,9″^ moiety in **16** (d_H_ 5.36, m, H-8″; *δ*_C_ 123.3, C-8″; *δ*_C_ 133.1, C-9″) with a dioxygenated moiety in **14** (*δ*_H_ 3.82, dd, H-8″; *δ*_C_ 69.4, C-8″; *δ*_C_ 78.3, C-9″). The latter resonances could in principle be attributed to three possible substructures (Figure 10) consisting of a dihydrobenzofuran, a dihydrobenzopyran, or an epoxide. The dihydrobenzofuran moiety (Figure 10a) is prominent among known aspulvinones (Figure S62) and features a consistent range of ^13^C NMR (methanol-*d*_4_) chemical shifts for C-8″ (*δ*_C_ 89.9–91.4) and C-9″ (*δ*_C_ 71.1–73.1) [16,31]. While the dihydrobenzopyran moiety (Figure 10b) is less common among known aspulvinones (Figure S62), there are nevertheless reports of the ^13^C NMR (methanol-*d*_4_) chemical shifts for C-8″ (*δ*_C_ 68.7–71.0) and C-9″ (*δ*_C_ 77.2–77.5) [16]. Finally, although there are no known accounts of aspulvinones featuring an epoxy moiety (Figure 10c), there are reports of ^13^C NMR chemical shifts that are relevant to both C-8″ (*δ*_C_ 60–65) and C-9″ (*δ*_C_ 57–59) [32]. These spectroscopic comparisons, together with diagnostic 2D NMR correlations (Figure 11), and biogenetic considerations, permitted assignment of the structure for aspulvinone Y (**14**) as shown. The sample of **14** proved unstable and decomposed during NMR data acquisition, precluding measurement of an optical rotation and consideration of absolute configuration.

A review of the scientific literature revealed a rich history of fungal natural products belonging to the aspulvinone structure class. While these originated with 1973 [17] and 1975 [18] accounts of the pulvinones from *Aspergillus terreus* IAM 2054, the trivial nomenclature and structures were subsequently revised in 1976 [19] and 1979 [33] as aspulvinones A–I. These early discoveries were followed by reports of new members of this structure class, including a 2011 account of aspulvinones I-CR to N-CR from a Costa Rican marine sediment-derived *Aspergillus* sp. 05545 [16]; a 2013 account of isoaspulvinone E from a Fijian marine mangrove rhizosphere soil-derived *Aspergillus terreus* Gwq-48 [34]; a 2013 account of aspulvinone B1 from *Aspergillus terreus* NIH 2624 [35]; a 2015 account of aspulvinone O from a Chinese marine red alga ephiphyte *Paecilomyces variotii* EN-291 [36] (curiously the trivial nomenclature “aspulvinones J–N” do not appear to have been utilised); a 2016 account of aspulvinone P–Q from a Chinese wetland mud-derived *Aspergillus flavipes* PJ03-11 [37]; a 2017 account of aspulvinone R from a Chinese medicinal plant-derived *Aspergillus* sp. CPCC 400735 [38]; a 2021 account of aspulvinones R–U from a Thai marine sponge-derived *Aspergillus flavipes* KUFA1152 [39] (note—the trivial nomenclature “aspulvinone R” had been prior assigned to a different fungal metabolite in 2017 [38]); a 2021 account of aspulvinones V-X from a marine-derived *Aspergillus terreus* ASM-1 [31]; a 2022 account of aspulvins A–H from the Chinese medicinal plant-derived *Cladosporium* sp. 7951 [40]; and a 2022 account of aspulvinones S–V from the Taiwanese marine green alga-derived *Aspergillus terreus* NTU243 [41] (note—the trivial nomenclature “aspulvinones S-V” had been prior assigned to different fungal metabolites in 2021 [31,39]). Given this history, we attributed the trivial nomenclature aspulvinone Y to **14**.

**Figure 12:**
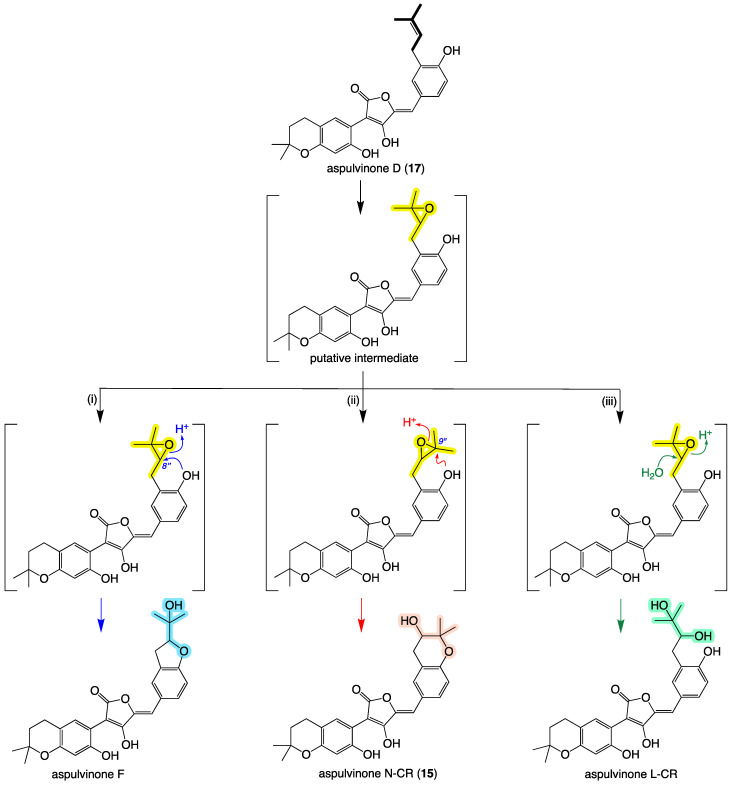
A plausible biogenetic relationship linking prenylated to hydroxylated and heterocyclic aspulvinones (i–iii three different pathways to yield various aspulvinone analogs). Highlighted (bold, yellow, blue, red, green) indicate biogenetically related residues.

Aspulvinone D (**17**) may be considered as the biosynthetic precursor of other aspulvinone analogs via a putative epoxide intermediate, which can be further modified (either enzymatically or non-enzymatically) [20] by one of three pathways: (i) intramolecular S_N_2 addition of the C-4″ phenol moiety to C-8″ with concomitant opening of the epoxide to yield aspulvinone F, (ii) intramolecular S_N_2 addition of the C-4″ phenol moiety to C-9″ with concomitant opening of the epoxide to yield aspulvinone N–CR (**15**), or (iii) S_N_2 addition of H_2_O to C-9″ (or C-8″) with concomitant opening of the epoxide to yield aspulvinone L–CR (Figure 12). This same set of transformations could in principle be applied to other (as yet undetected) aspulvinone/aspulvin epoxides as a pathway to all known heterocyclic members of this structure class.

In summary, an integrated workflow of chemical (GNPS) and cultivation (MATRIX) profiling has proved effective at exploring the chemical diversity encoded within the genome of the Queensland sheep pasture soil-derived fungus *Aspergillus* sp. CMB-MRF324. In addition to meroterpene millmeranones A–F (**1**–**6**), this latest study has demonstrated that CMB-MRF324 also produces new tryptamine enamino tripeptides, aspergillamide E–F (**7**–**8**), new dihydroquinoline-2-one aflaquinolones H–I (**11**–**12**), and a new prenylated phenylbutyrolactone aspulvinone Y (**14**), along with an array of known metabolites. The majority of the new natural products documented in this study proved chemically unstable once purified, further demonstrating the importance of employing effective and sensitive analytical methods to monitor and ensure chemical integrity during purification, characterisation, handling, and storage.

## 3. Materials and Methods

### 3.1. General Experimental Procedure

Chiroptical measurements ([α]_D_) were obtained on a JASCO P-1010 polarimeter (JASCO International Co. Ltd., Tokyo, Japan) in a 100 × 2 mm cell at specified temperatures. Nuclear magnetic resonance (NMR) spectra were acquired on a Bruker Avance 600 MHz spectrometer (Bruker Pty. Ltd., Preston, VIC, Australia) with either a 5 mm PASEL ^1^H/D-^13^C Z-Gradient probe or 5 mm CPTCI ^1^H/^19^F-^13^C/^15^N/DZ-Gradient cryoprobe. In all cases, spectra were acquired at 25 °C in DMSO-*d*_6_ or CDCl_3_ or methanol-*d*_4_ or acetone-*d*_6_ with referencing to residual ^1^H or ^13^C signals (DMSO-*d*_6_, δ_H_ 2.50 and δ_C_ 39.5; CDCl_3_ δ_H_ 7.26 and δ_C_ 77.1; methanol-*d*_4_, δ_H_ 3.31 and δ_C_ 49.0; acetone-*d*_6_, δ_H_ 2.05). High-resolution ESIMS spectra were obtained on a Bruker micrOTOF mass spectrometer (Bruker Pty. Ltd., Preston, VIC, Australia) by direct injection in MeOH at 3 μL/min using sodium formate clusters as an internal calibrant. Liquid chromatography diode array mass spectrometry (HPLC-DAD-MS) data were acquired either on an Agilent 1260 infinity II separation module equipped with an Agilent G6125B LC/MSD mass detector (Agilent Technologies Pty. Ltd, Mulgrave, VIC, Australia) and diode array detector or on Shimadzu LCMS-2020 LCMS (Shimadzu Pty. Ltd., Rydalmere, NSW, Australia). Semi-preparative HPLC were performed using Agilent 1100 series HPLC instruments with corresponding detectors, fraction collectors and software. UPLC chromatograms were obtained on Agilent 1290 infinity UPLC system equipped with diode array multiple wavelength detector (Agilent Zorbax C_8_ RRHD 1.8 μm, 50 × 2.1 mm column (Agilent Technologies Pty. Ltd., Mulgrave, VIC, Australia), gradient elution over 2.50 min at 0.417 mL/min from 90% H_2_O/MeCN to 100% MeCN with an isocratic 0.01% TFA modifier). UPLC-QTOF analysis was performed on an Agilent 1290 Infinity II UPLC (Agilent Zorbax C_8_ RRHD 1.8 μm, 50 × 2.1 mm column, gradient elution over 2.50 min at 0.417 mL/min from 90% H_2_O/MeCN to 100% MeCN with an isocratic 0.1% formic acid/MeCN modifier) coupled to an Agilent 6545 Q-TOF (Agilent Technologies Pty. Ltd, Mulgrave, VIC, Australia). MS/MS analysis was performed on the same instrument for ions detected in the full scan at an intensity above 1000 counts at 10 scans/s, with an isolation width of 4 *m/z* using a fixed collision energy and a maximum of 3 selected precursors per cycle. Chemicals were purchased from Sigma-Aldrich or Merck unless otherwise specified. Analytical grade solvents were used for solvent extractions. Chromatography solvents were of HPLC grade supplied by Labscan or Sigma-Aldrich and filtered/degassed through 0.45 μm polytetrafluoroethylene (PTFE) membrane prior to use. Deuterated solvents were purchased from Cambridge Isotopes. Microorganisms were manipulated under sterile conditions using a Laftech class II biological safety cabinet and incubated in either MMM Friocell incubators (Lomb Scientific Pty. Ltd., Taren Point, NSW, Australia) or an Innova 42R incubator shaker (John Morris Scientific Pty. Ltd., Chatswood, NSW, Australia).

### 3.2. Collection and Taxonomy of CMB-MRF324

The fungus CMB-MRF324 was isolated from the soil sample collected from a sheep pasture near Millmerran, Queensland, Australia. Following 7 days cultivation on an SD agar plate at 30 °C., Genomic DNA was extracted from the mycelia using the DNeasy Plant Mini Kit (Qiagen, Clayton, VIC, Australia), the 18S rRNA genes were amplified, and BLAST analysis (NCBI database) confirmed the closest homology with *Aspergillus terreus* (Genbank accession number: MZ823609) as previously reported [8].

### 3.3. Scale-Up SDA Cultivation and Fractionation of CMB-MRF324

CMB-MRF324 was incubated at 30 °C for 14 days on ×20 SDA plates after which the combined agar and mycelia was harvested and extracted with EtOAc (2 × 300 mL) and the organic phase concentrated *in vacuo* to yield an extract (732 mg). The extract was then subjected to sequential trituration to afford (after drying under nitrogen at 40 °C) n-hexane (380 mg) and MeOH (324 mg) solubles, with the latter subjected to preparative reverse-phase HPLC (Phenomenex Luna-C_8_ 10 μm, 21.2 × 250 mm column (Phenomenex Inc., Lane Cove, NSW, Australia), 20 mL/min gradient elution over 25 min from 60% H_2_O/MeCN to 15% H_2_O/MeCN with an isocratic 0.01% TFA/MeCN modifier) to yield fractions F1–F2. Fraction F1 was then subjected to semi-preparative HPLC (Agilent Zorbax C_8_ 5 μm, 250 × 9.4 mm column (Agilent Technologies Pty. Ltd., Mulgrave, VIC, Australia), 3 mL/min isocratic elution over 20 min at 55% H_2_O/MeCN to yield **7** (7.0 mg, 0.9%) and **8** (4.0 mg, 0.5%), while Fraction F2 was subjected to semi-preparative HPLC (Agilent Zorbax Eclipse XDB C_8_ 5 μm, 250 × 9.4 mm column (Agilent Technologies Pty. Ltd., Mulgrave, VIC, Australia), 3 mL/min gradient elution over 15 min from 40% H_2_O/MeCN to 25% H_2_O/MeCN, with an isocratic 0.01% TFA/MeCN modifier) to yield **9** (5.0 mg, 0.7%) and **10** (5.0 mg, 0.7%). (Note: All % yields are weight to weight estimates based on unfractionated EtOAc extract).

### 3.4. Scale-Up Brown Rice Cultivation and Fractionation of CMB-MRF324

CMB-MRF324 was incubated at room temperature for 24 days in rice-based medium (70 g brown rice, 0.3 g peptone, 0.3 g yeast extract, 0.1 g monosodium glutamate, in 100 mL distilled H_2_O) after which the medium and mycelia were extracted with EtOAc (3 × 500 mL) and the organic phase concentrated in vacuo to yield an extract (3.8 g). The extract was then subjected to sequential trituration to afford (after drying in vacuo) n-hexane, CH_2_Cl_2_ (3.05 g), and MeOH solubles. A portion of the CH_2_Cl_2_ solubles (2.4 g) was subjected to preparative HPLC (Phenomenex Luna-C_8_ 10 μm, 21.2 × 250 mm column, 20 mL/min gradient elution over 30 min from 90% H_2_O/MeCN to 100% MeCN with an isocratic 0.01% trifluoroacetic acid/MeCN modifier) to yield 40 fractions (F1–F40). F18 (50 mg) was subjected to semi-preparative HPLC (Agilent Zorbax SB C_3,_5 μm, 9.4 × 250 mm column, 3 mL/min gradient elution over 20 min from 60% MeCN/H_2_O to 55% MeCN/H_2_O with an isocratic 0.01% TFA/MeCN modifier) to yield **13** (4.0 mg, 0.1%). Combined F21–F24 (230 mg) were subjected to gel chromatography (Sephadex LH-20 (Sigma-Aldrich Pty. Ltd, Macquarie Park, NSW, Australia) gravity elution with 50% MeOH/CH_2_Cl_2_) followed by semi-preparative HPLC (Agilent Zorbax SB C_3_ 5 μm, 9.4 × 250 mm column, 3 mL/min isocratic elution over 25 min with 55% H_2_O/MeCN with an isocratic 0.01% TFA/MeCN modifier) to yield **11** (5.0 mg, 0.2%) and **12** (1.6 mg, 0.06%). F25 was subjected to semi-preparative HPLC (Agilent Zorbax Eclipse XDB C_8_ 5 μm, 9.4 × 250 mm column, 3 mL/min isocratic elution over 20 min with 45% H_2_O/MeCN with an isocratic 0.01% TFA/MeCN modifier) to yield **14** (1.0 mg, 0.03%), **15** (1.0 mg, 0.03%), and **16** (3.0 mg, 0.09%). F31 subjected to semi-preparative HPLC (Agilent Zorbax Eclipse XDB C_8_ 5 μm, 9.4 × 250 mm column, 3 mL/min gradient elution over 20 min from 40% H_2_O/MeCN to 35% H_2_O/MeCN with an isocratic 0.01% TFA/MeCN modifier) to yield **17** (2.5 mg, 0.07%) and **18** (2.5 mg, 0.07%). (Note: All % yields are weight to weight estimates based on unfractionated EtOAc extract).

### 3.5. Metabolite Characterization

Aspergillamide E (**7**). White powder; NMR (600 MHz, DMSO-*d*_6_), Table 1, Table 2, Tables S1 and S2, Figures S5–S10; HRESI(+)MS *m/z* 483.2348 [M + Na]^+^ (calcd for C_27_H_32_N_4_NaO_3_ 483.2367).

Aspergillamide F (**8**). white powder; 1D and 2D NMR (DMSO-*d*_6_), Table 1, Table 2, Tables S3 and S4, Figures S12–S17; HRESI(+)MS *m/z* 483.2392 [M + Na]^+^ (calcd for C_27_H_32_N_4_NaO_3_ 483.2367).

Asterriquinone SU5228 (**9**). 1D NMR (DMSO-*d*_6_), Table S5, Figure S19; HRESI(+)MS *m/z* 461.1475 [M + Na]^+^ (calcd for C_27_H_22_NaN_2_O_4_ 461.1472).

Asterriquinone CT5 (**10**). 1D NMR (DMSO-*d*_6_), Table S6, Figures S21–S24; HRESI(+)MS *m/z* 507.2282 [M + H]^+^ (calcd for C_32_H_31_N_2_O_4_ 507.2278).

Aflaquinolone H (**11**). colorless crystal; [α]_D_ +55 (c 0.1, MeOH); 1D and 2D NMR (CDCl_3_), Table 3 and Table S7, Figures S26–S31; HRESI(+)MS *m/z* 488.2044 [M + H]^+^ (calcd for C_27_H_31_NO_6_ 488.2064).

Aflaquinolone I (**12**). White powder; [α]_D_ +16 (c 0.1, MeOH); 1D and 2D NMR (CDCl_3_), Table 3 and Table S8, Figures S33–S38; HRESI(+)MS *m/z* 490.2200 [M + H]^+^ (calcd for C_27_H_33_NO_6_ 490.2210).

Terrecyclic acid A (**13**) [14]. [α]_D_ -57 (c 0.1, CHCl_3_); 1D NMR (DMSO-*d*_6_), Table S9, Figures S40–S41; HRESI(+)MS *m/z* 249.1506 [M + H]^+^ (calcd for C_15_H_21_O_3_ 249.1485).

Aspulvinone Y (**14**). Yellowish powder; 1D and 2D NMR (methanol-*d*_4_), Table 4 and Table S10, Figures S43–S46; HRESI(+)MS *m/z* 449.1959 [M + H]^+^ (calcd for C_27_H_29_O_6_ 449.1983).

Aspulvinone N-CR (**15**). Yellowish powder; 1D and 2D NMR (acetone-*d*_6_), Table 4 and Table S11, Figures S48–S50; HRESI(+)MS *m/z* 465.1908 [M + H]^+^ (calcd for C_27_H_29_O_7_ 465.1917).

Aspulvinone B (**16**) [42]. A 1D NMR (acetone-*d*_6_), Figure S52; HRESI(+)MS *m/z* 455.1828 [M + Na]^+^ (calcd for C_27_H_28_NaO_5_ 455.1829).

Aspulvinone D (**17**) [42]. A 1D NMR (acetone-*d*_6_), Figure S54; HRESI(+)MS *m/z* 449.1969 [M + H]^+^ (calcd for C_27_H_29_O_6_ 449.1959).

Aspulvinone H (**18**) [35,43]. A 1D NMR (methanol-*d*_4_), Figure S56; HRESI(+)MS *m/z* 433.2029 [M + H]^+^ (calcd for C_27_H_29_O_5_ 433.2010).

### 3.6. Marfey’s Analysis of Aspergillamides E (**7**) and F (**8**)

A sample of analyte (i.e., **7** or **8**) (100 μg) in 6M HCl (200 μL) was heated overnight at 110 °C after which the sample was concentrated to dryness under N_2_ and the residue treated with 1N NaHCO_3_ (20 μL), 1% (*w*/*v*) 1-fluoro-2,4-dinitro-5-l-alanine amide (l-FDAA) in acetone (50 μL) at 40 °C for 60 min, followed by the addition of 1N HCl (20 μL) and dilution with MeCN prior to HPLC-MS analysis. Samples of the authentic amino acid standards l-valine and *N*-methyl-l-phenylalanine were subjected to the same derivatisation process with both l-FDAA and d-FDAA. Aliquots (5 μL) of the FDAA derivatised analytes and standards were subjected to HPLC-DAD-MS (Agilent Poroshell EC-C_8_ 2.7 μm, 3.0 × 150 mm, 0.8 mL/min isocratic elution over 12 min with 70% H_2_O/MeCN with an isocratic 0.05% formic acid modifier) to confirm that **7** and **8** incorporated l-valine and N-methyl-l-phenylalanine (Figure S4).

### 3.7. Isomerisation of Aspergillamide F (**8**) to E (**7**)

Three samples A-C of **8** (10 μg in 100 μL MeOH each) were prepared. Sample A was stirred at room temperature for 24 h. Sample B was treated with 0.1% TFA at room temperature for 24 h. Sample C was heated at 60 °C for 24 h. After 24 h aliquots (5 μL) of each were analysed by UPLC-DAD (Zorbax C_8_ RRHD 1.8 μm, 50 × 2.1 mm column, gradient elution over 2.50 min at 0.417 mL/min from 90% H_2_O/MeCN to 100% MeCN with an isocratic 0.01% TFA modifier) to reveal that **8** transformed to **7** slowly at room temperature even in the presence of acid, while heating at 60 °C accelerated this conversion (Figure S3).

### 3.8. X-ray Crystallography

Crystals of **11** were obtained by slow evaporation from 50% MeOH/CH_2_Cl_2_ at room temperature. Crystallographic data (CuKα radiation 1.54184 Å, 2θmax = 125°) were collected on an Oxford Diffraction Gemini S Ultra CCD diffractometer with the crystal cooled to 190 K with an Oxford Cryosystems Desktop Cooler. Data reduction and empirical absorption corrections were carried out with the CrysAlisPro program (Oxford Diffraction vers. 171.38.46). The structure was solved by direct methods with SHELXT and refined with SHELXL [44]. The thermal ellipsoid diagrams were generated with Mercury [45]. All crystallographic calculations were carried out within the WinGX graphical user interface [46]. The crystal structures data in CIF format have been deposited in the Cambridge Structural Database (CCDC 2217840) (see Figure 8 and Supplementary Materials).

## Figures and Tables

**Table 1:** ^1^H NMR (DMSO-*d*_6_) Data for Aspergillamides E–F (**7**–**8**).

Position	7a *δ*_H_, Mult (*J* in Hz)	7b *δ*_H_, Mult (*J* in Hz)	8a *δ*_H_, Mult (*J* in Hz)	8b *δ*_H_, Mult (*J* in Hz)
enamino-Trp				
2	7.42, d (2.2)	7.45, d (2.2)	7.53, d (2.3)	7.71, d (2.3)
4	7.61, d (7.7)	7.62, d (7.7)	7.57, d (7.9)	7.57 ^B^
5	7.07, dd (7.7, 7.7)	7.11, m	7.02, dd (7.9, 7.3)	7.03 ^B^
6	7.12, m	7.07, m	7.12, dd (7.9, 7.3)	7.12 ^B^
7	7.38, d (8.0)	7.36, d (8.0)	7.38, d (7.9)	7.37, d (8.3)
8	6.42, d (15.0)	6.52, d (15.0)	5.95, d (9.5)	5.99, d (9.5)
9	7.27, dd (15.0, 9.9)	7.32, dd (15.0, 9.9)	6.64, dd (10.2, 9.5)	6.61, dd (9.8, 9.5)
1-NH	11.1, d (2.2)	11.1, d (2.2)	11.29, br s	11.30, br s
9-NH	9.93, d (9.9)	9.92, d (9.9)	9.01, d (10.2)	9.23, d (9.8)
*N*-Me-l-Phe				
2	5.27, dd (9.6, 6.1)	4.98, dd (9.6, 5.2)	5.53, dd (9.9, 6.0)	5.30, dd (8.8, 6.0)
3	*a.* 3.23, dd (14.3, 6.1)	*a.* 3.32 ^A^	*a.* 3.23, dd (15.0, 6.0)	*a.* 3.37 ^A^
	*b.* 2.97, dd (14.3, 9.6)	*b.* 2.95, d (14.3, 6.1)	*b.* 3.00, dd (15.0, 9.9)	*b.* 2.96, dd (14.4, 8.8)
5	7.21, d (7.1)	7.29, d (7.1)	7.23, d (7.1)	7.30, d (7.0)
6	7.24, dd (7.1, 7.1)	7.30, dd (7.1, 7. 1)	7.22, dd (7.1, 7.1)	7.29, dd (7.0, 7.0)
7	7.18, t (7.1)	7.22, t (7.1)	7.17, t (7.1)	7.22 ^B^
N-Me	3.08, s	2.81, s	3.03, s	2.89, s
l-Val				
2	4.44, dd (8.7, 8.7)	4.29, dd (7.6, 7.6)	4.46, dd (8.9, 8.9)	4.26, dd (8.1, 8.1)
3	1.90, dqq (8.7, 6.9, 6.9)	1.23, dqq (7.6, 6.9, 6.9)	1.89, dqq (8.9, 6.7, 6.7)	1.56, dqq (8.1, 6.8, 6.8)
4^C^	0.81, d (6.9)	0.63, d (6.9)	0.72, d (6.7)	0.48, d (6.8)
5^C^	0.84, d (6.9)	0.48, d (6.9)	0.77, d (6.7)	0.69, d (6.8)
NH	7.97, d (8.7)	8.26, d (7.6)	7.9, d (8.9)	8.24, d (8.1)
NHAc	1.79, s	1.91, s	1.73, s	1.66, s

^A^ Resonance obscured by residual H_2_O and detected by HSQC; ^B^ *E/Z* amide rotamer resonances overlapping.

**Table 2:** ^13^C NMR (DMSO-*d*_6_) Data for aspergillamides E–F (**7–8**).

Position	7a *δ*_C_, Type	7b *δ*_C_, Type	8a *δ*_C_, Type	8b *δ*_C_, Type
enamino-Trp				
2	123.5, CH	123.6, CH	123.6, CH	124.4, CH
3	111.5, C	111.4, C	109.5, C	109.2, C
3a	124.8, C	124.9, C	126.5, C	126.6, C
4	118.9, CH	118.8, CH	118.3, CH	118.3, CH
5	119.3, CH	119.3, CH	119.1, CH	119.1, CH
6	121.4, CH	121.4, CH	121.6, CH	121.5, CH
7	111.8, CH	111.8, CH	111.5, CH	111.4, CH
7a	136.8, C	136.8, C	135.6, C	135.6, C
8	106.8, CH	107.0, CH	103.3, CH	104.2, CH
9	119.5, CH	119.5, CH	118.1, CH	117.9, CH
*N*-Me-l-Phe				
1	167.0, C	166.1, C	168.4, C	167.6, C
2	57.7, CH	61.3, CH	56.8, CH	60.6, CH
3	34.1, CH_2_	34.2, CH_2_	33.5, CH_2_	35.4, CH_2_
4	137.6, C	137.8, C	137.4, C	137.5, C
5	128.8, CH	129.3, CH	128.8, CH	129.3, CH
6	128.1, CH	128.4, CH	128.1, CH	128.3, CH
7	126.3, CH	126.5, CH	126.3, CH	126.5, CH
*N*-Me	32.3, CH_3_	29.3, CH_3_	31.6, CH_3_	29.7, CH_3_
l-Val				
1	172.4, C	171.5, C	172.8, C	171.8, C
2	54.0, CH	53.6, CH	53.6, CH	54.0, CH
3	30.0, CH	28.7, CH	30.0, CH	29.2, CH
4 ^A^	18.4, CH_3_	17.5, CH_3_	18.9, CH_3_	19.3, CH_3_
5 ^A^	18.9, CH_3_	19.3, CH_3_	18.2, CH_3_	18.0, CH_3_
NHCOCH_3_	169.1, C	170.6, C	168.8, C	170.2, C
NHCOCH_3_	22.2, CH_3_	22.2, CH_3_	22.1, CH_3_	21.7, CH_3_

^A^ Assignments with same superscript are interchangeable.

**Table 3:** 1D NMR (600 MHz, CDCl_3_) data for aflaquinolones H–I (**11**–**12**).

Position	11		12	
	*δ*_H_, Mult (*J* in Hz)	*δ*_C_, Type	*δ*_H_, Mult (*J* in Hz)	*δ*_C_, Type
2	-	165.8, C	-	165.4, C
3	3.70, br d (1.5)	84.3, CH	3.69, br d (1.4)	84.4, CH
3-OMe	3.61, s	59.1, C	3.60, s	59.0, C
4	-	78.9, C	-	78.9, C
5	-	111.1, C	-	111.0, C
6	-	155.2, C	-	155.0, C
7	-	122.4, C	-	123.0, C
8	7.40, d (8.2)	127.3, CH	7.37, d (8.1)	127.1, CH
9	6.36, d (8.2)	107.0, CH	6.32, d (8.1)	106.8, CH
10	-	134.4, C	-	134.0, C
11	-	129.2, C	-	129.2, C
12	7.18, d (8.8)	128.0, CH	7.18, d (8.8)	128.0, CH
13	6.82, d (8.8)	114.5, CH	6.83, d (8.8)	114.5, CH
14	-	160.5, C	-	160.4, C
14-OMe	3.76, s	55.4, CH_3_	3.76, s	55.5, CH_3_
15	6.82, d (8.8)	114.5, CH	6.83, d (8.8)	114.5, CH
16	7.18, d (8.8)	128.0, CH	7.18, d (8.8)	128.0, CH
17	6.79, d (16.6)	122.7, CH	6.61, d (16.8)	121.8, CH
18	6.28, d (16.6)	135.9, CH	6.14, d (16.8)	137.7, CH
19	-	37.6, C	-	37.2, C
20	*a.* 2.11, m	47.7, CH_2_	*a*. 1.74, m	46.1, CH_2_
	*b.* 1.47, dd (13.4, 13.4)		*b*. 1.07, m	
21	2.54, m	41.5, CH	1.57 ^A^	36.3, CH
22	-	214.0, C	3.12, ddd (10.5, 10.5, 4.5)	77.3, CH
23	*a.* 2.49, dd (14.2, 5.7)	38.7, CH_2_	*a.* 1.77, m	32.1, CH_2_
	*b.* 2.24, m		*b.* 1.51 ^A^	
24	*a.* 2.15, m	38.5, CH_2_	*a.* 1.82, m	36.9, CH_2_
	*b.* 1.73, td (13.4, 4.4)		*b.* 1.37, td (13.7, 3.5)	
25	1.10, s	30.6, CH_3_	1.03, s	31.3, CH_3_
26	0.99, d (6.5)	14.6, CH_3_	0.98, d (6.4)	18.8, CH_3_
1-NH	7.75, br s	-	7.35, br s	-
4-OH	4.58, s	-	4.54, s	-
6-OH	9.14, s	-	9.08, s	-

^A^ Resonances obscured by solvent, observed by HSQC.

**Table 4:** 1D NMR (methanol-*d*_4_) data for aspulvinone Y (**14**).

Position	14	
	δ_H_, Mult (*J* in Hz)	δ_C_, Type
1	-	ND
2	-	101.6
3	-	164.2
4	-	142.3
5	6.38, s	108.0
1′	-	122.6
2′	7.64, d (1.5)	129.7
3′	-	121.7
4′	-	154.3
5′	6.73, d (8.3)	117.6
6′	7.62, dd (8.3, 1.5)	127.7
7′	2.83, t (6.8)	23.1
8′	1.84, t (6.8)	33.5
9′	-	75.2
10′	1.33, s	26.8
11′	1.33, s	26.8
1″	-	126.3
2″	7.57, s	133.1
3″	-	121.4
4″	-	155.0
5″	6.78, d (8.6)	118.1
6″	7.51, d (1.5)	130.9
7″	*a*. 3.07, dd (16.4, 5.0)	31.8
	*b*. 2.78, dd (16.4, 7.3)	
8″	3.79, dd (7.3, 5.0)	69.9
9″	-	78.5
10″	1.29, s	21.1
11″	1.35, s	25.6

ND: Not detected due to low intensity.

## Data Availability

Raw NMR data have been deposited in NP-MRD (The Natural Products Magnetic Resonance Database).

## Supplementary Materials

The following supporting information can be downloaded at: https://www.mdpi.com/article/10.3390/molecules27249066/s1, Figures S1–S62 and Tables S1–S11 NMR spectra, tabulated NMR data and X-ray crystallographic data.

## Sample Availability

Samples of selected compounds may be available from the authors on request, subject to an approved Materials Transfer Agreement.

## References

[1] Capon R.J., Skene C., Stewart M., Ford J., O’Hair R.A.J., Williams L., Lacey E., Gill J.H., Heiland K., Friedel T. (2003). Aspergillicins A–E: Five Novel Depsipeptides from the Marine-Derived Fungus *Aspergillus carneus*. Org. Biomol. Chem..

[2] Capon R.J., Ratnayake R., Stewart M., Lacey E., Tennant S., Gill J.H. (2005). Aspergillazines A-E: Novel Heterocyclic Dipeptides from an Australian Strain of *Aspergillus unilateralis*. Org. Biomol. Chem..

[3] Fremlin L.J., Piggott A.M., Lacey E., Capon R.J. (2009). Cottoquinazoline A and Cotteslosins A and B, Metabolites from an Australian Marine-Derived Strain of *Aspergillus versicolor*. J. Nat. Prod..

[4] Khalil Z.G., Huang X.-C., Raju R., Piggott A.M., Capon R.J. (2014). Shornephine A: Structure, Chemical Stability, and P-Glycoprotein Inhibitory Properties of a Rare Diketomorpholine from an Australian Marine-Derived *Aspergillus* sp.. J. Org. Chem..

[5] Quezada M., Shang Z., Kalansuriya P., Salim A.A., Lacey E., Capon R.J. (2017). Waspergillamide A, a Nitro Depsi-Tetrapeptide Diketopiperazine from an Australian Mud Dauber Wasp-Associated *Aspergillus* sp. (CMB-W031). J. Nat. Prod..

[6] Aron A.T., Gentry E.C., McPhail K.L., Nothias L.-F., Nothias-Esposito M., Bouslimani A., Petras D., Gauglitz J.M., Sikora N., Vargas F. (2020). Reproducible Molecular Networking of Untargeted Mass Spectrometry Data Using GNPS. Nat. Protoc..

[7] Salim A.A., Khalil Z.G., Elbanna A.H., Wu T., Capon R.J. (2021). Methods in Microbial Biodiscovery. Mar. Drugs.

[8] Wu T., Salim A.A., Capon R.J. (2021). Millmerranones A-F: A Meroterpene Cyclic Carbonate and Related Metabolites from the Australian Fungus *Aspergillus* sp. CMB-MRF324. Org. Lett..

[9] Alvi K.A., Pu H., Luche M., Rice A., App H., McMahon G., Dare H., Margolis B. (1999). Asterriquinones Produced by *Aspergillus candidus* Inhibit Binding of the Grb-2 Adapter to Phosphorylated E Receptor Tyrosine Kinase. J. Antibiot..

[10] Kaji A., Iwata T., Kiriyama N., Wakusawa S., Miyamoto K.-I. (1994). Four New Metabolites of *Aspergillus terreus*. Chem. Pharm. Bull..

[11] Mocek U., Schultz L., Buchan T., Baek C., Fretto L., Nzerem J., Sehl L., Sinha U. (1996). Isolation and Structure Elucidation of Five New Asterriquinones from *Aspergillus*, *Humicola* and *Botryotrichum* Species. J. Antibiot..

[12] Nakagawa M., Hirota A., Sakai H., Isogai A. (1982). Terrecyclic Acid A, a New Antibiotic from *Aspergillus terreus*. I. Taxonomy, Production, and Chemical and Biological Properties. J. Antibiot..

[13] Hirota A., Nakagawa M., Sakai H., Isogai A. (1982). Terrecyclic Acid A, a New Antibiotic from *Aspergillus terreus*. II. Structure of Terrecyclic Acid A. J. Antibiot..

[14] Hirota A., Nakagawa M., Sakai H., Isogai A. (1984). Terrecyclic Acid A, a New Antibiotic from *Aspergillus terreus*. III. Carbon-13 NMR Spectrum of Terrecyclic Acid A. J. Antibiot..

[15] Hirota A., Nakagawa M., Hirota H., Takahashi T., Isogai A. (1986). Terrecyclic Acid A, a New Antibiotic from *Aspergillus terreus*. IV. Absolute Stereochemistry of Terrecyclic Acid A. J. Antibiot..

[16] Cruz P.G., Auld D.S., Schultz P.J., Lovell S., Battaile P., MacArthur R., Shen M., Tamayo-Castillo G., Inglese J., Sherman D.H. (2011). Titration-Based Screening for Evaluation of Natural Product Extracts: Identification of an Aspulvinone Family of Luciferase Inhibitors. Chem. Biol..

[17] Ojima N., Takenaka S., Seto S. (1973). New Butenolides from *Aspergillus terreus*. Phytochemistry.

[18] Ojima N., Takenaka S., Seto S. (1975). Structures of Pulvinone Derivatives from *Aspergillus terreus*. Phytochemistry.

[19] Ojima N., Takahashi I., Ogura K., Seto S. (1976). New Metabolites from *Aspergillus terreus* Related to the Biosynthesis of Aspulvinones. Tetrahedron Lett..

[20] Capon R.J. (2020). Extracting Value: Mechanistic Insights into the Formation of Natural Product Artifacts—Case Studies in Marine Natural Products. Nat. Prod. Rep..

[21] Kagamizono T., Sakai N., Arai K., Kobinata K., Osada H. (1997). Terpeptin, a Novel Mammalian Cell Cycle Inhibitor, Produced by *Aspergillus terreus* 95F-1. Tetrahedron Lett..

[22] Toske S.G., Jensen P.R., Kauffman C.A., Fenical W. (1998). Aspergillamides A and B: Modified Cytotoxic Tripeptides Produced by a Marine Fungus of the Genus *Aspergillus*. Tetrahedron.

[23] Shiomi K., Hatae K., Yamaguchi Y., Masuma R., Tomoda H., Kobayashi S., Omura S. (2002). New Antibiotics Miyakamides Produced by a Fungus. J. Antibiot..

[24] Lin Z., Zhu T., Fang Y., Gu Q. (2008). ^1^H and ^13^C NNMR Assignments of Two New Indolic Enamide Diastereomers from a Mangrove Endophytic Fungus *Aspergillus* sp.. Magn. Reson. Chem..

[25] Izumikawa M., Hashimoto J., Takagi M., Shin-ya K. (2010). Isolation of Two New Terpeptin Analogs—JBIR-81 and JBIR-82—From a Seaweed-Derived Fungus, *Aspergillus* sp. SpD081030G1f1. J. Antibiot..

[26] Luo X.W., Lin Y., Lu Y.-J., Zhou X.-F., Liu Y.-H. (2019). Peptides and Polyketides Isolated from the Marine Sponge-Derived Fungus *Aspergillus terreus* SCSIO 41008. Chin. J. Nat. Med..

[27] Hooft R.W.W., Straver L.H., Spek A.L. (2008). Determination of Absolute Structure Using Bayesian Statistics on Bijvoet Differences. J. Appl. Crystallogr..

[28] Neff S.A., Lee S.U., Asami Y., Ahn J.S., Oh H., Baltrusaitis J., Gloer J.B., Wicklow D.T. (2012). Aflaquinolones A-G: Secondary Metabolites from Marine and Fungicolous Isolates of *Aspergillus* spp.. J. Nat. Prod..

[29] Mou X.-F., Liu X., Xu R.-F., Wei M.-Y., Fang Y.W., Shao C.-L. (2018). Scopuquinolone B, a New Monoterpenoid Dihydroquinolin-2(1H)-One Isolated from the Coral-Derived *Scopulariopsis* sp. Fungus. Nat. Prod. Rep..

[30] Chen M., Shao C.-L., Meng H., She Z.-G., Wang C.-Y. (2014). Anti-Respiratory Syncytial Virus Prenylated Dihydroquinolone Derivatives from the Gorgonian-Derived Fungus *Aspergillus* sp. XS-20090B15. J. Nat. Prod..

[31] Wu C., Cui X., Sun L., Lu J., Li F., Song M., Zhang Y., Hao X., Tian C., Song M. (2021). Aspulvinones Suppress Postprandial Hyperglycemia as Potent A-Glucosidase Inhibitors from *Aspergillus terreus* ASM-1. Front. Chem..

[32] Elyashberg M., Blinov K., Molodtsov S., Williams A.J. (2013). Structure Revision of Asperjinone Using Computer-Assisted Structure Elucidation Methods. J. Nat. Prod..

[33] Begley M.J., Gedge D.R., Knight D.W., Pattenden G. (1979). Aspulvinones, a New Class of Natural Products from *Aspergillus terreus*. Re-Investigation of Structures by X-Ray Crystallographic and Spectroscopic Analysis. J. Chem. Soc., Perkin Trans..

[34] Gao H., Guo W., Wang Q., Zhang L., Zhu M., Zhu T., Gu Q., Wang W., Li D. (2013). Aspulvinones from a Mangrove Rhizosphere Soil-Derived Fungus *Aspergillus terreus* Gwq-48 with Anti-Influenza A Viral (H1N1) Activity. Bioorg. Med. Chem. Lett..

[35] Guo C.-J., Knox B.P., Sanchez J.F., Chiang Y.-M., Bruno K.S., Wang C.C.C. (2013). Application of an Efficient Gene Targeting System Linking Secondary Metabolites to Their Biosynthetic Genes in *Aspergillus terreus*. Org. Lett..

[36] Zhang P., Li X.-M., Wang J.-N., Li X., Wang B.-G. (2015). New Butenolide Derivatives from the Marine-Derived Fungus *Paecilomyces variotii* with DPPH Radical Scavenging Activity. Phytochem. Lett..

[37] Zhang L.-H., Feng B.-M., Zhao Y.-Q., Sun Y., Liu B., Liu F., Chen G., Bai J., Hua H.-M., Wang H.-F. (2016). Polyketide Butenolide, Diphenyl Ether, and Benzophenone Derivatives from the Fungus *Aspergillus flavipes* PJ03-11. Bioorg. Med. Chem. Lett..

[38] Pang X., Zhao J.-Y., Fang X.-M., Zhang T., Zhang D.-W., Liu H.-Y., Su J., Cen S., Yu L.-Y. (2017). Metabolites from the Plant Endophytic Fungus *Aspergillus* sp. CPCC 400735 and Their Anti-HIV Activities. J. Nat. Prod..

[39] Machado F.P., Kumla D., Pereira J.A., Sousa E., Dethoup T., Freitas-Silva J., Costa P.M., Mistry S., Silva A.M.S., Kijjoa A. (2021). Prenylated Phenylbutyrolactones from Cultures of a Marine Sponge-Associated Fungus *Aspergillus flavipes* KUFA1152. Phytochemistry.

[40] Liang X.-X., Zhang X.-J., Zhao Y.-X., Feng J., Zeng J.-C., Shi Q.-Q., Kaunda J.S., Li X.-L., Wang W.-G., Xiao W.-L. (2022). Aspulvins A–H, Aspulvinone Analogues with SARS-CoV-2 Mpro Inhibitory and Anti-Inflammatory Activities from an Endophytic *Cladosporium* sp.. J. Nat. Prod..

[41] Hsiao G., Chi W.-C., Chang C.-H., Chiang Y.-R., Fu Y.-J., Lee T.-H. (2022). Bioactive Pulvinones from a Marine Algicolous Fungus *Aspergillus Terreus* NTU243. Phytochemistry.

[42] Manchoju A., Annadate R.A., Desquien L., Pansare S.V. (2018). Functionalization of Diazotetronic Acid and Application in a Stereoselective Modular Synthesis of Pulvinone, Aspulvinones A–E, G, Q and Their Analogues. Org. Biomol. Chem..

[43] Chen G.-Y., Ruan B.-H., Yang Y.-B., Wang Q., Li X.-Z., Luo N., Yang X.-Q., Zhao L.-X. (2018). Secondary Metabolites of the Fungus *Aspergillus ferreus*. Chem. Nat. Compd..

[44] Sheldrick G.M. (2008). A Short History of Shelx. Acta Crystallogr., Sect. A Found. Crystallogr..

[45] Macrae C.F., Edgington P.R., McCabe P., Pidcock E., Shields G.P., Taylor R., Towler M., van de Streek J. (2006). Mercury: Visualization and Analysis of Crystal Structures. J. Appl. Crystallogr..

[46] Farrugia L.J. (1999). Wingx Suite for Small-Molecule Single-Crystal Crystallography. J. Appl. Crystallogr..

