# Supplementary material for: Molecular Networking and Cultivation Profiling Reveals Diverse Natural Product Classes from an Australian Soil-Derived Fungus Aspergillus sp. CMB-MRF324
Source: Molecules. 2022 Dec 19;27(24):9066. doi: 10.3390/molecules27249066 (PMC9786664; doi:10.3390/molecules27249066)
Supplement: Supplementary file 1 [file molecules-27-09066-s001.zip › molecules-2054000-supplementary.pdf]

Supplementary Materials

Molecular Networking and Cultivation Profiling Reveals Diverse Natural Product Classes from an Australian Soil-Derived Fungus *Aspergillus* sp. CMB-MRF324

Taizong Wu[1], Angela A. Salim[1], Paul V. Bernhardt[2] and Robert J. Capon[1,*]

[1] Institute for Molecular Bioscience, The University of Queensland, Brisbane, QLD 4072, Australia
[2] School of Chemistry and Molecular Bioscience, The University of Queensland, Brisbane, QLD 4072, Australia

* Correspondence: r.capon@uq.edu.au; Tel. +61-7-3346-2979

Table of Contents

1. Isolation schemes for pure compounds 5

2. Transformation of **8** to **7** with exposure to heat 6

3. Marfey's analysis for aspergillamides E-F (**7**-**8**) 6

4 Spectroscopic data of **7**-**18** 7

4.1 Aspergillamide E (**7a**-**7b**) 7

4.2 Aspergillamide F (**8a**-**8b**) 13

4.3 Asterriquinone SU5228 (**9**) 19

4.4 Asterriquinone CT5 (**10**) 21

4.5 Aflaquinolone H (**11**) 25

4.6 Aflaquinolone I (**12**) 30

4.7 Terrecyclic acid A (**13**) 35

4.8 Aspulvinone Y (**14**) 38

4.9 Aspulvinone N-CR (**15**) 42

4.10 Aspulvinone B (**16**) 45

4.11 Aspulvinone D (**17**) 46

4.12 Aspulvinone H (**18**) 47

5. X-ray Crystallography of Aflaquinolone H (**11**) 48

6. MS/MS fragmentation analysis for aspergillamides related nodes 54

List of Tables

Table S1. 1D and 2D NMR (600 MHz, DMSO-d_6) data for aspergillamide E (**7a**) 7

Table S2. 1D and 2D NMR (600 MHz, DMSO-d_6) data for aspergillamide E (**7b**) 8

Table S3. 1D and 2D NMR (600 MHz, DMSO-d_6) data for aspergillamide F (**8a**) 13

Table S4. 1D and 2D NMR (600 MHz, DMSO-d_6) data for aspergillamide F (**8b**) 14

Table S5. 1D and 2D NMR (600 MHz, DMSO-d_6) data for asterriquinone SU5228 (**9**) 19

Table S6. 1D and 2D NMR (600 MHz) data for asterriquinone CT5 (**10**) 21

Table S7. 1D and 2D NMR (600 MHz, $CDCl_3$) data for aflaquinolone H (**11**) 25

Table S8. 1D and 2D NMR (600 MHz, $CDCl_3$) data for aflaquinolone I (**12**) 30

Table S9. 1D and 2D NMR (600 MHz, DMSO-d_6) data for terrecyclic acid A (**13**) 35

Table S10. 1D and 2D NMR (600 MHz, methanol-d_4) data for aspulvinone Y (**14**) 38

Table S11. 1D and 2D NMR (600 MHz, acetone-d_6) data for aspulvinone N-CR (**15**)* 42

List of Figures

Figure S1. Isolation scheme for compounds **7-10** from CMB-MRF324 SDA culture. 5

Figure S2. Isolation scheme for compounds **11-18** from CMB-MRF324 brown rice culture. 5

Figure S3. Transformation from **8** to **7** when exposed to heat (60 °C). 6

Figure S4. Marfey's analysis for **7-8**: DAD (340 nm) chromatogram of L-FDAA amino acid derivatives of acid hydrolysate of an aliquot of **7** (i); **8** (ii); and standard D-NMe-Phe-L-FDAA derivative (iii), standard D-NMe-Phe-D-FDAA derivative (iv); standard D-Val-L-FDAA derivative (v); standard D-Val-L-FDAA derivative (vi). 6
Figure S5. ^{1}H NMR (600 MHz, DMSO-d_6) spectrum of aspergillamide E (**7a**, labelled in black; **7b**, labelled in red). 9
Figure S6. ^{13}C NMR (150 MHz, DMSO-d_6) spectrum of aspergillamide E (**7a**, labelled in black; **7b**, labelled in red). 9
Figure S7. HSQC NMR **(**600 MHz, DMSO-d_6) spectrum of aspergillamide E (**7**). 10
Figure S8. HMBC NMR **(**600 MHz, DMSO-d_6) spectrum of aspergillamide E (**7**). 10
Figure S9. COSY NMR **(**600 MHz, DMSO-d_6) spectrum of aspergillamide E (**7**). 11
Figure S10. ROESY NMR **(**600 MHz, DMSO-d_6) spectrum of aspergillamide E (**7**). 11
Figure S11. HRMS spectrum and measurement for aspergillamide E (**7**). 12
Figure S12. ^{1}H NMR (600 MHz, DMSO-d_6) spectrum of aspergillamide F (**8a**, labelled in black; **8b**, labelled in red). 15
Figure S13. ^{13}C NMR (150 MHz, DMSO-d_6) spectrum of aspergillamide F (**8a**, labelled in black; **8b**, labelled in red). 15
Figure S14. HSQC NMR **(**600 MHz, DMSO-d_6) spectrum of aspergillamide F (**8**). 16
Figure S15. HMBC NMR **(**600 MHz, DMSO-d_6) spectrum of aspergillamide F (**8**). 16
Figure S16. COSY NMR **(**600 MHz, DMSO-d_6) spectrum of aspergillamide F (**8**). 17
Figure S17. HMBC NMR **(**600 MHz, DMSO-d_6) spectrum of aspergillamide F (**8**). 17
Figure S18. HRMS spectrum and measurement for aspergillamide F (**8**). 18
Figure S19. ^{1}H NMR (600 MHz, DMSO-d_6) spectrum of asterriquinone SU5228 (**9**). 20
Figure S20. HRMS spectrum and measurement for asterriquinone SU5228 (**9**). 20
Figure S21. ^{1}H NMR (600 MHz, DMSO-d_6) spectrum of asterriquinone CT5 (**10**). 22
Figure S22. ^{1}H NMR (600 MHz, methanol-d_4) spectrum of asterriquinone CT5 (**10**). 22
Figure S23. ^{1}H NMR (600 MHz, $CDCl_3$) spectrum of asterriquinone CT5 (**10**). 23
Figure S24. ^{13}C NMR (150 MHz, $CDCl_3$) spectrum of asterriquinone CT5 (**10**). 23
Figure S25. HRMS spectrum and measurement for asterriquinone CT5 (**10**). 24
Figure S26. ^{1}H NMR (600 MHz, $CDCl_3$) spectrum for aflaquinolone H (**11**). 26
Figure S27. ^{13}C NMR (150 MHz, $CDCl_3$) and UV-vis (inset) spectra for aflaquinolone H (**11**). ... 26
Figure S28. HSQC NMR ($CDCl_3$) spectrum for aflaquinolone H (**11**). 27
Figure S29. HMBC NMR ($CDCl_3$) spectrum for aflaquinolone H (**11**). 27
Figure S30. COSY NMR ($CDCl_3$) spectrum for aflaquinolone H (**11**). 28
Figure S31. ROESY NMR ($CDCl_3$) spectrum for aflaquinolone H (**11**). 28

Figure S32. HRMS spectrum and measurement for aflaquinolone H (**11**). 29
Figure S33. ^{1}H NMR (600 MHz, $CDCl_3$) spectrum for aflaquinolone I (**12**)................................. 31
Figure S34. ^{13}C NMR (150 MHz, $CDCl_3$) and UV-vis (inset) spectra for aflaquinonlone I (**12**). ... 31
Figure S35. HSQC NMR ($CDCl_3$) spectrum for aflaquinolone I (**12**). 32
Figure S36. HMBC NMR ($CDCl_3$) spectrum for aflaquinolone I (**12**). 32
Figure S37. COSY NMR ($CDCl_3$) spectrum for aflaquinolone I (**12**). 33
Figure S38. ROESY NMR ($CDCl_3$) spectrum for aflaquinolone I (**12**). 33
Figure S39. HRMS spectrum and measurement for aflaquinolone I (**12**). 34
Figure S40. ^{1}H NMR (600 MHz, DMSO-d_6) spectrum for terrecyclic acid A (**13**). 36
Figure S41. ^{13}C NMR (150 MHz, DMSO-d_6) spectrum for terrecyclic acid A (**13**). 36
Figure S42. HRMS spectrum and measurement for terrecyclic acid A (**13**). 37
Figure S43. ^{1}H NMR (600 MHz, acetone-d_6) spectrum for aspulvinone Y (**14**). 39
Figure S44. HSQC NMR (600 MHz, acetone-d_6) spectrum for aspulvinone Y (**14**). 39
Figure S45. HMBC NMR (600 MHz, acetone-d_6) spectrum for aspulvinone Y (**14**). 40
Figure S46. COSY NMR (600 MHz, acetone-d_6) spectrum for aspulvinone Y (**14**). 40
Figure S47. HRMS spectrum and measurement for aspulvinone Y (**14**). 41
Figure S48. ^{1}H NMR (600 MHz, acetone-d_6) spectrum for aspulvinone N-CR (**15**). 43
Figure S49. HSQC NMR (600 MHz, acetone-d_6) spectrum for aspulvinone N-CR (**15**). 43
Figure S50. HMBC NMR (600 MHz, acetone-d_6) spectrum for aspulvinone N-CR (**15**). 44
Figure S51. HRMS spectrum and measurement for aspulvinone N-CR (**15**). 44
Figure S52. ^{1}H NMR (600 MHz, acetone-d_6) spectrum for aspulvinone B (**16**). 45
Figure S53. HRMS spectrum and measurement for aspulvinone B (**16**). 45
Figure S54. ^{1}H NMR (600 MHz, acetone-d_6) spectrum for aspulvinone D (**17**). 46
Figure S55. HRMS spectrum and measurement for aspulvinone D (**17**). 46
Figure S56. ^{1}H NMR (600 MHz, methanol-d_4) spectrum for aspulvinone H (**18**). 47
Figure S57. HRMS spectrum and measurement for aspulvinone H (**18**). 47
Figure S58. MS/MS fragmentation and HRMS analysis for aspergillamides E-F (**7-8**). 54
Figure S59. MS/MS fragmentation and HRMS analysis for aspergillamides A-D (**20-21**, **30-31**). 54
Figure S60. MS/MS fragmentation and HRMS analysis for aspergillamide(s) **i**. 55
Figure S61. MS/MS fragmentation and HRMS analysis for aspergillamide(s) **ii**. 55
Figure S62. Chemical shifts of C-8″ and C-9″ for dihydrobenzofuran (yellow highlight) and dihydrobenzopyran (green highlight) moiety 56

1. Isolation schemes for pure compounds

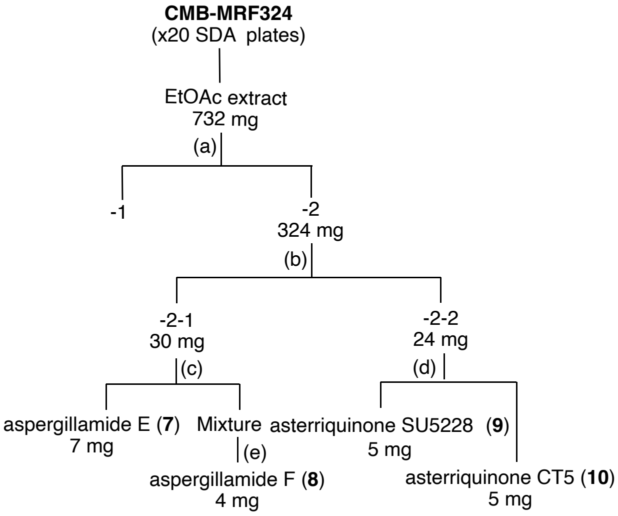

(a) Trituration [*n*-hexane (-1), MeOH (-2)]
(b) HPLC; column prep C_8, 40-75% MeCN (0.01 % TFA) over 25 mins, 20 mL/min
(c) HPLC; column semi-prep C_8, 45% MeCN (0.01 % TFA) over 20 mins, 20 mL/min
(d) HPLC; column semi-prep C_8, 60-75% MeCN (0.01 % TFA) over 15 mins, 20 mL/min
(e) HPLC; column semi-prep C_8, 40-45% MeCN (no acid) over 25 mins, 20 mL/min

Figure S1. Isolation scheme for compounds **7-10** from CMB-MRF324 SDA culture.

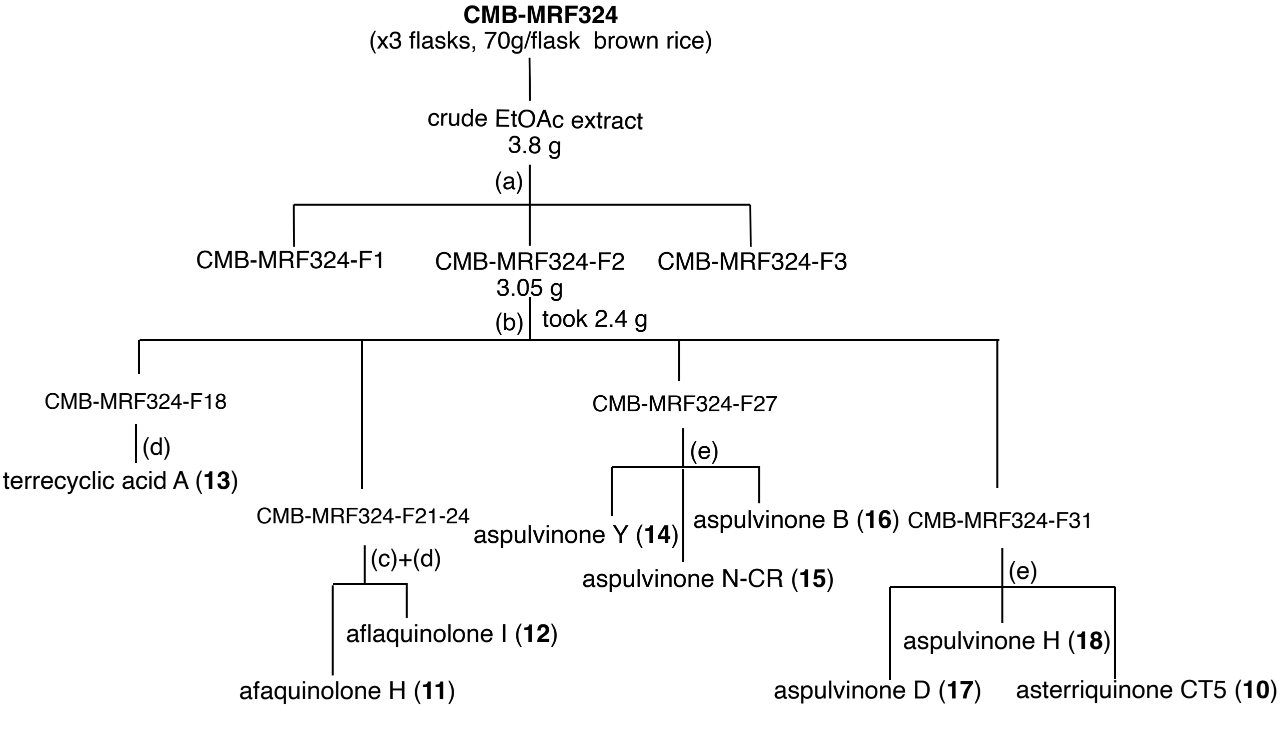

(a) Trituration [*n*-hexane (F1), DCM (F2), MeOH (F3)
(b) HPLC; column semi-prep C_8, 10-100% MeCN (0.01 % TFA) in 30 mins, 20 mL/min
(c) Sephadex LH20, MeOH/DCM = 1:1
(d) HPLC; column semiprep C_3, 40-45% MeCN (0.01% TFA) in 20 mins, 3 mL/min
(e) HPLC; column semiprep C_8, 55% MeCN (0.01% TFA) in 20 mins, 3 mL/min

Figure S2. Isolation scheme for compounds **11-18** from CMB-MRF324 brown rice culture.

2. Transformation of 8 to 7 with exposure to heat

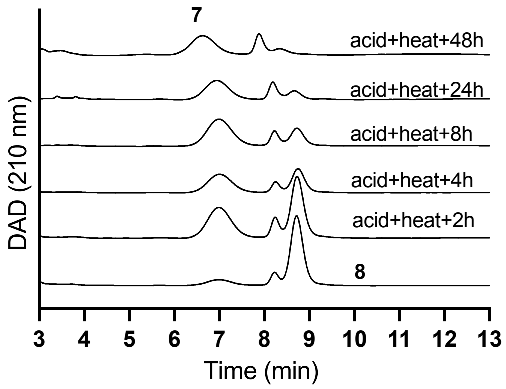

Figure S3. Transformation from **8** to **7** when exposed to heat (60 °C).

3. Marfey's analysis for aspergillamides E-F (7-8)

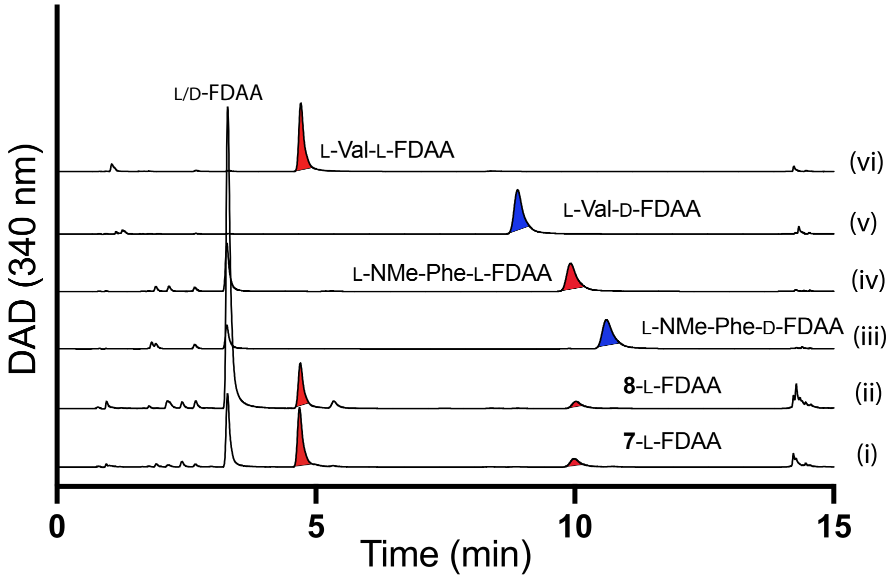

Figure S4. Marfey's analysis for **7-8**: DAD (340 nm) chromatogram of L-FDAA amino acid derivatives of acid hydrolysate of an aliquot of **7** (i); **8** (ii); and standard D-NMe-Phe-L-FDAA derivative (iii), standard D-NMe-Phe-D-FDAA derivative (iv); standard D-Val-L-FDAA derivative (v); standard D-Val-L-FDAA derivative (vi).

4 Spectroscopic data of 7-18

4.1 Aspergillamide E (7a-7b)

Table S1. 1D and 2D NMR (600 MHz, DMSO-d_6) data for aspergillamide E (**7a**)

	Pos.	δ_H, mult, (*J* in Hz)	δ_C	COSY	ROESY	HMBC
enamino-Trp	2	7.42, d (2.2)	123.5	1-NH	8, 1-NH	7a, 3, 3a, 8
	3		111.5			
	3a		124.8			
	4	7.61, d (7.7)	118.9	5		7a, 3, 3a, 6
	5	7.07, dd (7.7, 7.7)	119.3	4		3a, 7
	6	7.12, m	121.4	7		7a, 4
	7	7.38, d (8.0)	111.8	6		3a, 5
	7a		136.8			
	8	6.42, d (15.0)	106.8	9	2, 9-NH	2, 3a, 9
	9	7.27, dd (15.0, 9.9)	119.5	8, 9-NH		3, 8, 10
	1-NH	11.1, d (2.2)		2	2	7a, 3, 3a
	9-NH	9.93, d (9.9)		9	8, Phe-2, Phe-3a	8, 10
N-Me-L-Phe	1		167.0			
	2	5.27, dd (9.6, 6.1)	57.7	2a, 3b	3a, 3b, 5, 9-NH	1, 3, 4, N-Me, Val-1
	3	a. 3.23, dd (14.3, 6.1)	34.1	2, 3b	2, 5, 9-NH	1, 2, 4, 5
		b. 2.97, dd (14.3, 9.6)		2, 3a	2, 5	2, 4, 5
	4		137.6			
	5	7.21, d (7.1)	128.8			3, 7
	6	7.24, dd (7.1, 7.1)	128.1			4
	7	7.18, t (7.1)	126.3			5
	N-Me	3.08, s	32.3		2, 5, Val-2, Val-4, Val-5	2, Val-1
L-Val	1		172.4			
	2	4.44, dd (8.7, 8.7)	54.0	3, NH	N-Me, 4, 5	3, 4, 5
	3	1.90, dqq (8.7, 6.9, 6.9)	30.0	2, 3, 4	NH	2, 4, 5
	4	0.81, d (6.9)	18.4	3	N-Me, 2	2, 3, 5
	5	0.84, d (6.9)	18.9	3	2, NH	2, 3, 4
	NH	7.97, d (8.7)		2	3, 4, 5, N-acetyl	2, 3, N-acetyl
	NHAc		169.1			
		1.79, s	22.2		Val-NH	

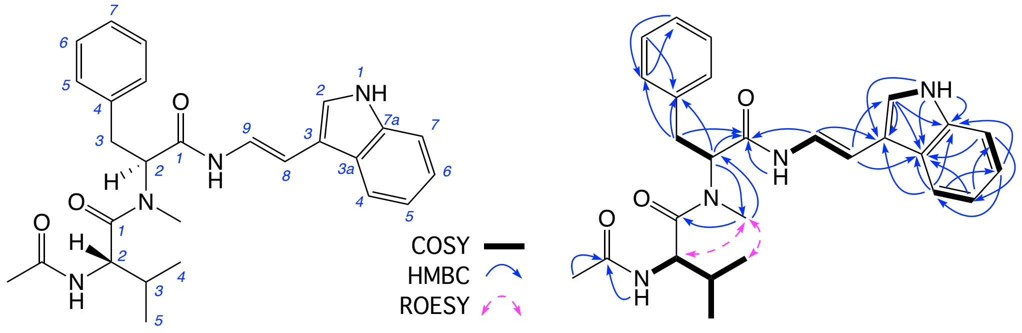

Table S2. 1D and 2D NMR (600 MHz, DMSO-d_6) data for aspergillamide E (**7b**)

	Pos.	δ_H, mult, (J in Hz)	δ_C	COSY	ROESY	HMBC
enamino-Trp	2	7.45, d (2.2)	123.6	1-NH	8, 1-NH	7a, 3, 3a
	3		111.4			
	3a		124.9			
	4	7.62, d (7.7)	118.8	5		7a, 3, 3a, 6
	5	7.11, m	119.3	4		
	6	7.07, m	121.4	7		
	7	7.36, d (8.0)	111.8	6		3a, 5
	7a		136.8			
	8	6.52, d (15.0)	107.0	9	2, 9-NH	2, 3a, 9
	9	7.32, dd (15.0, 9.9)	119.5	8		3, 8, Phe-1
	1-NH	11.1, d (2.2)		2	2	7a, 3, 3a
	9-NH	9.92, d (9.9)		9	8, Phe-2	8, Phe-1
N-Me-L-Phe	1		166.1			
	2	4.98, dd (9.6, 5.2)	61.3	3a, 3b	9-NH, 3a, 3b, 5, Val-2	1, 3, 4, N-Me, Val-[illegible]
	3	a. 3.32[A]	34.2			
		b. 2.95, d (14.3, 6.1)			2, 5	1, 2, 4, 5
	4		137.8			
	5	7.29, d (7.1)	129.3			
	6	7.30, dd (7.1, 7.1)	128.4			
	7	7.22, t (7.1)	126.5			
	N-Me	2.81, s	29.3			2, Val-1
Val	1		171.5			
	2	4.29, dd (7.6, 7.6)	53.6	3, NH	Phe-2, Phe-5, 3, 4, 5	3, 4, 5
	3	1.23, dqq (7.6, 6.9, 6.9)	28.7	2, 24/5	NH	2, 4, 5
	4	0.63, d (6.9)	17.5	3	2, NH	2, 3, 4
	5	0.48, d (6.9)	19.3	3	2, NH	2, 3, 4
	NH	8.26, d (7.6)		2	N-acetyl	2, N-acetyl
	NHAc		170.6			
		1.91, s	22.2		Val-NH	

[A] Resonance under H_2O peak, detected by HSQC.

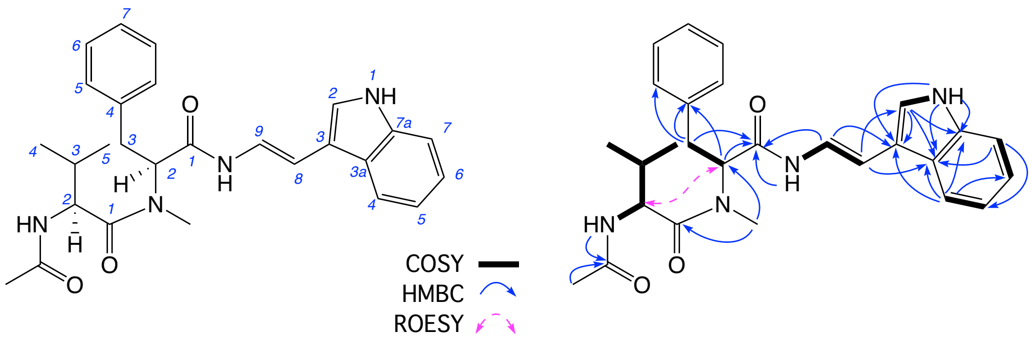

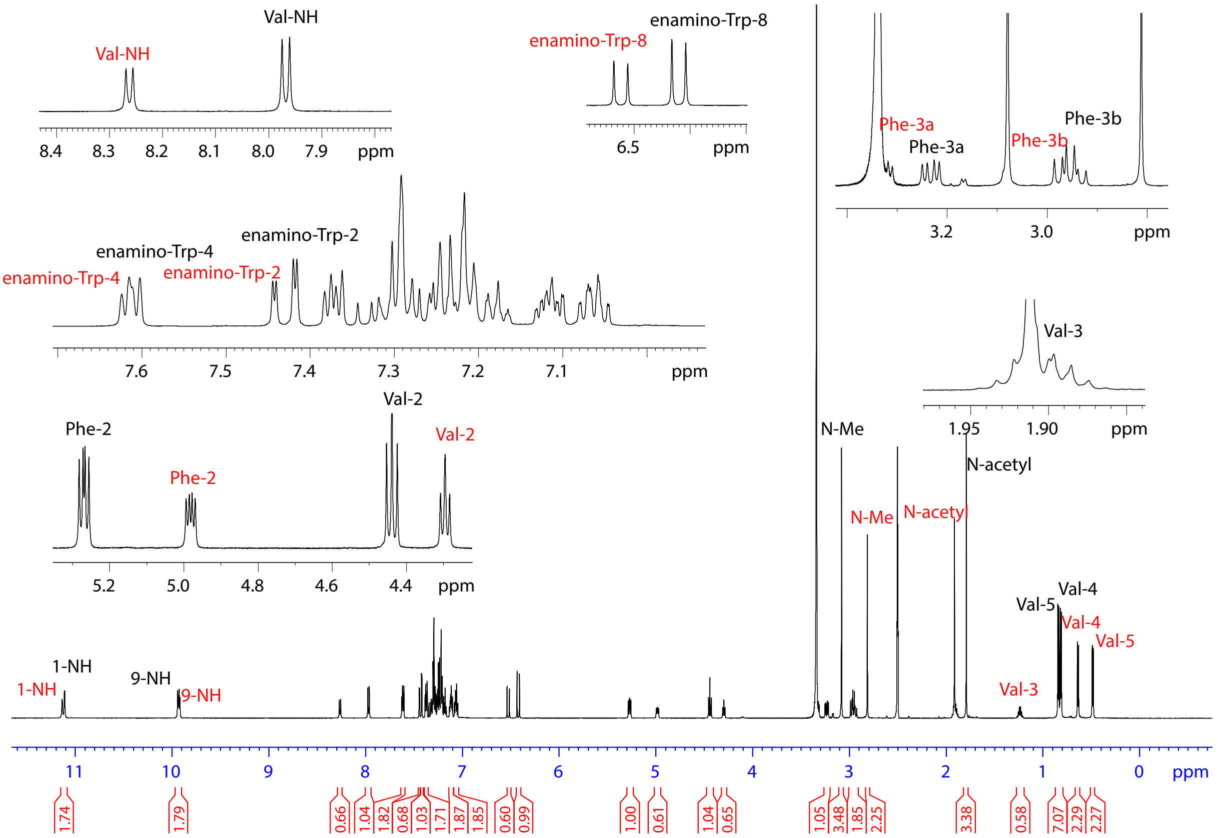

Figure S5. ^{1}H NMR (600 MHz, DMSO-d_6) spectrum of aspergillamide E (**7a**, labelled in black; **7b**, labelled in red).

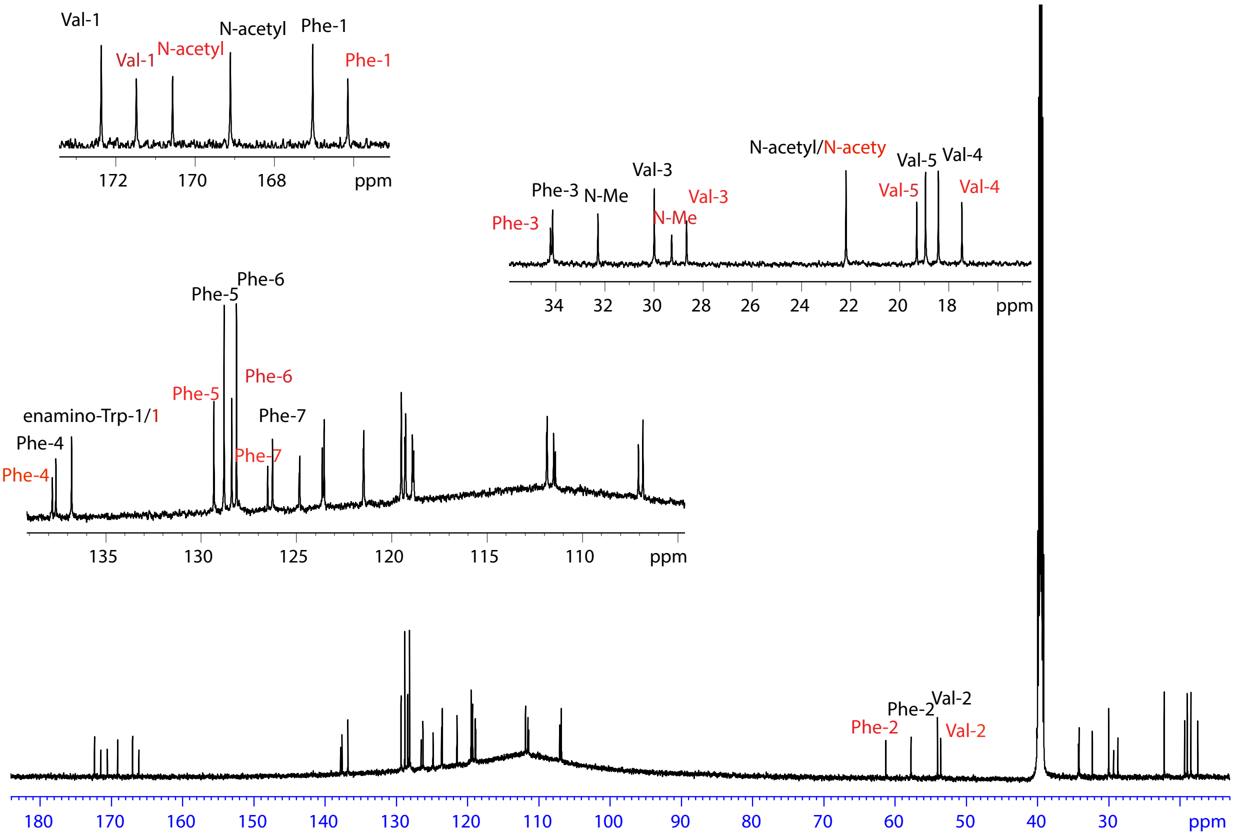

Figure S6. ^{13}C NMR (150 MHz, DMSO-d_6) spectrum of aspergillamide E (**7a**, labelled in black; **7b**, labelled in red).

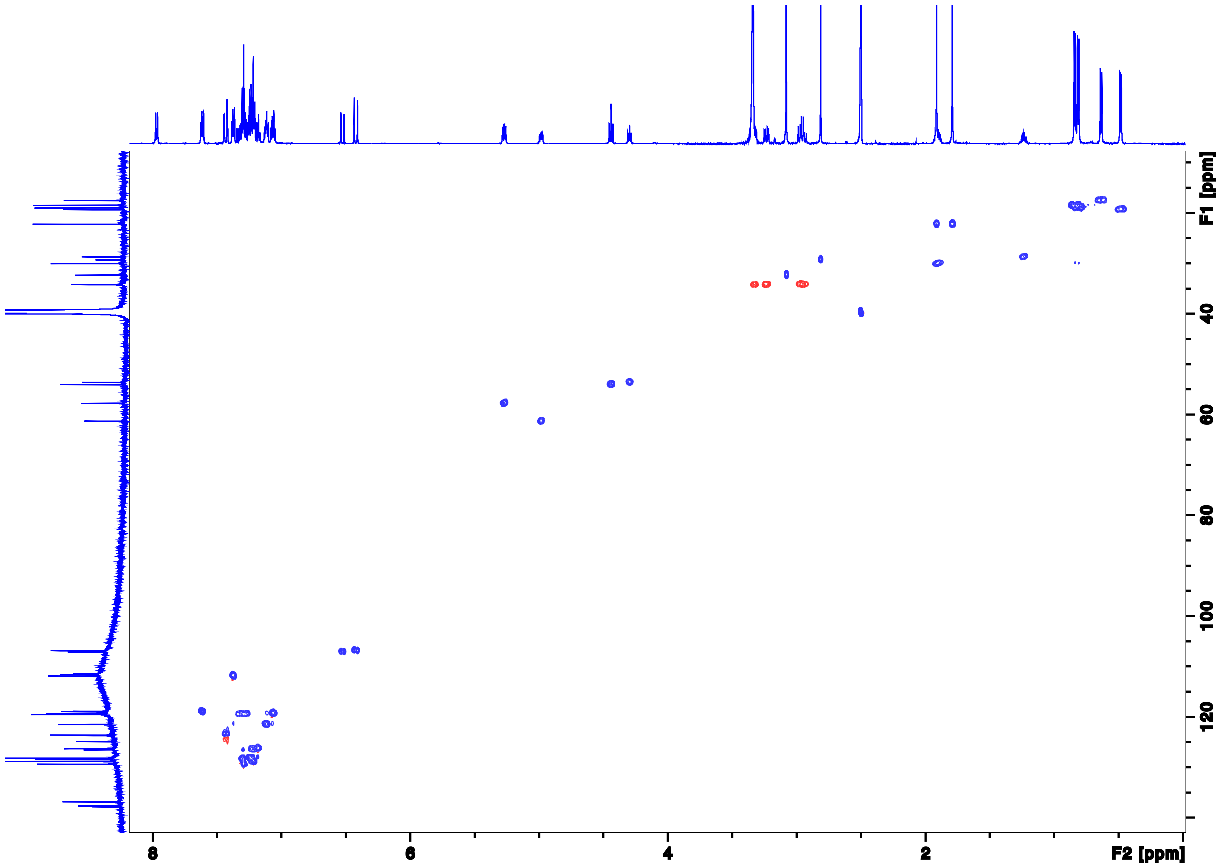

Figure S7. HSQC NMR (600 MHz, DMSO-d_6) spectrum of aspergillamide E (**7**).

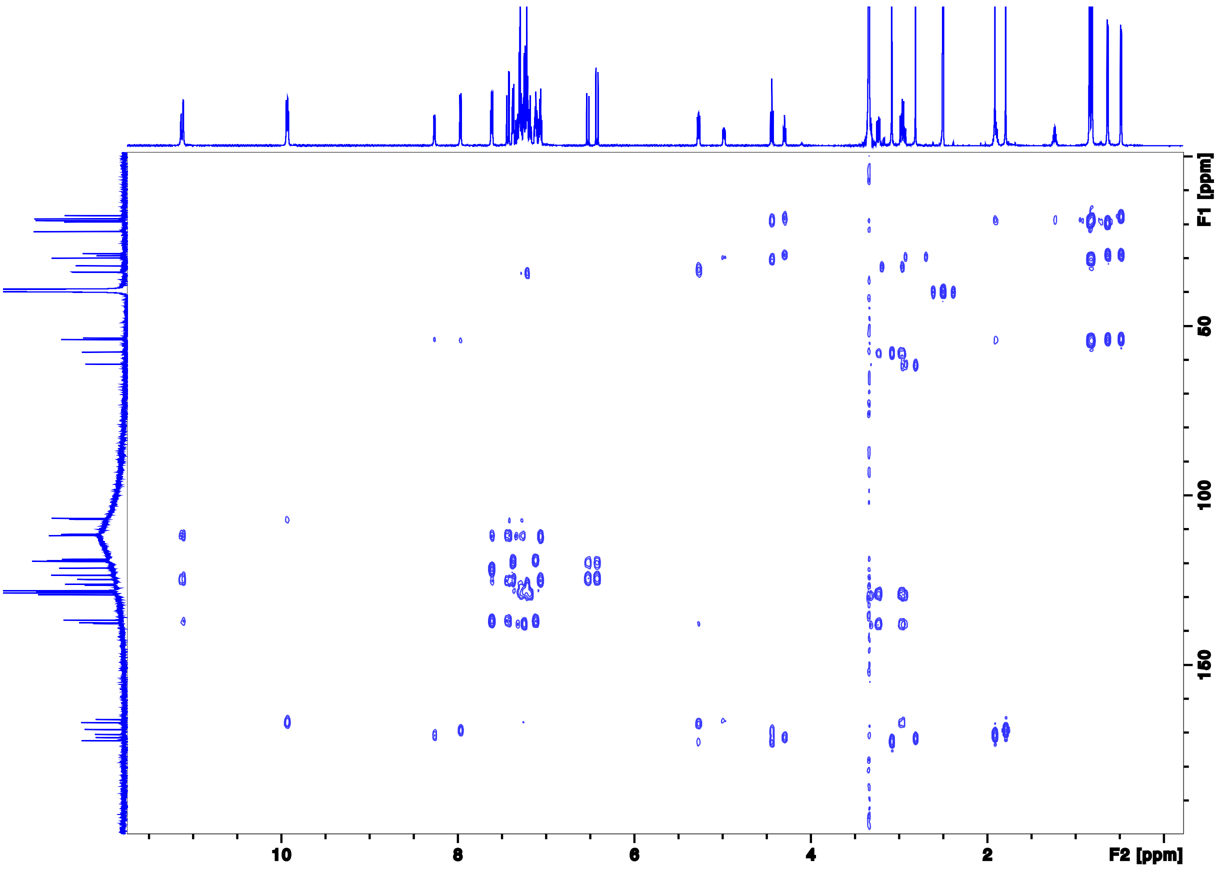

Figure S8. HMBC NMR (600 MHz, DMSO-d_6) spectrum of aspergillamide E (**7**).

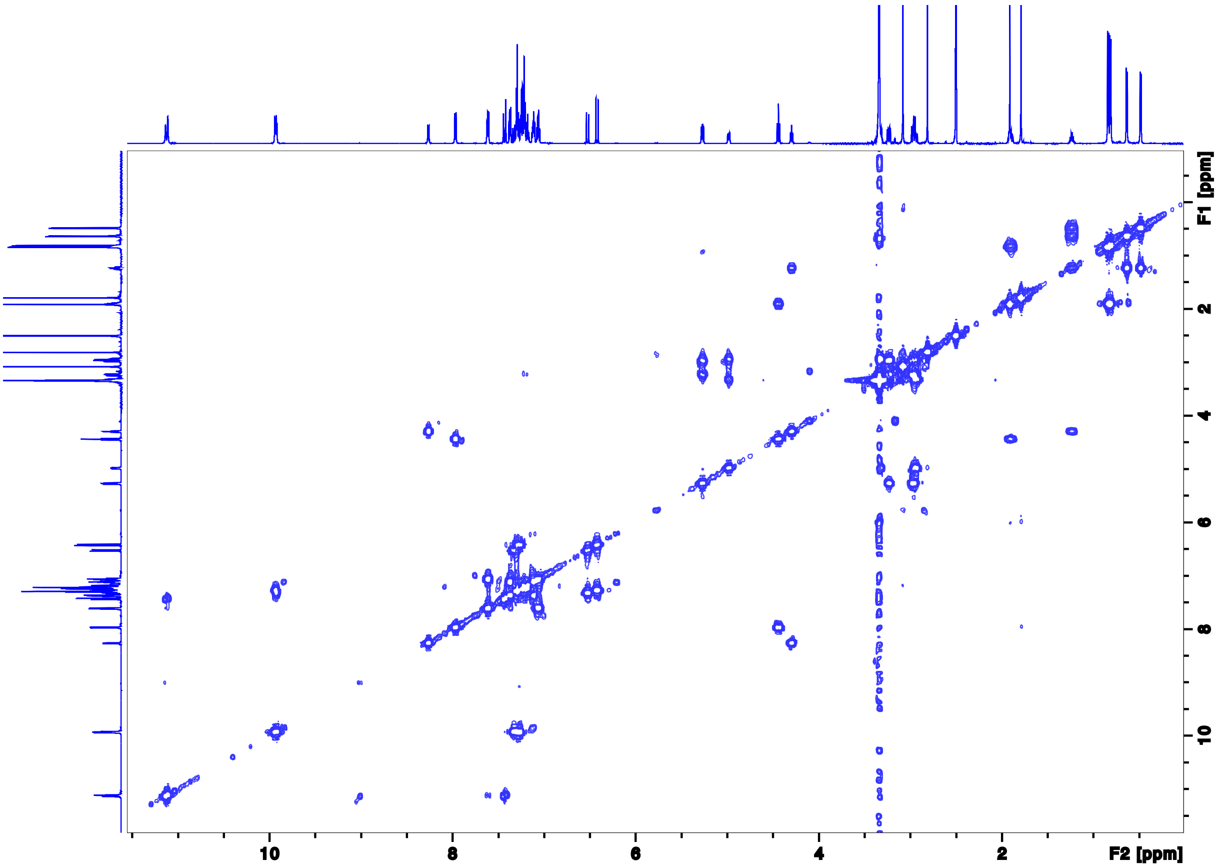

Figure S9. COSY NMR (600 MHz, DMSO-d_6) spectrum of aspergillamide E (**7**).

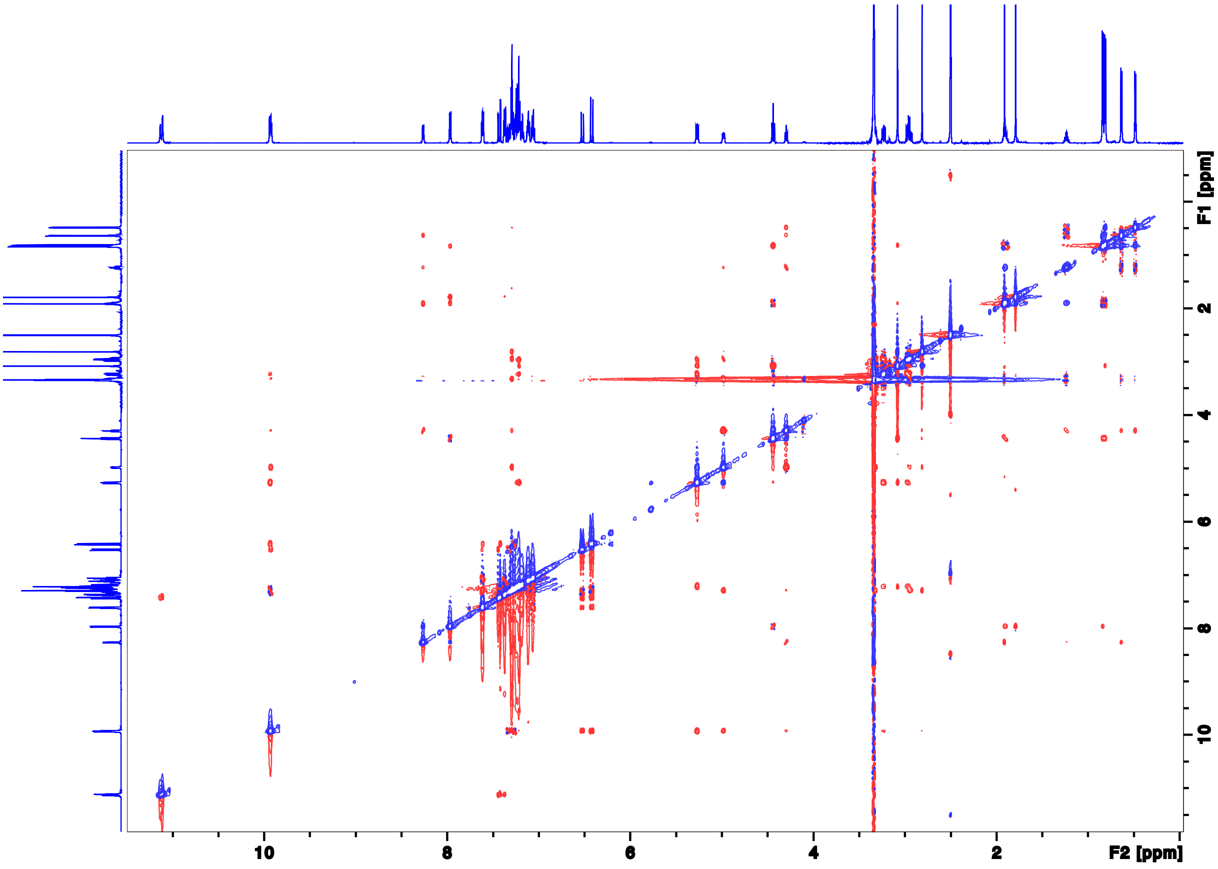

Figure S10. ROESY NMR (600 MHz, DMSO-d_6) spectrum of aspergillamide E (**7**).

Mass Spectrum Molecular Formula Report

Analysis Info

Analysis Name	D:\Data\Taizong\MRF324_com1a.d	Acquisition Date	1/13/2020 11:35:19 AM
Method	tune-med_AP.m	Operator	a.salim
Sample Name	MRF324_com1a	Instrument / Ser#	micrOTOF 213750.00232
Comment			

Acquisition Parameter

Source Type	ESI	Ion Polarity	Positive	Set Nebulizer	0.8 Bar
Focus	Not active			Set Dry Heater	180 °C
Scan Begin	100 m/z	Set Capillary	4500 V	Set Dry Gas	5.0 l/min
Scan End	1000 m/z	Set End Plate Offset	-500 V	Set Divert Valve	Source

Generate Molecular Formula Parameter

Formula, min.		
Formula, max.		
Measured m/z	Tolerance	Charge
Check Valence	Minimum	Maximum
Nirogen Rule	Electron Configuration	
Filter H/C Ratio	Minimum	Maximum
Estimate Carbon		

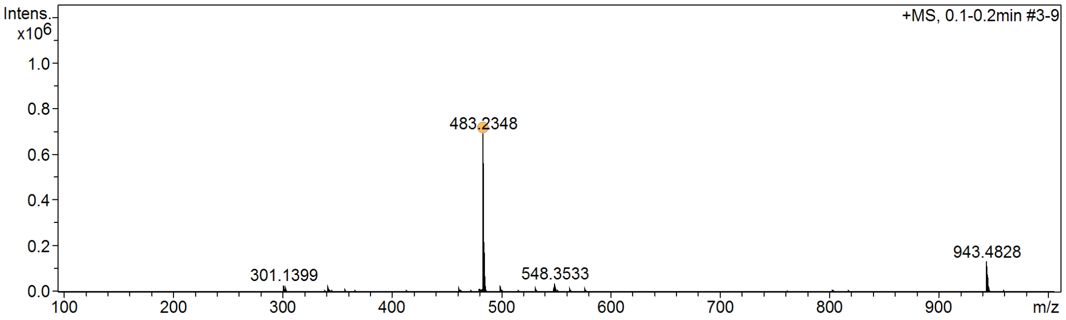

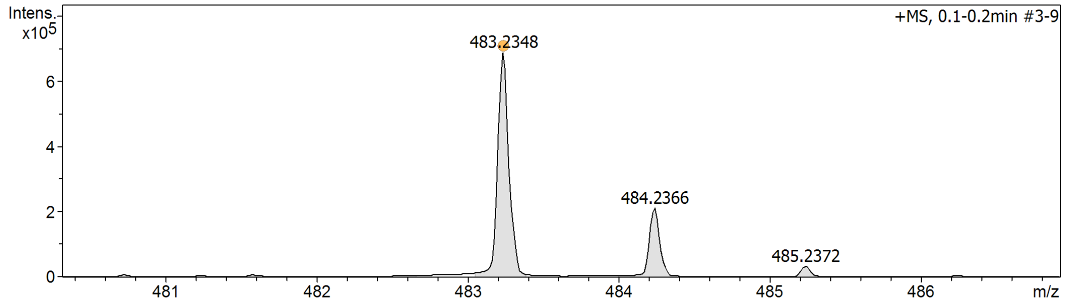

Meas. m/z	#	Ion Formula	m/z	err [ppm]	mSigma	# Sigma	Score	rdb	e⁻ Conf	N-Rule
483.2348	1	C27H32N4NaO3	483.2367	3.8	3.7	1	49.49	13.5	even	ok
	2	C23H28N10NaO	483.2340	1.7	9.2	2	86.58	14.5	even	ok
	3	C26H36NaO7	483.2353	1.1	9.8	3	100.00	8.5	even	ok
	4	C22H32N6NaO5	483.2326	4.5	20.6	4	28.38	9.5	even	ok
	5	C21H36N2NaO9	483.2313	-7.2	32.2	5	6.20	4.5	even	ok

Bruker Compass DataAnalysis 4.1 printed: 1/13/2020 11:43:52 AM Page 1 of 1

Figure S11. HRMS spectrum and measurement for aspergillamide E (**7**).

4.2 Aspergillamide F (8a-8b)

Table S3. 1D and 2D NMR (600 MHz, DMSO-d_6) data for aspergillamide F (**8a**)

	Pos.	δ_H, mult, (J in Hz)	δ_C	COSY	ROESY	HMBC
enamino-Trp	2	7.53, d (2.3)	123.6	1-NH	9-NH, 1-NH	7a, 3, 4
	3		109.5			
	3a		126.5			
	4	7.57, d (7.9)	118.3	5	9	7a, 3, 6
	5	7.02, dd (7.9, 7.3)	119.1	4		3a, 7
	6	7.12, dd (7.9, 7.3)	121.6	7		7a, 4
	7	7.38, d (7.9)	111.5	6		3a, 5
	7a		135.6			
	8	5.95, d (9.5)	103.3	9	5, 10	2, 3a
	9	6.64, dd (10.2, 9.5)	118.1	8, 9-NH	9	3, 8, Phe-1
	1-NH	11.29, br s		2	2, 7	7a, 2, 3, 3a
	9-NH	9.01, d (10.2)		9	2, Phe-2	Phe-1
N-Me-L-Phe	1		168.4			
	2	5.53, dd (9.9, 6.0)	56.8	3a, 3b	3a, 3b, 5, 9-NH	1, 3, 4, N-Me, Val-1
	3	a. 3.23, dd (15.0, 6.0)	33.5	2, 3b	2, 5	2, 4, 5
		b. 3.00, dd (15.0, 9.9)		2, 3a	2, 5	2, 4, 5
	4		137.4			
	5	7.22, dd (7.1, 7.1)	128.8		2, 3a, 3b	3, 7
	6	7.23, d (7.1)	128.1			4, 5
	7	7.17, (7.1)	126.3			5
	N-Me	3.03, s	31.6		2, Val-3, Val-4	2, Val-1
L-Val	1		172.8			
	2	4.46, dd (8.9, 8.9)	53.6	3, NH	4, 5	1, 3, 4, 5, N-acetyl
	3	1.89, dqq (8.9, 6.7, 6.7)	30.0	2, 4, 5	NH	2, 4, 5
	4	0.72, d (6.7)	18.9	3	2	2, 3, 5
	5	0.77, d (6.7)	18.2	3	2, NH	2, 3, 4
	NH	7.9, d (8.9)		2	3, 5, N-acetyl	N-acetyl
	NHAc		168.8			
		1.73, s	22.1		NH	

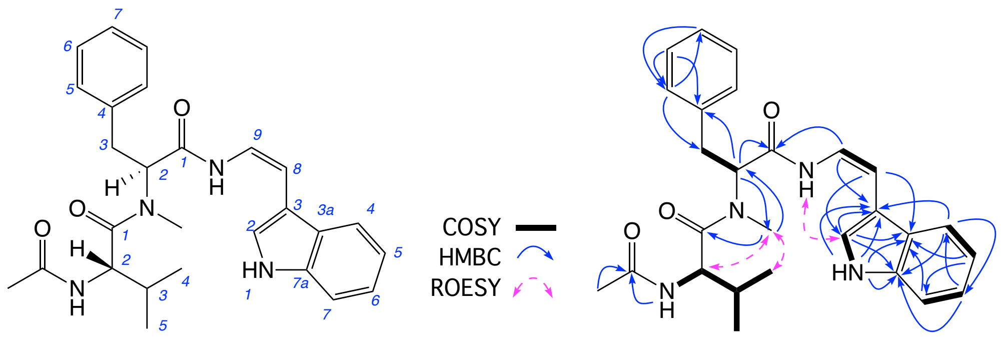

Table S4. 1D and 2D NMR (600 MHz, DMSO-d_6) data for aspergillamide F (**8b**)

	Pos.	δ_H, mult, (J in Hz)	δ_C	COSY	ROESY	HMBC
enamino-Trp	2	7.71, d (2.3)	124.4	1-NH	1-NH, 9-NH	7a, 3, 3a
	3		109.2			
	3a		126.6			
	4	7.57[A]	118.3	5	8	7a, 3, 6
	5	7.03[A]	119.1	4		3a, 7
	6	7.12[A]	121.5	7		7a, 4
	7	7.37, d (8.3)	111.4	6		3a, 5
	7a		135.6			
	8	5.99, d (9.5)	104.2	9	4, 9	2, 3a
	9	6.61, dd (9.8, 9.5)	117.9	8, 9-NH	8	3, 8, Phe-1
	1-NH	11.30, br s		2	2, 7	7a, 2, 3, 3a
	9-NH	9.23, d (9.8)		9	2, Phe-2	Phe-1
N-Me-L-Phe	1		167.6			
	2	5.30, dd (8.8, 6.0)	60.6	3a, 3b	9-NH, 3b, 5, Val-2	1, 3, 4, N-Me, Val-1
	3	a. 3.37[B]	35.4	3b		4, 5
		b. 2.96, dd (14.4, 8.8)		2, 3a	2, 5	2, 4, 5
	4		137.5			
	5	7.31, d (7.0)	129.3		2, 3a, 3b	3, 7
	6	7.29, dd (7.0, 7.0)	128.3			4, 5
	7	7.22[A]	126.5			5
	N-Me	2.89, s	29.7		2	2, Val-1
L-Val	1		171.8			
	2	4.26, dd (8.1, 8.1)	54.0	3, NH	Phe-2, 3, 4, 5	1, 3, 4, 5, N-acetyl
	3	1.56, dqq (8.1, 6.8, 6.8)	29.2	2, 4, 5	2, NH	2, 4, 5
	4	0.48, d (6.8)	19.3	3	2	2, 3, 5
	5	0.69, d (6.8)	18.0	3	2, 2-NH	2, 3, 4
	NH	8.24, d (8.1)		2	3, 5, N-acetyl	N-acetyl
	NHAc		170.2			
		1.66, s	21.7		NH	

[A] *E/Z* amide rotamer resonances overlapping; [B] Resonance obscured by residual H_2O and detected by HSQC

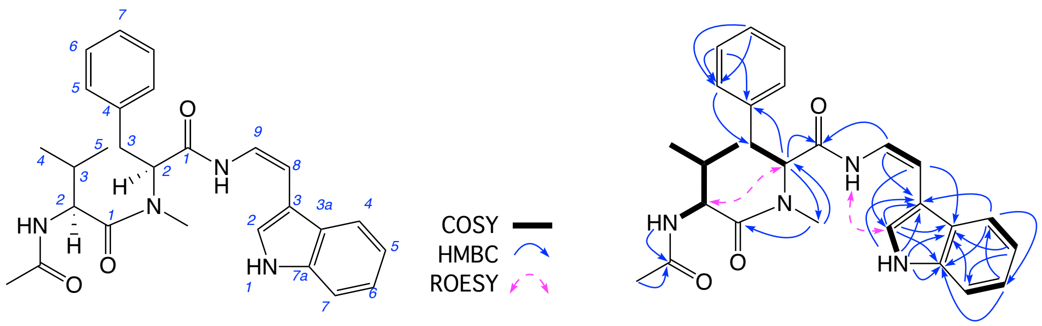

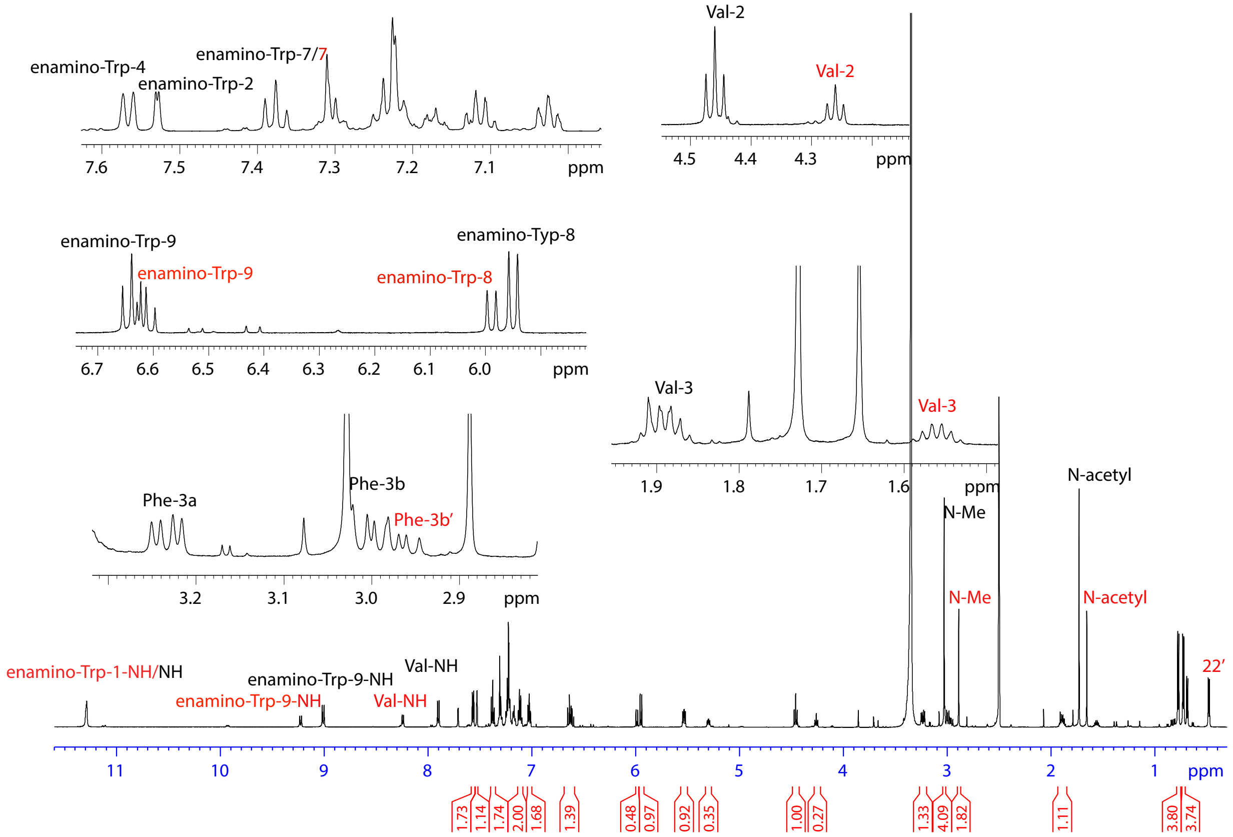

Figure S12. ^{1}H NMR (600 MHz, DMSO-d_6) spectrum of aspergillamide F (**8a**, labelled in black; **8b**, labelled in red).

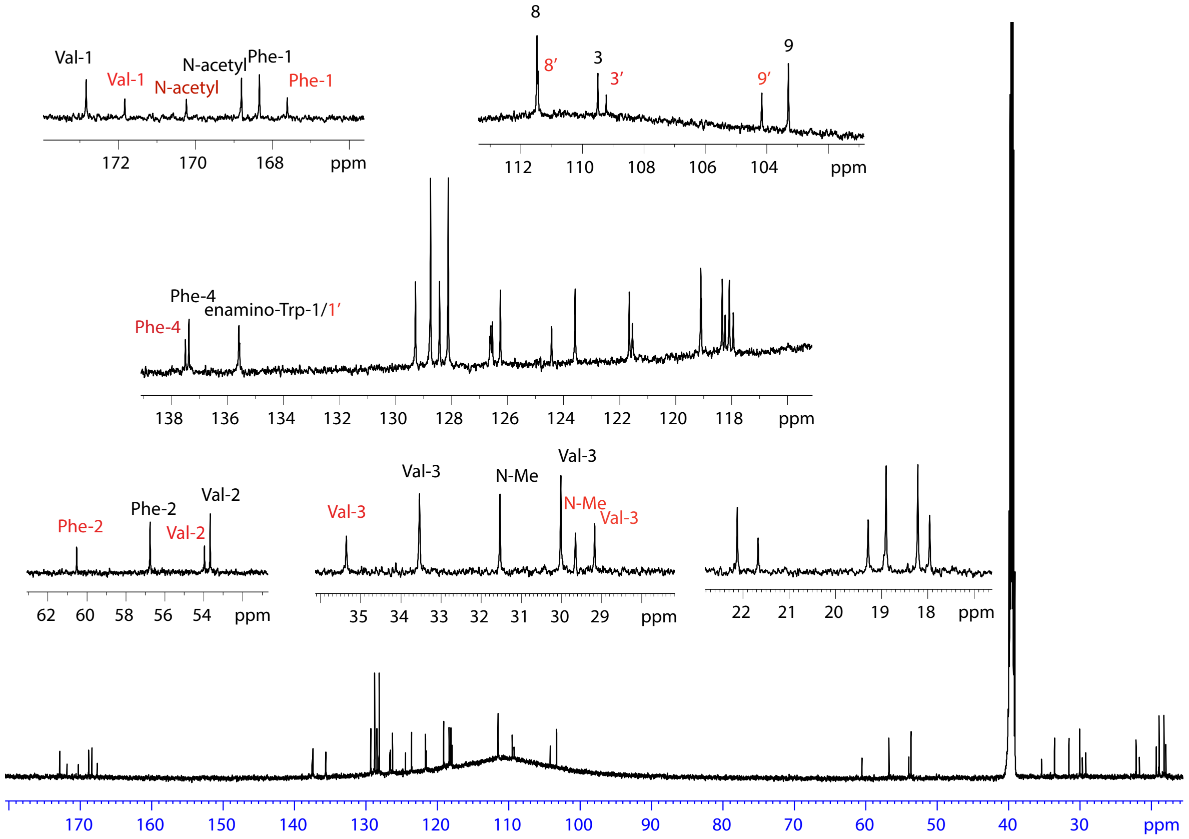

Figure S13. ^{13}C NMR (150 MHz, DMSO-d_6) spectrum of aspergillamide F (**8a**, labelled in black; **8b**, labelled in red).

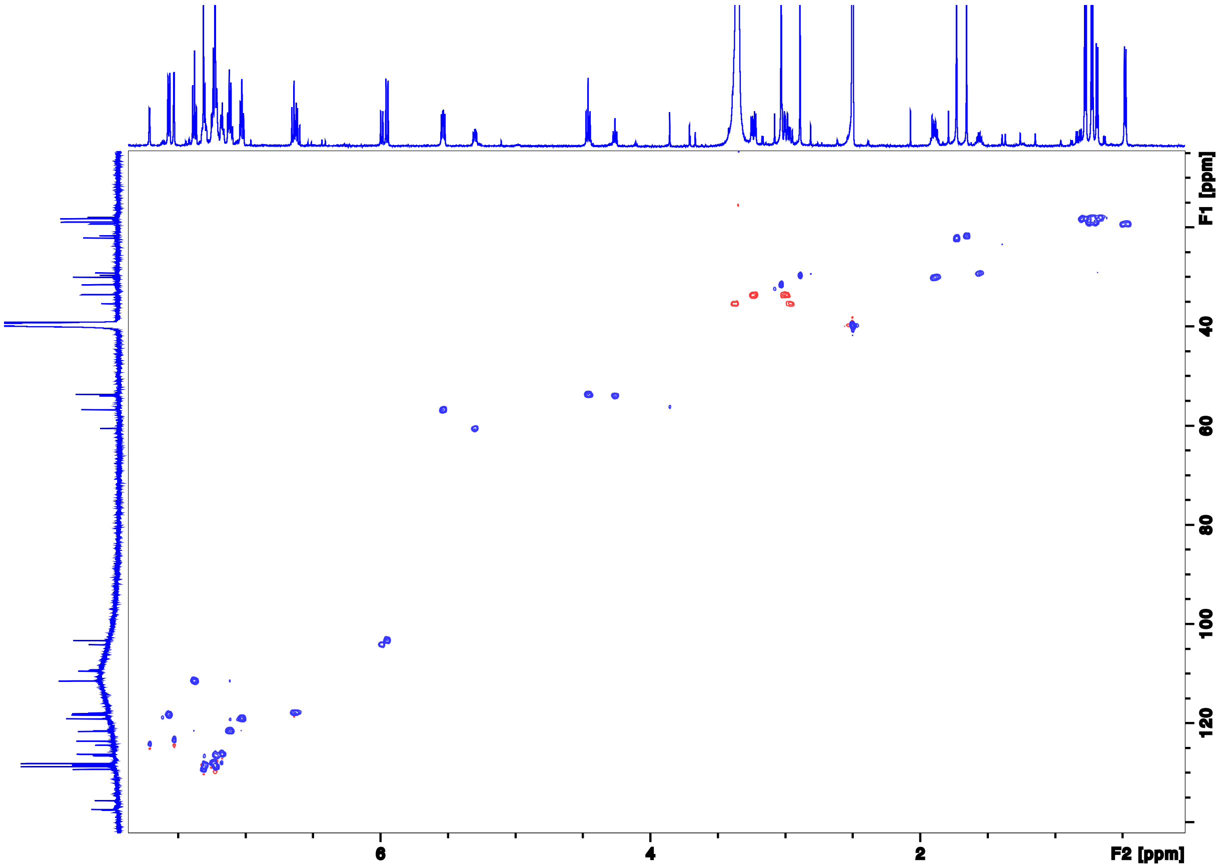

Figure S14. HSQC NMR (600 MHz, DMSO-d_6) spectrum of aspergillamide F (**8**).

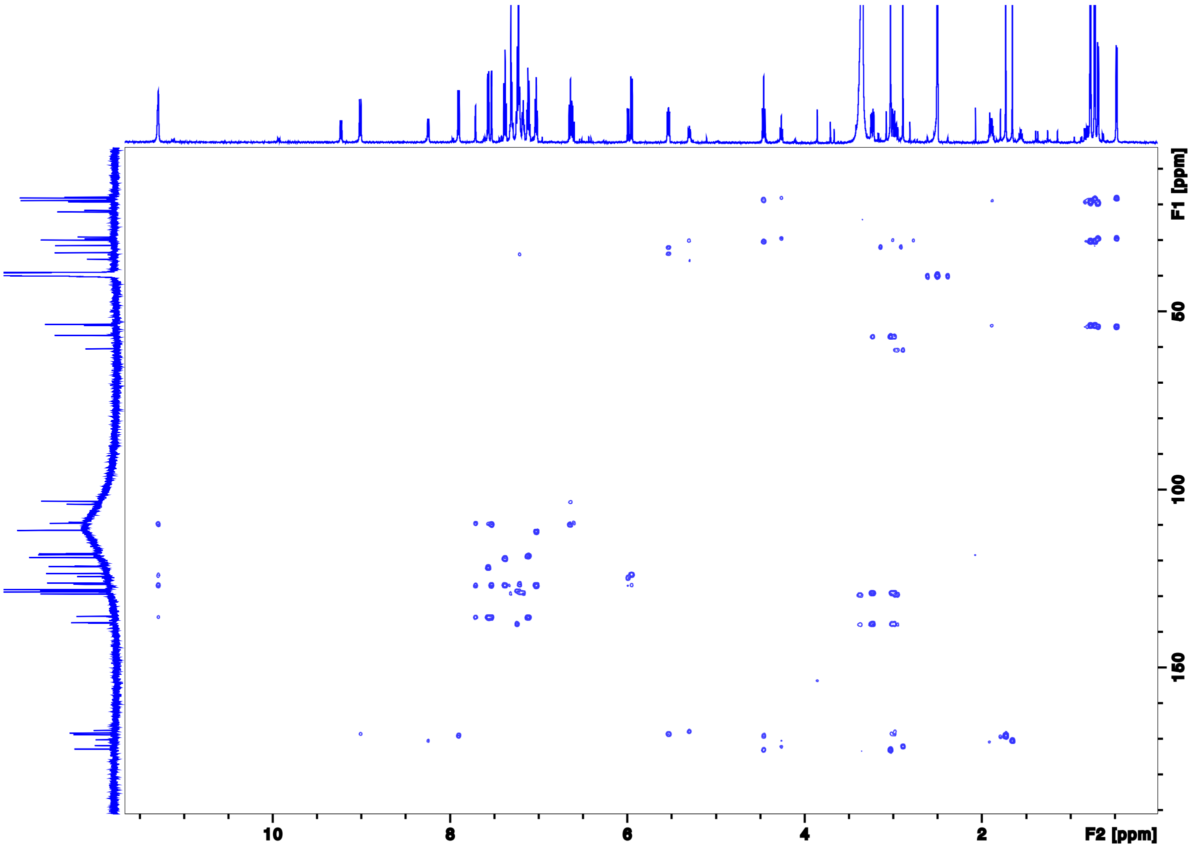

Figure S15. HMBC NMR (600 MHz, DMSO-d_6) spectrum of aspergillamide F (**8**).

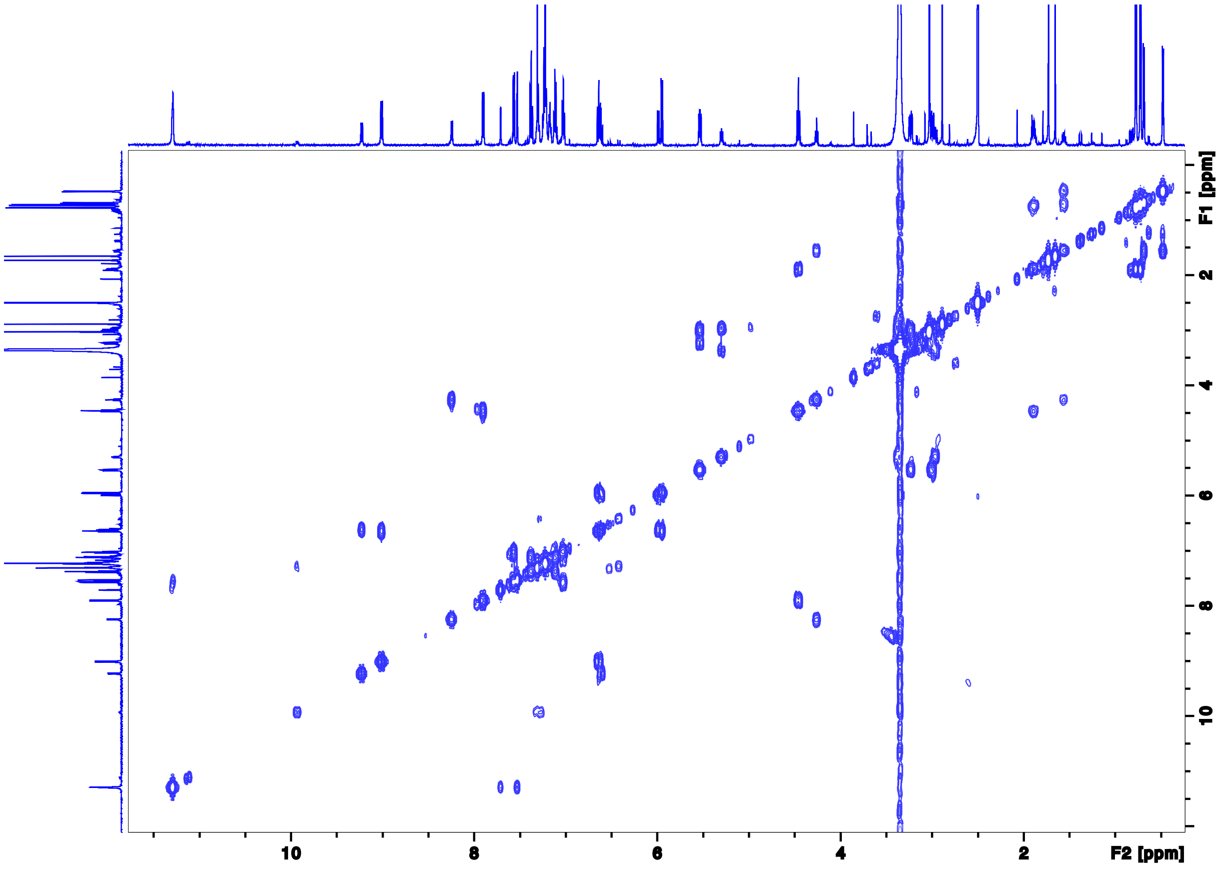

Figure S16. COSY NMR (600 MHz, DMSO-d_6) spectrum of aspergillamide F (**8**).

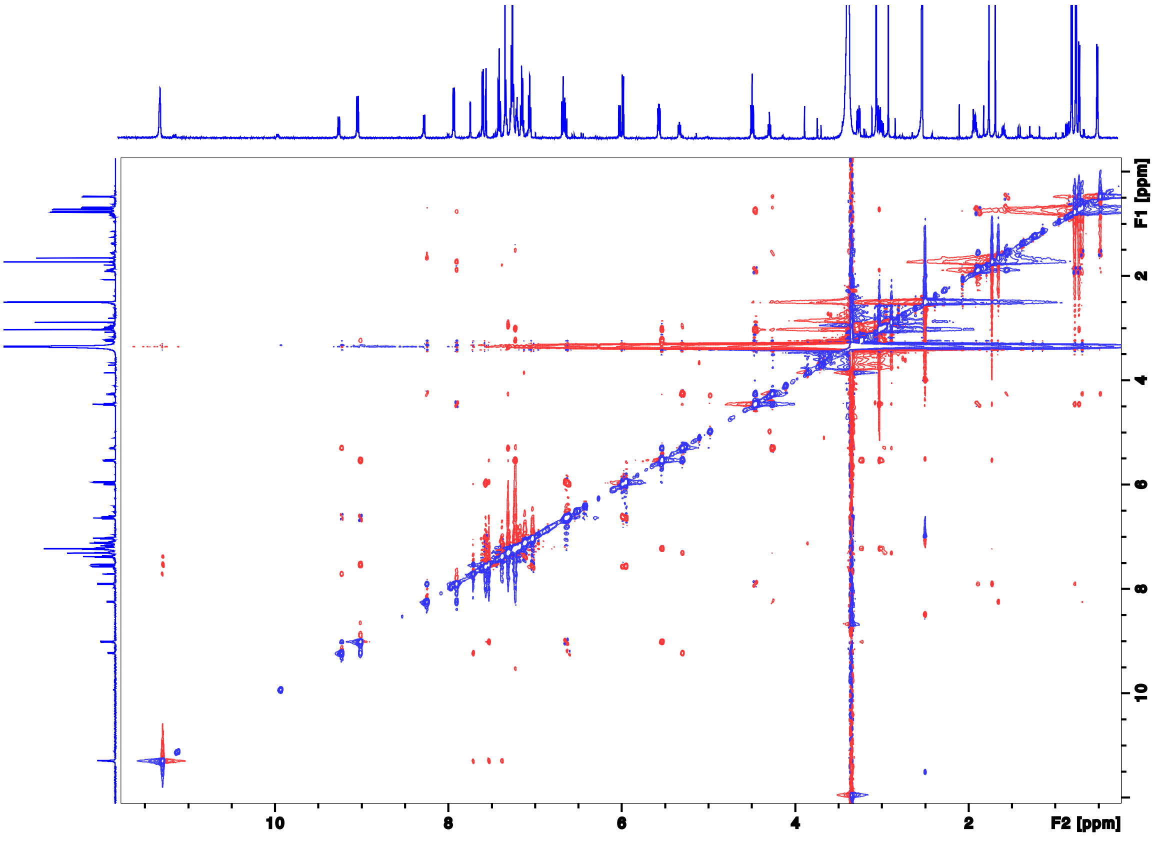

Figure S17. HMBC NMR (600 MHz, DMSO-d_6) spectrum of aspergillamide F (**8**).

Mass Spectrum Molecular Formula Report

Analysis Info

Analysis Name	D:\Data\Taizong\CMB_MRF324_com1c_2.d	Acquisition Date	1/16/2020 2:51:06 PM
Method	tune-med_AP.m	Operator	a.salim
Sample Name	CMB_MRF324_com1c_2	Instrument / Ser#	micrOTOF 213750.00232
Comment			

Acquisition Parameter

Source Type	ESI	Ion Polarity	Positive	Set Nebulizer	0.8 Bar
Focus	Not active			Set Dry Heater	180 °C
Scan Begin	100 m/z	Set Capillary	4500 V	Set Dry Gas	5.0 l/min
Scan End	1000 m/z	Set End Plate Offset	-500 V	Set Divert Valve	Source

Generate Molecular Formula Parameter

Formula, min.		
Formula, max.		
Measured m/z	Tolerance	Charge
Check Valence	Minimum	Maximum
Nirogen Rule	Electron Configuration	
Filter H/C Ratio	Minimum	Maximum
Estimate Carbon		

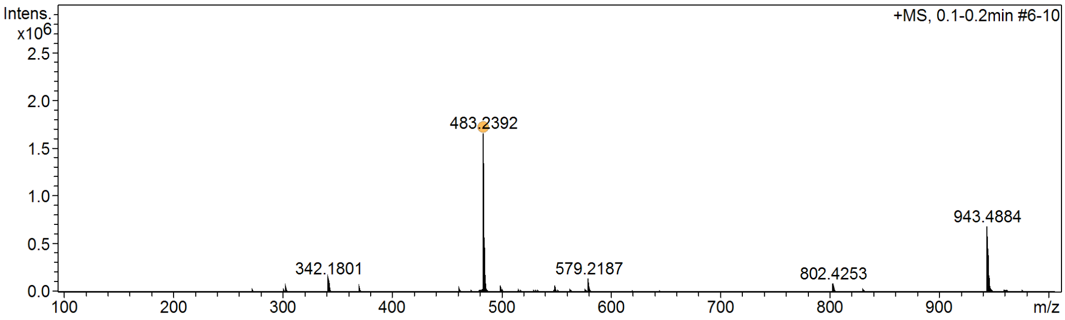

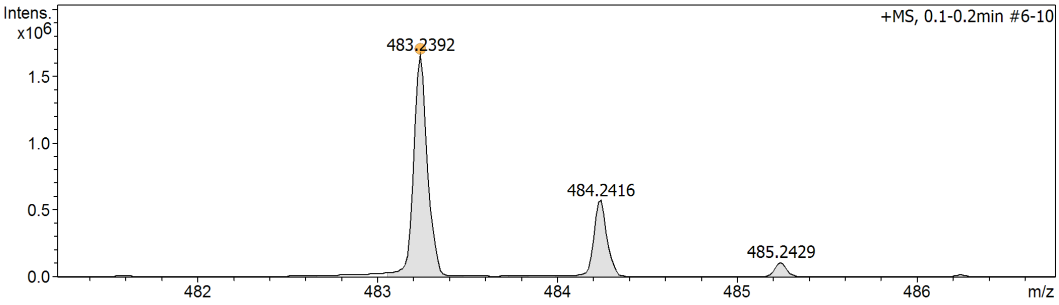

Meas. m/z	#	Ion Formula	m/z	err [ppm]	mSigma	# Sigma	Score	rdb	e⁻ Conf	N-Rule
483.2392	1	C32H32N2NaO	483.2407	3.2	6.7	1	100.00	17.5	even	ok
	2	C27H32N4NaO3	483.2367	5.2	17.4	2	39.13	13.5	even	ok
	3	C26H36NaO7	483.2353	-7.9	28.6	3	7.98	8.5	even	ok

Bruker Compass DataAnalysis 4.1 printed: 1/16/2020 2:56:31 PM Page 1 of 1

Figure S18. HRMS spectrum and measurement for aspergillamide F (**8**).

4.3 Asterriquinone SU5228 (9)

Table S5. 1D and 2D NMR (600 MHz, DMSO-d_6) data for asterriquinone SU5228 (**9**)

Pos.	δ_H, mult, (J in Hz)	δ_C	COSY	ROESY	HMBC
1		n.d.			
2		n.d			
3		n.d.			
4		n.d.			
5		n.d.			
6		n.d.			
2′		137.9			
3′		101.4			
4′	7.16, d (7.9)	119.4	5′		6′, 8′
5′	6.91, dd (7.9)	118.4	4′, 6′		7′, 9′
6′	7.01, dd (7.9)	120.1	5′, 7′		4′, 8′
7′	7.32, d (7.9)	110.6	6′, 1′-NH	1′-NH	5′, 9′
8′		135.4			
9′		128.0			
10′	3.33[A]	26.4	11′		2′, 3′, 11′, 12′
11′	5.3, t (6.8)	121.2	10′	13′, 14′	10′, 12′, 13′
12′		132.1			
13′	1.64, s	17.6		11′	11′, 12′, 14′
14′	1.67, s	25.5		11′	11′, 12′, 13′
2″	7.50, d (2.5)	127.2		1″-NH	3, 8″, 9″
3″		104.6			
4″	7.42, d (7.9)	121.5	5″		6″, 8″
5″	6.99, dd (7.9, 7.9)	118.5	4″, 6″		7″, 9″
6″	7.10, dd (7.9, 7.9)	120.8	5″, 7″		4″, 8″
7″	7.41, d (7.9)	111.4	6″	1″-NH	5″, 9″
8″		135.7			
9″		126.5			
1′-NH	10.98, s			7′, 10′, 11′	3′, 9′
1″-NH	11.36, s			2″, 7″	

[A] Resonance obscured by H_2O but detected by HSQC; n.d. Not detected.

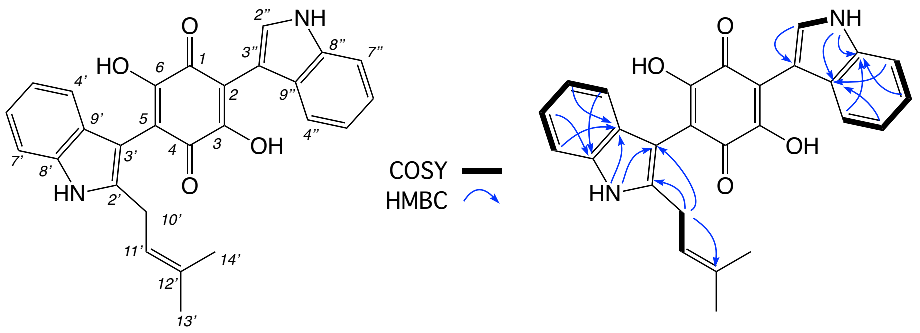

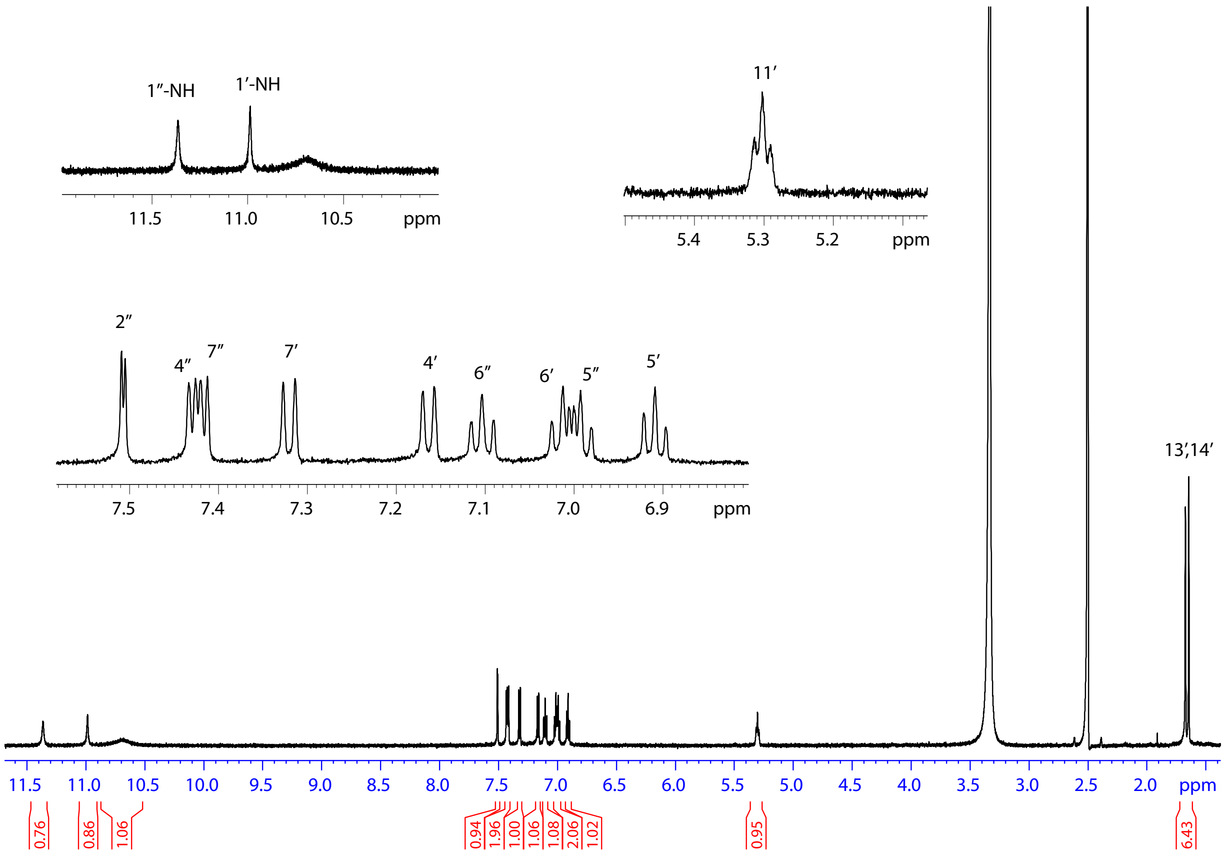

Figure S19. ^{1}H NMR (600 MHz, DMSO-d_6) spectrum of asterriquinone SU5228 (**9**).

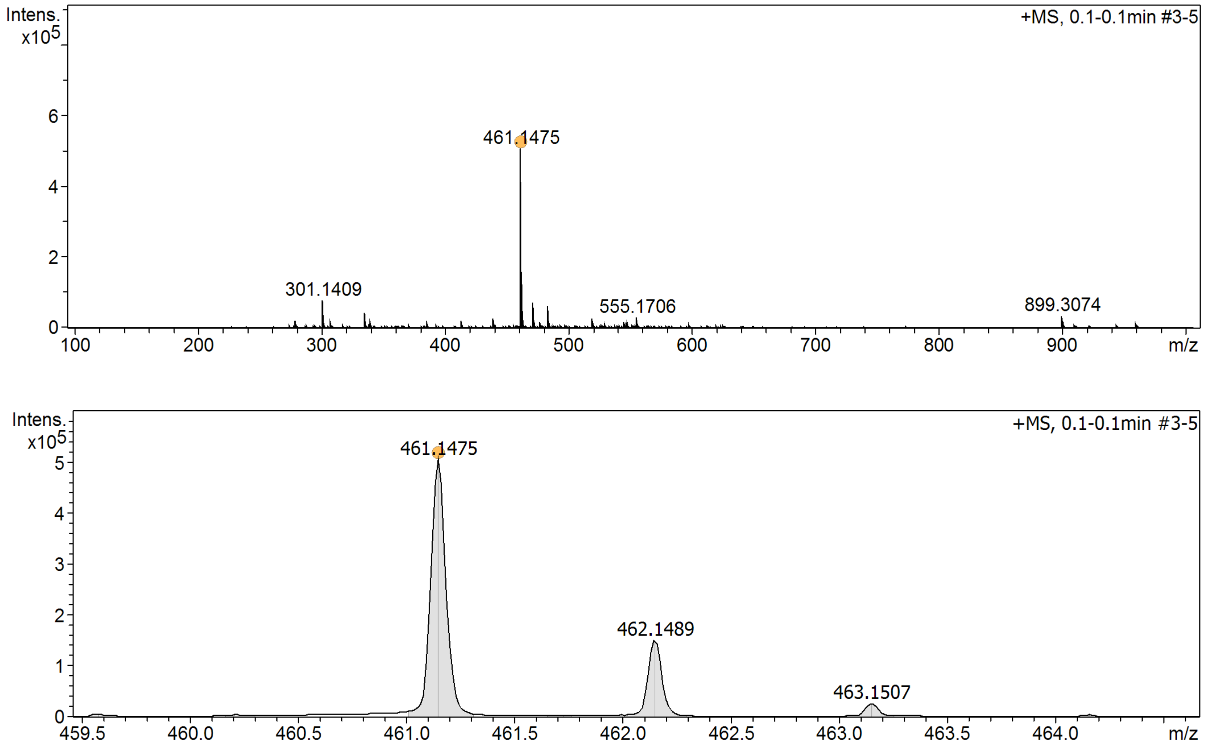

Meas. m/z	#	Ion Formula	m/z	err [ppm]	mSigma	# Sigma	Score	rdb	e⁻ Conf	N-Rule
461.1475	1	C27H22N2NaO4	461.1472	0.6	3.6	1	100.00	17.5	even	ok

Figure S20. HRMS spectrum and measurement for asterriquinone SU5228 (**9**).

4.4 Asterriquinone CT5 (10)

Table S6. 1D and 2D NMR (600 MHz) data for asterriquinone CT5 (**10**)

Pos.	δ_H, mult, (*J* in Hz)*	δ_C*	COSY*	ROESY*	HMBC*	δ_H, mult, (*J* in Hz)**	δ_C, **
1		n.d.					
2		n.d					
3		n.d.					
4		n.d.					
5		n.d.					
6		n.d.					
2′		137.8					138.4
3′		101.5					100.5
4′	7.20, d (8.0)[B]	119.5	5′		6′, 8′	7.33, d (7.7)	119.3
5′	6.92, dd (8.0, 8.0)	118.4	4′, 6′		7′, 9′	7.12, dd (7.7, 7.7)	120.1
6′	7.02, dd (8.0, 8.0)	120.1	5′, 7′		4′, 8′	7.17, dd (7.7, 7.7)	121.7
7′	7.33, d (8.0)	110.7	6′	1′-NH	5′, 9′	7.35, d (7.7)	110.9
8′		135.4					135.3
9′		128.0					127.9
10′	3.34[A]	26.4	11′	1′-NH	2′, 3′, 11′, 12′	3.47, dd (13.3, 7.2)	26.9
11′	5.30, t (7.3)	121.2	10′	1′-NH	10′, 13′, 14′	5.41, br. s	119.7
12′		132.2					135.9
13′	1.65, s[C]	17.6			11′, 12′, 14′	1.77, br. s	18.1
14′	1.68, s[D]	25.6			11′, 12′, 13′	1.82, br. s	25.9
2″		137.8					
3″		101.5					
4″	7.13, d (8.0)[B]	119.2	5″		6″, 8″		
5″	6.92, dd (8.0, 8.0)	118.4	4″, 6″		7″, 9″		
6″	7.02, dd (8.0, 8.0)	120.1	5″, 7″		4″, 8″		
7″	7.33, d (8.0)	110.7	6″	1″-NH	5″, 9″		
8″		135.4					
9″		128.0					
10″	3.34[A]	26.4	11″	1″-NH	2″, 3″, 11″, 12″		
11″	5.30, t (7.3)	121.2	10″	1″-NH	10″, 13″, 14″		
12″		132.2					
13″	1.63, s[C]	17.6			11″, 12″, 14″		
14″	1.67, s[D]	25.6			11″, 12″, 13″		
1′-NH	11.02, s			7′, 10′, 11′	2′, 3′, 8′, 9′		
1″-NH	11.02, s			7″, 10″,	2″, 3″, 8″, 9″		

* Data acquired in DMSO-d_6; ** Data acquired in $CDCl_3$; [A]Resonance obscured by H_2O but detected by HSQC; [B-D] Assignments with same letter are interchangeable;

Note: The asymmetry of the NMR data of **10** in DMSO-d_6 and methanol-d_4 was proposed due to formation of atropisomers. The atropisomer issue was solved when acquiring NMR data of **10** in nonpolar $CDCl_3$.

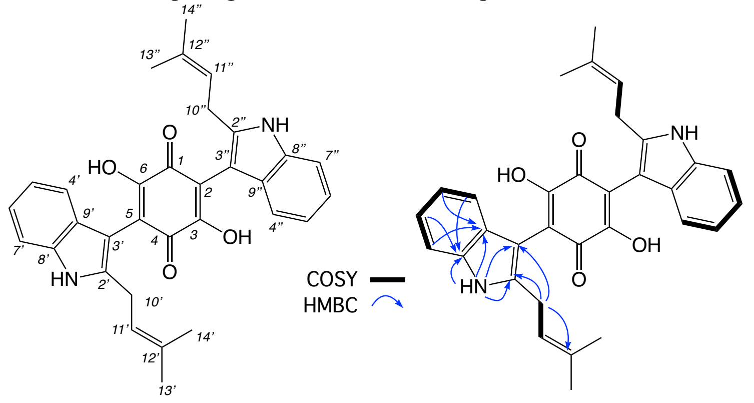

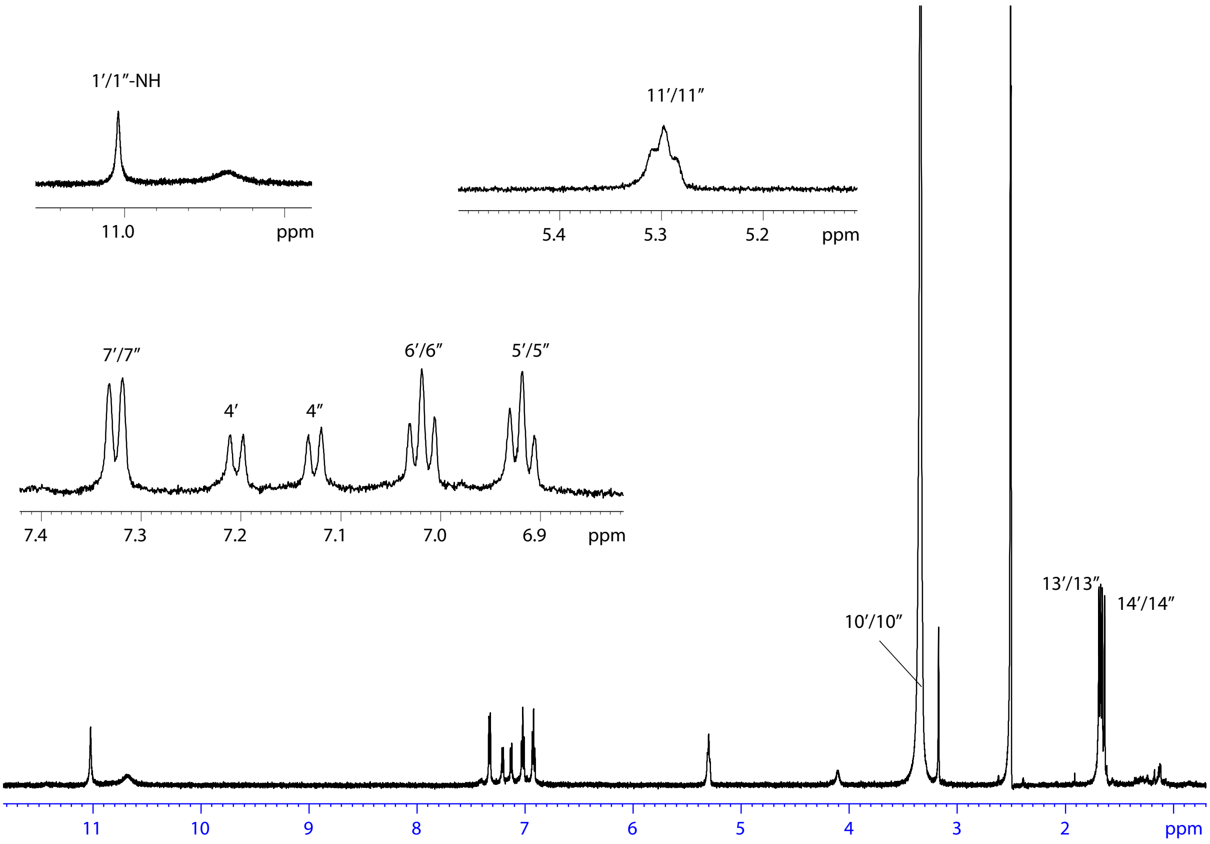

Figure S21. ^{1}H NMR (600 MHz, DMSO-d_6) spectrum of asterriquinone CT5 (**10**).

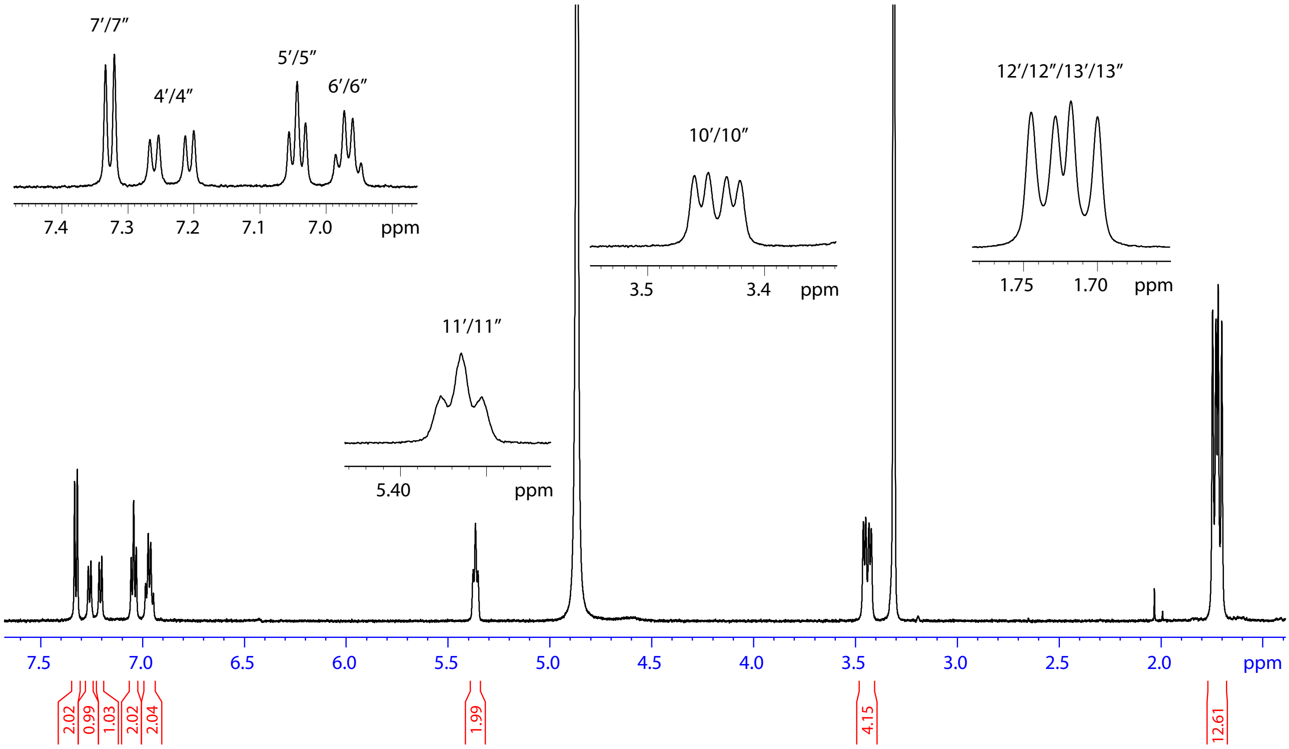

Figure S22. ^{1}H NMR (600 MHz, methanol-d_4) spectrum of asterriquinone CT5 (**10**).

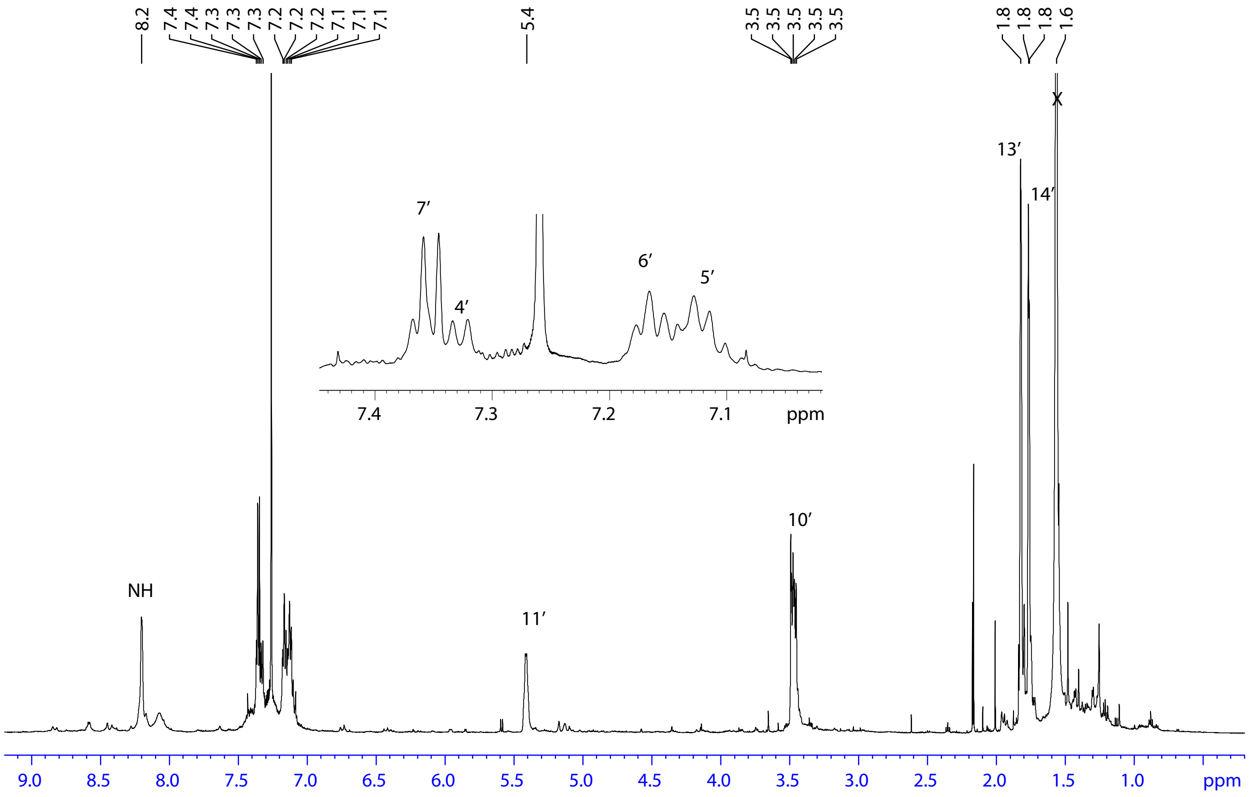

Figure S23. ^{1}H NMR (600 MHz, $CDCl_3$) spectrum of asterriquinone CT5 (**10**).

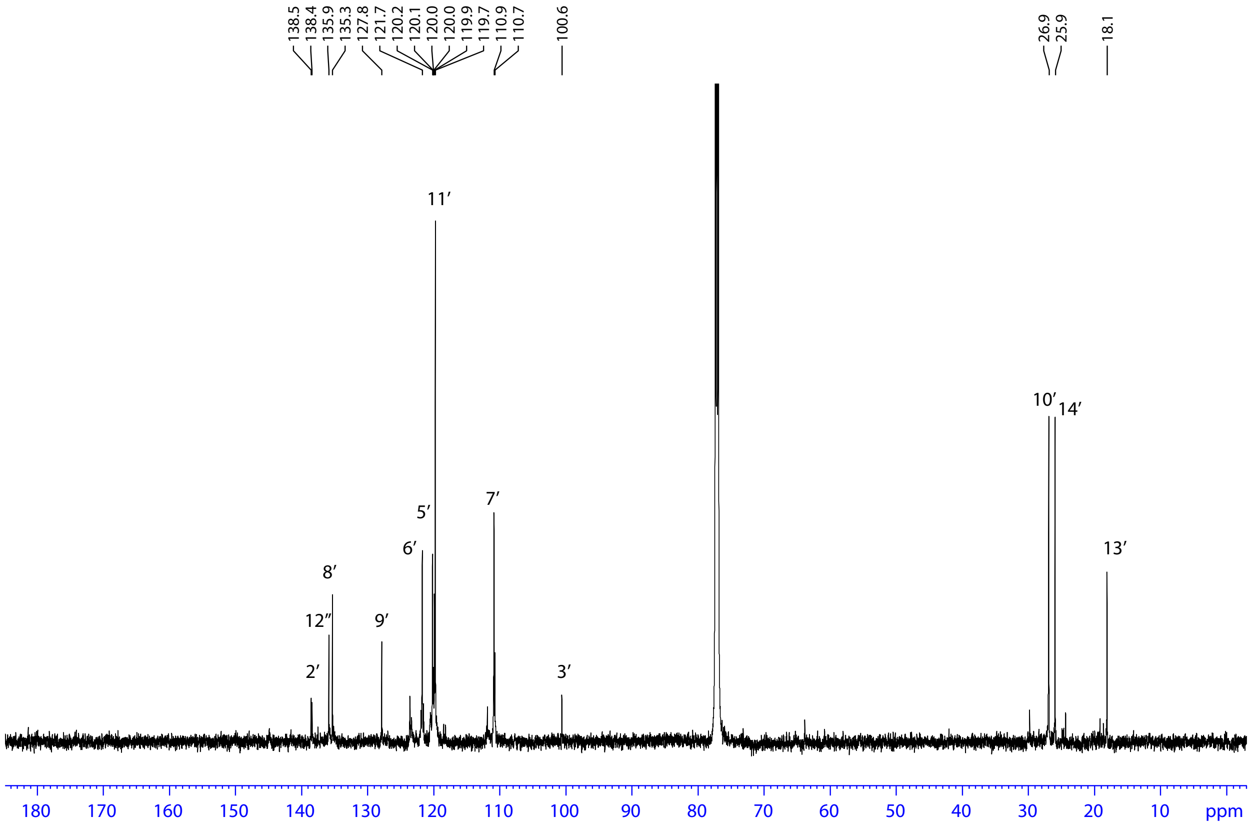

Figure S24. ^{13}C NMR (150 MHz, $CDCl_3$) spectrum of asterriquinone CT5 (**10**).

Mass Spectrum Molecular Formula Report

Analysis Info

Analysis Name	D:\Data\Taizong\CMB_MRF324_com4.d	Acquisition Date	1/5/2020 12:28:05 PM
Method	tune-med_AP.m	Operator	a.salim
Sample Name	CMB_MRF324_com4	Instrument / Ser#	micrOTOF 213750.00232
Comment			

Acquisition Parameter

Source Type	ESI	Ion Polarity	Positive	Set Nebulizer	0.8 Bar
Focus	Not active			Set Dry Heater	180 °C
Scan Begin	100 m/z	Set Capillary	4500 V	Set Dry Gas	5.0 l/min
Scan End	1000 m/z	Set End Plate Offset	-500 V	Set Divert Valve	Source

Generate Molecular Formula Parameter

Formula, min.		
Formula, max.		
Measured m/z	Tolerance	Charge
Check Valence	Minimum	Maximum
Nirogen Rule	Electron Configuration	
Filter H/C Ratio	Minimum	Maximum
Estimate Carbon		

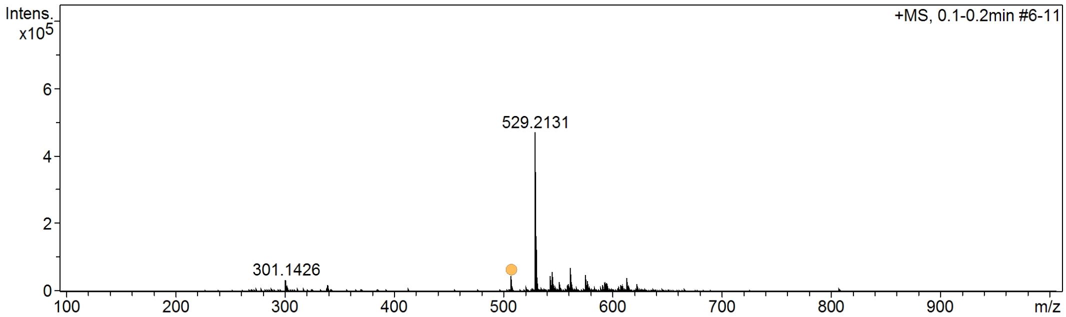

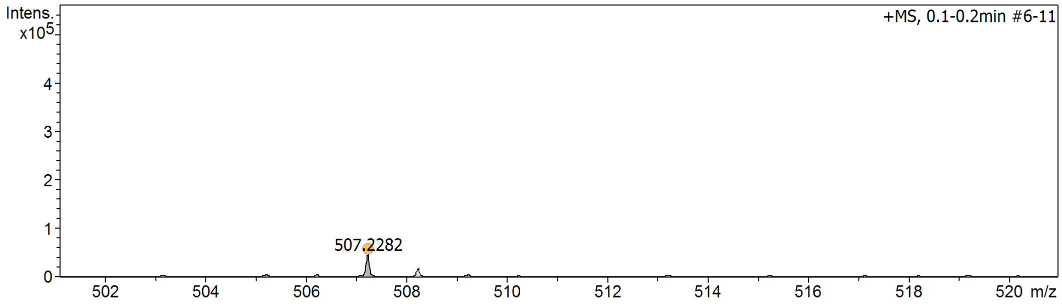

Meas. m/z	#	Ion Formula	m/z	err [ppm]	mSigma	# Sigma	Score	rdb	e⁻ Conf	N-Rule
507.2282	1	C32H31N2O4	507.2278	0.7	19.1	1	100.00	18.5	even	ok
	2	C33H27N6	507.2292	2.0	21.7	2	67.67	23.5	even	ok
	3	C28H27N8O2	507.2251	6.0	26.4	3	12.81	19.5	even	ok
	4	C37H31O2	507.2319	-7.3	26.9	4	6.39	22.5	even	ok

Bruker Compass DataAnalysis 4.1 printed: 1/5/2020 12:39:24 PM Page 1 of 1

Figure S25. HRMS spectrum and measurement for asterriquinone CT5 (**10**).

4.5 Aflaquinolone H (11)

Table S7. 1D and 2D NMR (600 MHz, $CDCl_3$) data for aflaquinolone H (**11**)

Pos.	δ_H, mult, (*J* in Hz)	δ_C	COSY	ROESY	HMBC
2		165.8			
3	3.70, br d (1.5)	84.3		3-OMe, 4-OH, 12/16	2, 4, 5, 11, 3-OMe
3-OMe	3.61, s	59.1		3, 4-OH	3
4		78.9			
5		111.1			
6		155.2			
7		122.4			
8	7.40, d (8.2)	127.3	9		6, 10, 11, 17
9	6.36, d (8.2)	107.0	8		5, 7
10		134.4			
11		129.2			
12	7.18, d (8.8)	128.0	13	3	4, 14, 16
13	6.82, d (8.8)	114.5	12		11, 14, 15
14		160.5			
14-OMe	3.76, s	55.4			14
15	6.82, d (8.8)	114.5	16		
16	7.18, d (8.8)	128.0	15	3	
17	6.79, d (16.6)	122.7	18		6, 8, 19
18	6.28, d (16.6)	135.9	17		7, 20, 22, 24
19		37.6			
20	a. 2.11, m	47.7	20b, 21		18, 19, 22, 25, 26
	b. 1.47, dd (13.4, 13.4)		20a, 21		
21	2.54, m	41.5	20a, 20b, 26		20, 22, 26
22		214.0			
23	a. 2.49, dd (14.2, 5.7)	38.7	23b, 24a, 24b		19, 22, 24
	b. 2.24, m		23a, 24a, 24b		
24	a. 2.15, m	38.5	23a, 23b, 24b		18, 25
	b. 1.73, td (13.4, 4.4)		23a, 24b, 24a		
25	1.10, s	30.6			18, 19, 20, 22
26	0.99, d (6.5)	14.6	21		20, 21, 22
1-NH	7.75, br s				2, 3, 5, 9, 10
4-OH	4.58, s			3, 3-OMe	3, 4, 11
6-OH	9.14, s				5, 6, 7

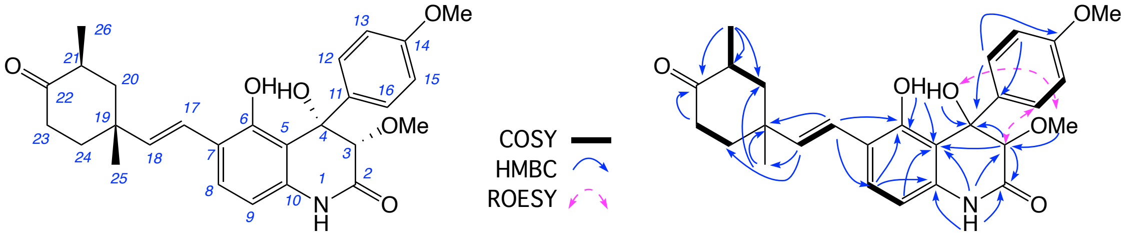

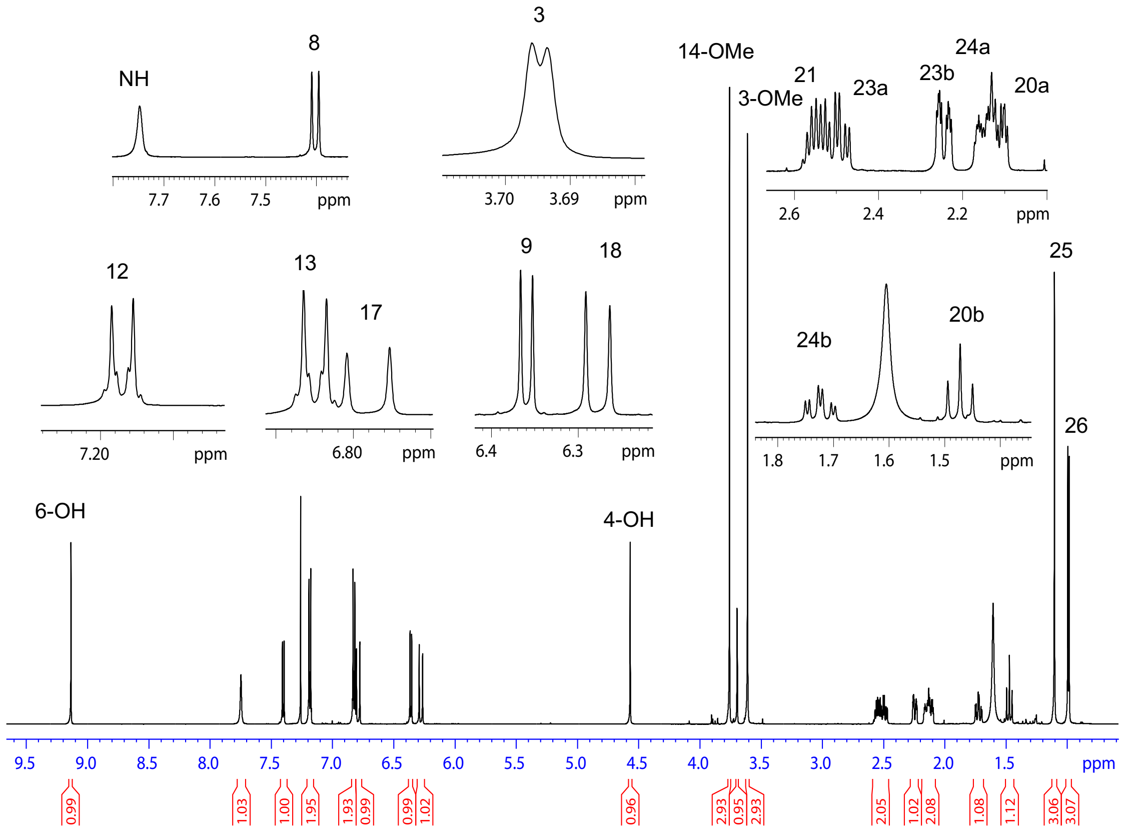

Figure S26. ^{1}H NMR (600 MHz, $CDCl_3$) spectrum for aflaquinolone H (**11**).

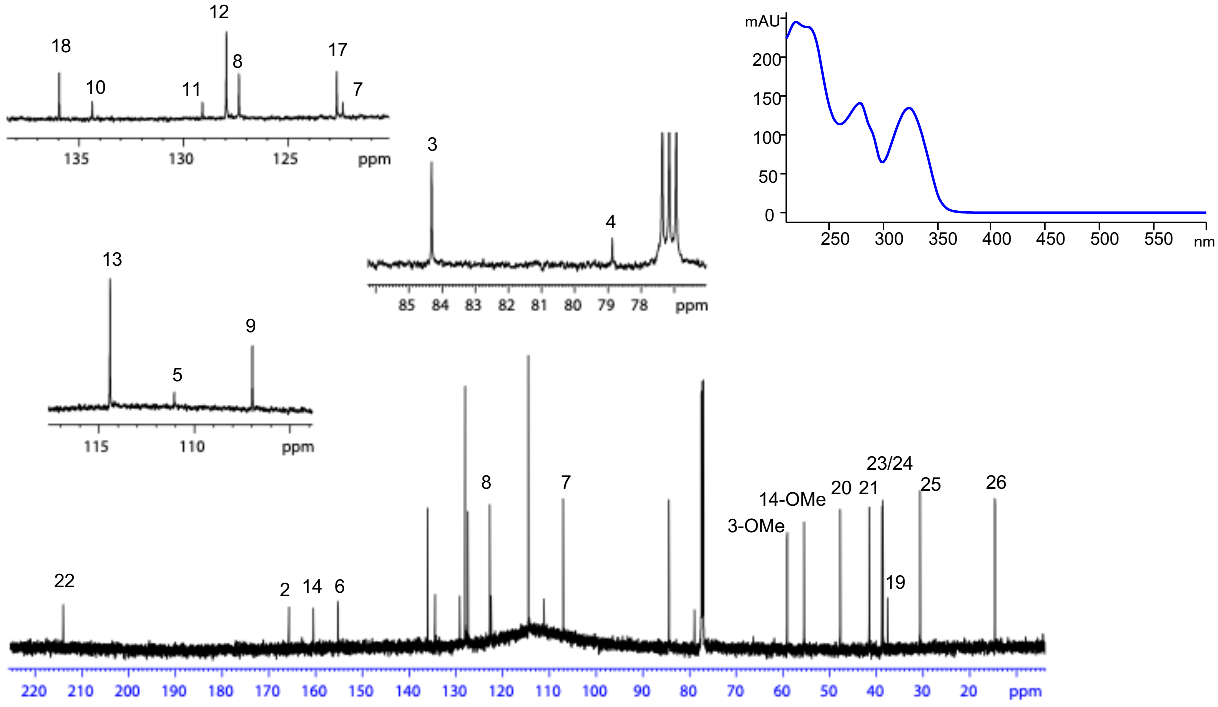

Figure S27. ^{13}C NMR (150 MHz, $CDCl_3$) and UV-vis (inset) spectra for aflaquinolone H (**11**).

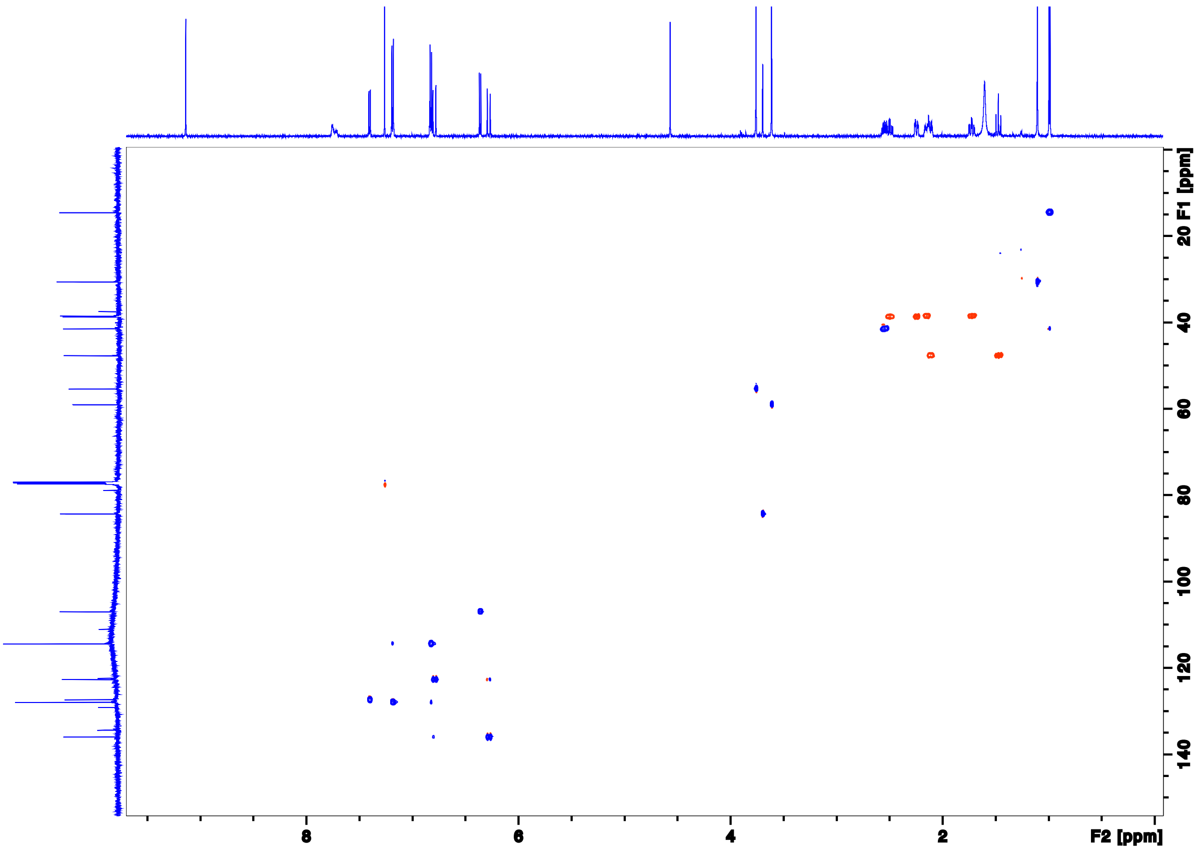

Figure S28. HSQC NMR ($CDCl_3$) spectrum for aflaquinolone H (**11**).

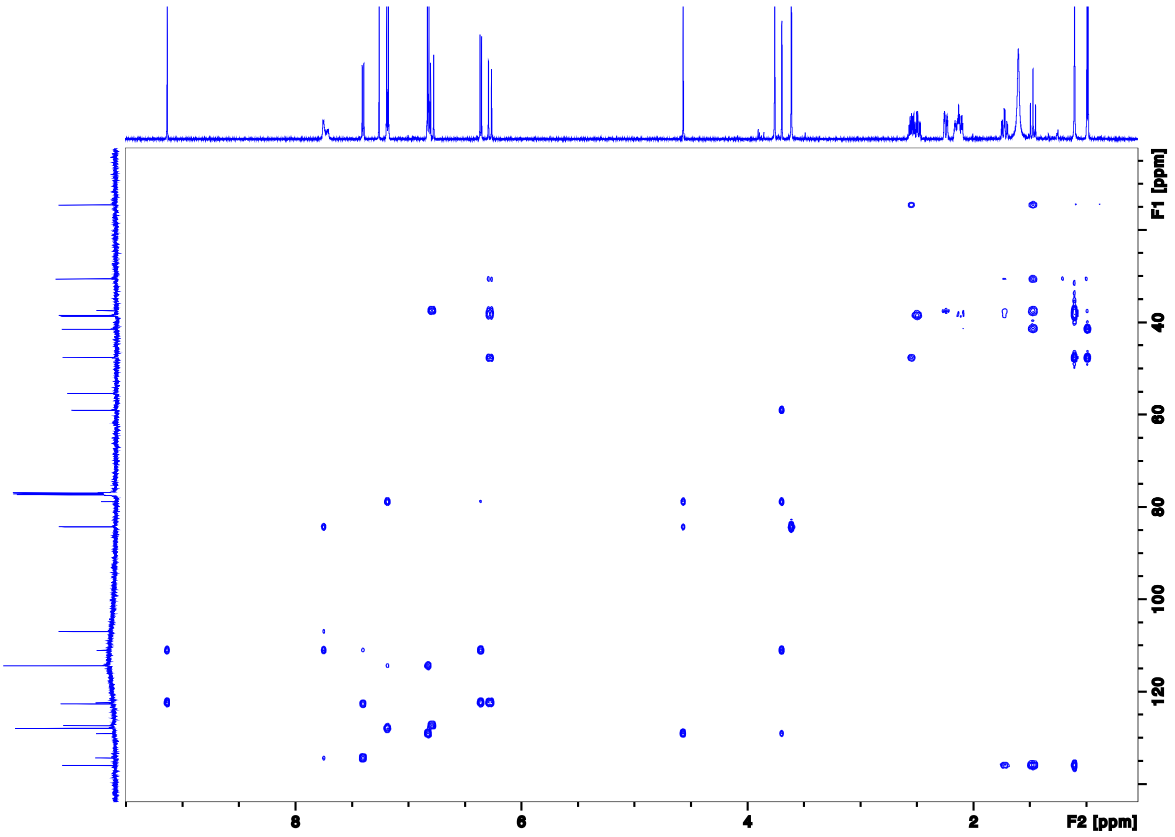

Figure S29. HMBC NMR ($CDCl_3$) spectrum for aflaquinolone H (**11**).

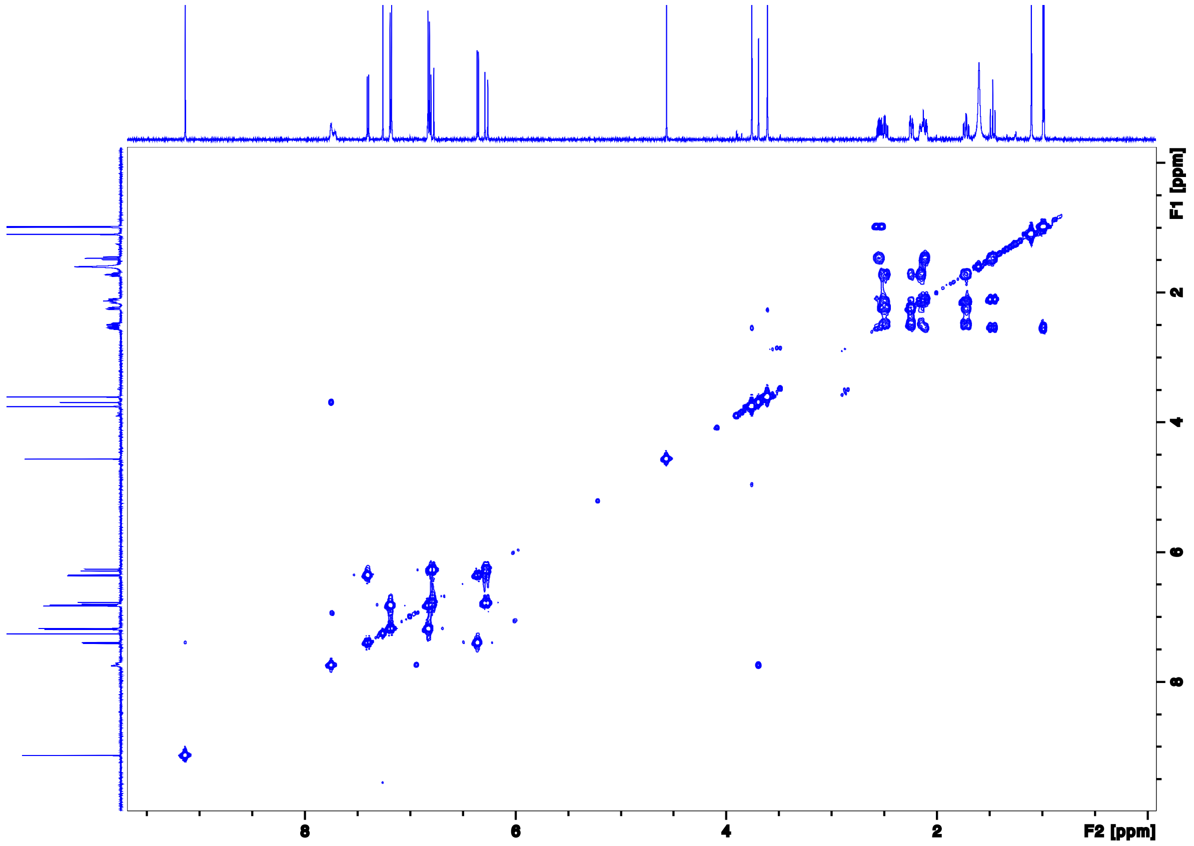

Figure S30. COSY NMR ($CDCl_3$) spectrum for aflaquinolone H (**11**).

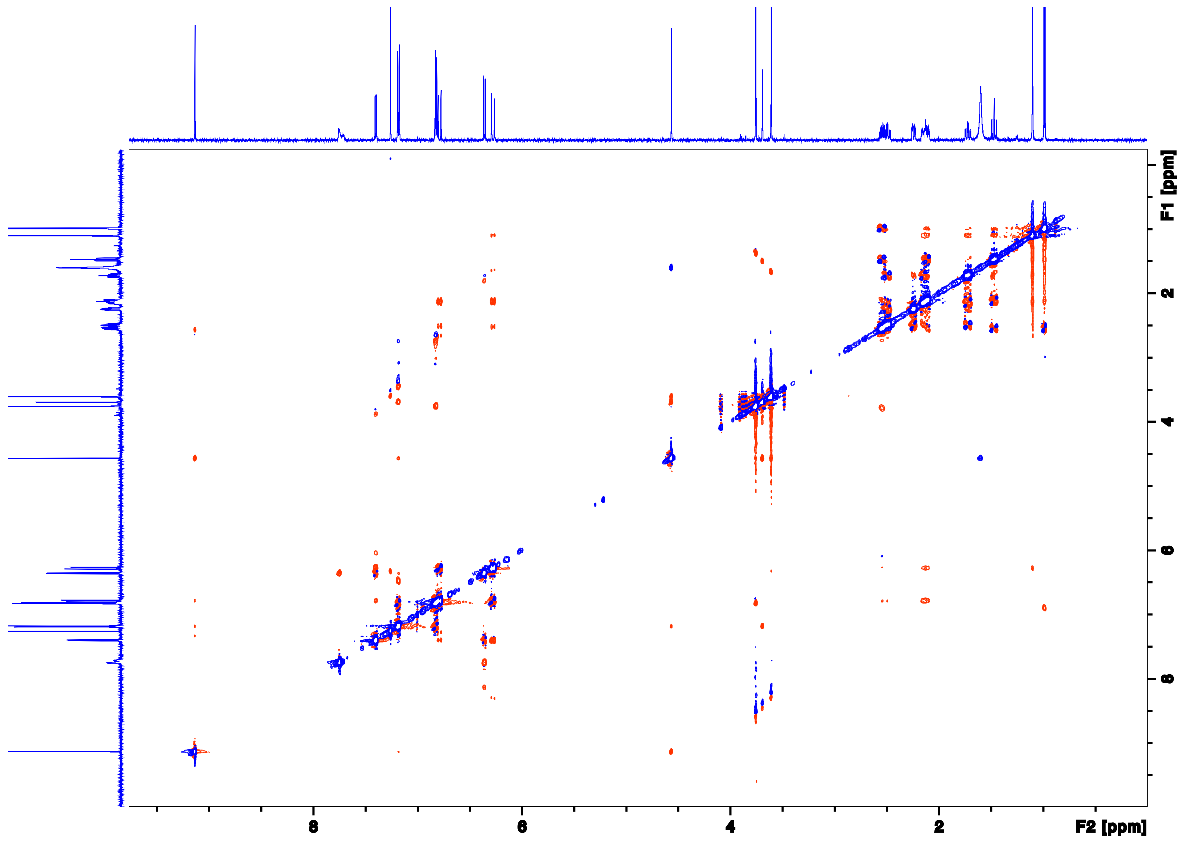

Figure S31. ROESY NMR ($CDCl_3$) spectrum for aflaquinolone H (**11**).

Mass Spectrum Molecular Formula Report

Analysis Info

Analysis Name	D:\Data\Taizong\MRF324_RICE_F21_lh20_f20_vial 30_2.d	Acquisition Date	4/8/2020 5:26:39 PM
Method	tune-medhigh_AP.m	Operator	a.salim
Sample Name	MRF324_RICE_F21_lh20_f20_vial 30_2	Instrument / Ser#	micrOTOF 213750.00232
Comment			

Acquisition Parameter

Source Type	ESI	Ion Polarity	Positive	Set Nebulizer	0.5 Bar
Focus	Not active			Set Dry Heater	180 °C
Scan Begin	100 m/z	Set Capillary	4500 V	Set Dry Gas	5.0 l/min
Scan End	1500 m/z	Set End Plate Offset	-500 V	Set Divert Valve	Source

Generate Molecular Formula Parameter

Formula, min.		
Formula, max.		
Measured m/z	Tolerance	Charge
Check Valence	Minimum	Maximum
Nirogen Rule	Electron Configuration	
Filter H/C Ratio	Minimum	Maximum
Estimate Carbon		

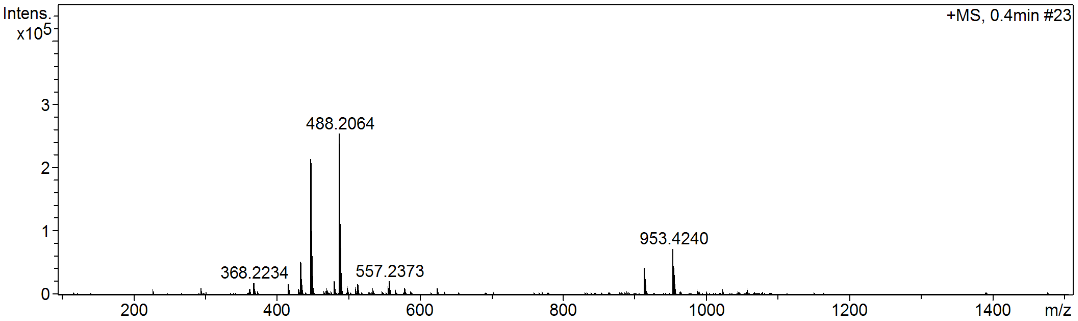

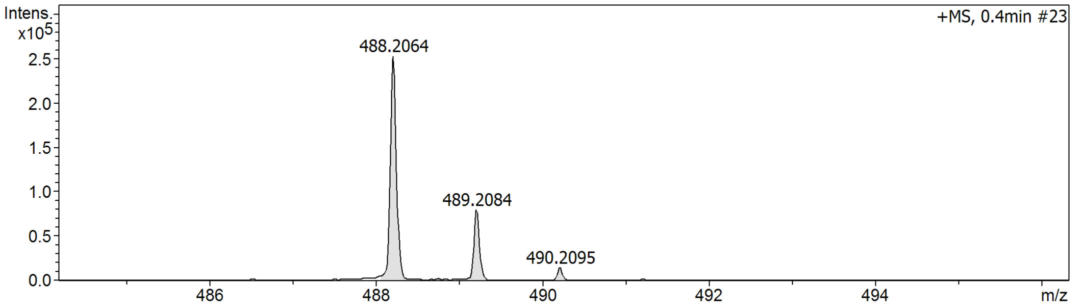

Meas. m/z	#	Ion Formula	m/z	err [ppm]	mSigma	# Sigma	Score	rdb	e⁻ Conf	N-Rule
488.2064	1	C27H31NNaO6	488.2044	-4.1	5.8	1	42.96	12.5	even	ok
	2	C28H27N5NaO2	488.2057	1.4	6.0	2	100.00	17.5	even	ok
	3	C24H23N11Na	488.2030	6.9	7.6	3	12.35	18.5	even	ok
	4	C33H27N3Na	488.2097	-6.9	29.6	4	7.68	21.5	even	ok

Bruker Compass DataAnalysis 4.1 printed: 4/8/2020 5:53:05 PM Page 1 of 1

Figure S32. HRMS spectrum and measurement for aflaquinolone H (**11**).

4.6 Aflaquinolone I (12)

Table S8. 1D and 2D NMR (600 MHz, $CDCl_3$) data for aflaquinolone I (**12**)

Pos.	δ_H, mult, (*J* in Hz)	δ_C	COSY	Key ROESY	HMBC
2		165.4			
3	3.69, br d (1.4)	84.4		4-OH, 12/16	2, 3-OMe, 4, 5
3-OMe	3.60, s	59.0			3
4		78.9			
5		111.0			
6		155.0			
7		123.0			
8	7.37, d (8.1)	127.1	9		6, 10, 17
9	6.32, d (8.1)	106.8	8		5, 7
10		134.0			
11		129.2			
12	7.18, d (8.8)	128.0	13	3	4, 14, 16
13	6.83, d (8.8)	114.5	12		11, 14, 15
14		160.4			
14-OMe	3.76, s	55.5			14
15	6.83, d (8.8)	114.5	16		
16	7.18, d (8.8)	128.0	15	3	
17	6.61, d (16.8)	121.8	18		6, 8, 19
18	6.14, d (16.8)	137.7	17		7, 20, 24, 25
19		37.2			
20	a. 1.74, m	46.1	20b, 21		18
	b. 1.07, m		20a, 21		
21	1.57[A]	36.3	20a, 20b, 22, 26		
22	3.12, ddd (10.5, 10.5, 4.5)	77.3	21, 23a, 23b		
23	a. 1.77, m	32.1	22, 23b, 24a, 24b		
	b. 1.51[A]		22, 23a, 24a, 24b		
24	a. 1.82, m	36.9	23a, 23b, 24b		
	b. 1.37, td (13.7, 3.5)		23a, 23b, 24a		
25	1.03, s	31.3			18, 20, 24
26	0.98, d (6.4)	18.8			20, 21, 22
1-NH	7.35, br s				3, 5
4-OH	4.54, s			3, 3-OMe	4, 11
6-OH	9.08, s				6, 7

[A]Resonances obscured by solvent, observed by HSQC.

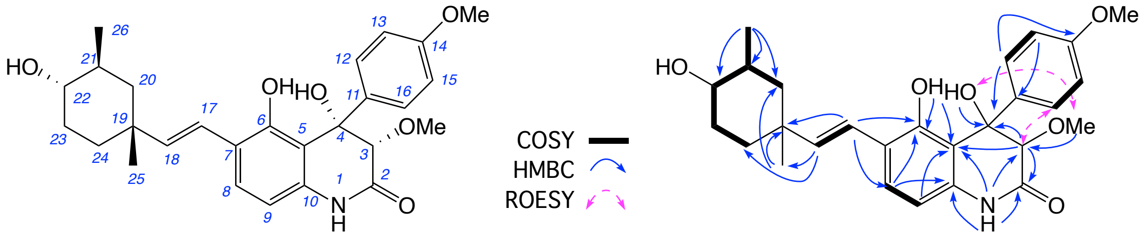

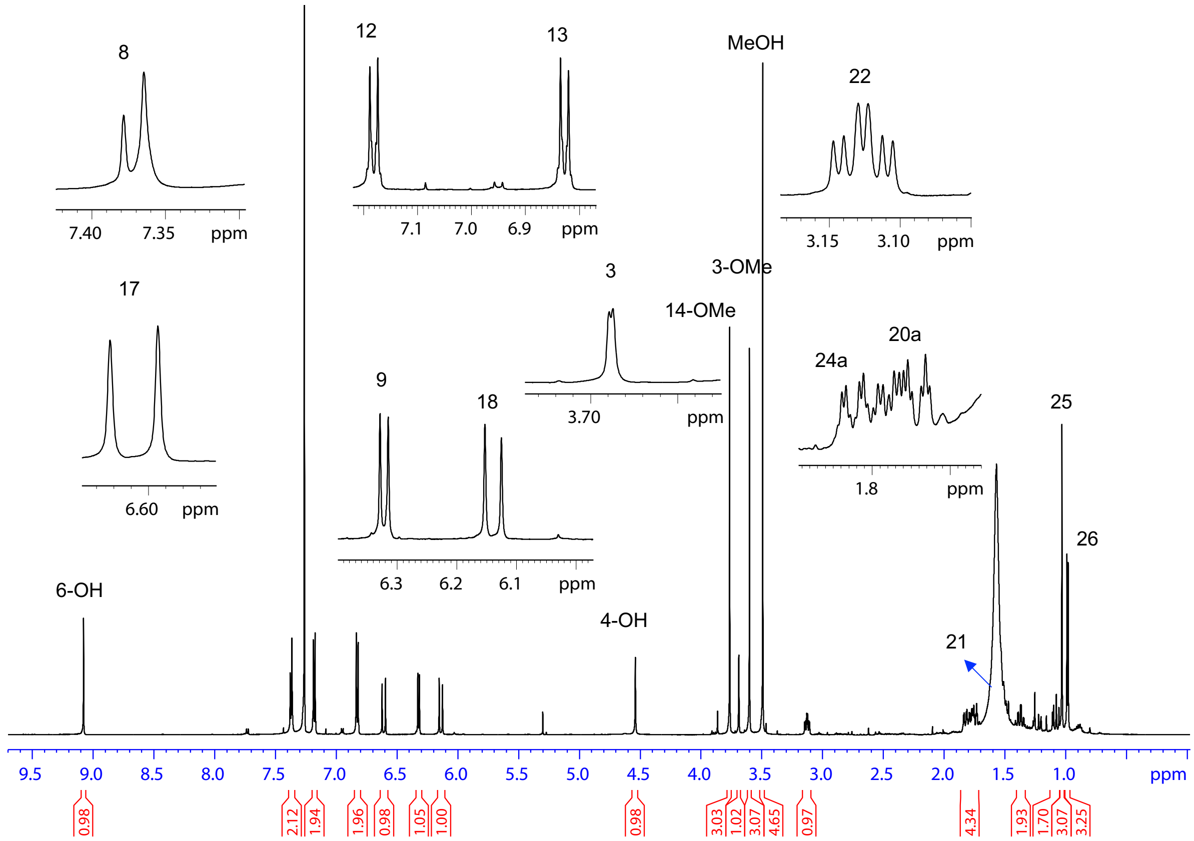

Figure S33. ^{1}H NMR (600 MHz, $CDCl_3$) spectrum for aflaquinolone I (**12**).

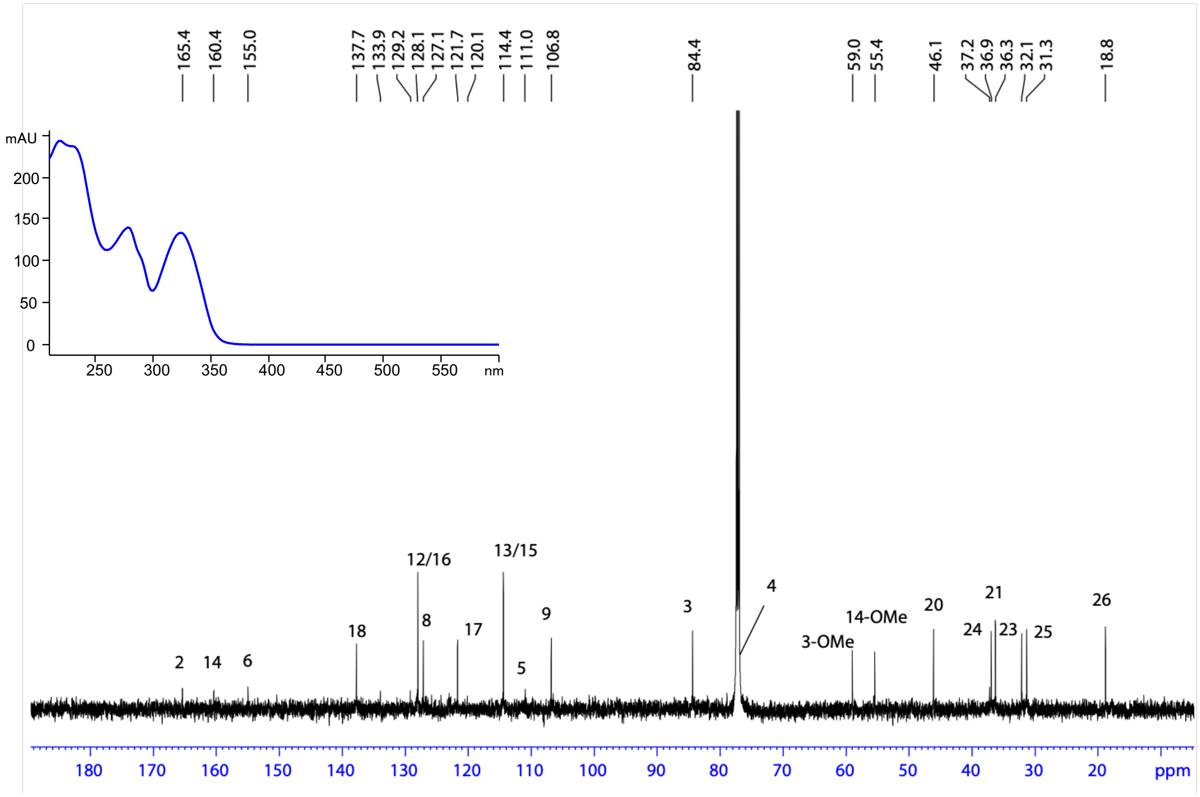

Figure S34. ^{13}C NMR (150 MHz, $CDCl_3$) and UV-vis (inset) spectra for aflaquinonlone I (**12**).

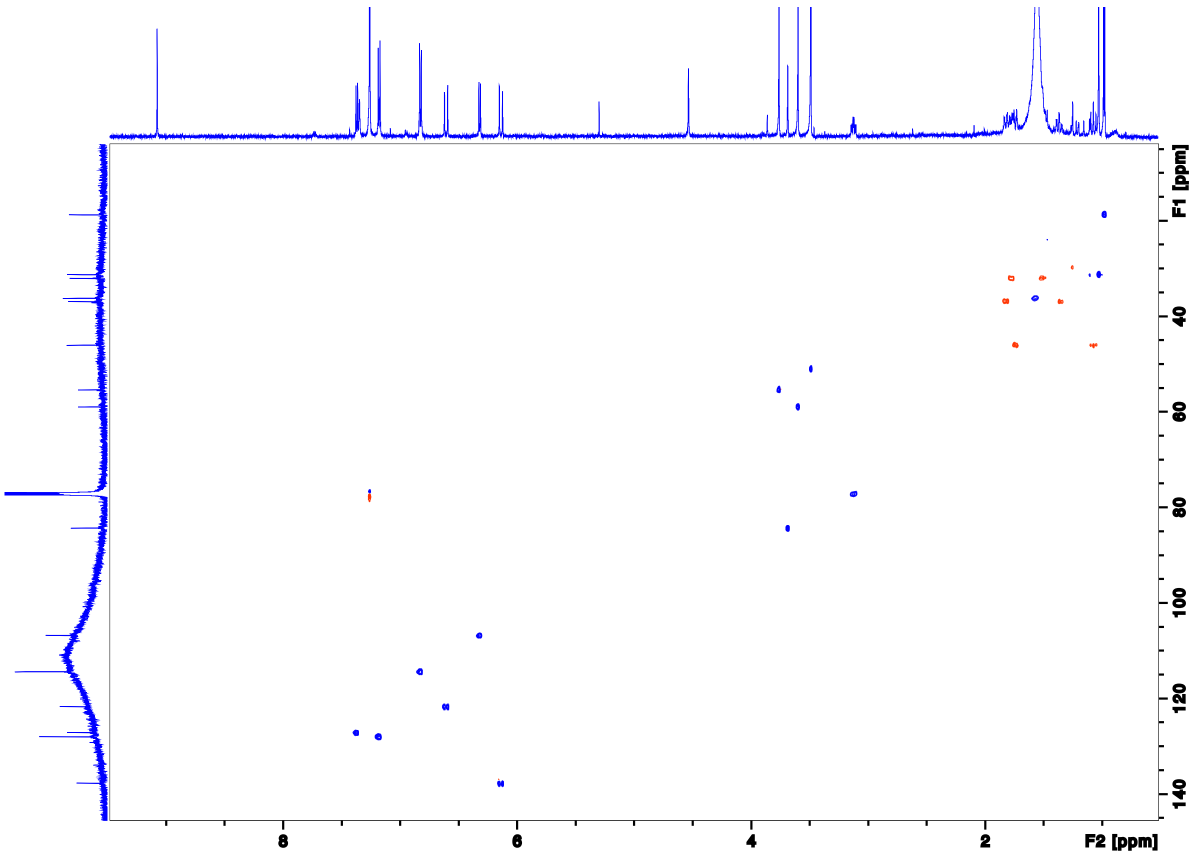

Figure S35. HSQC NMR ($CDCl_3$) spectrum for aflaquinolone I (**12**).

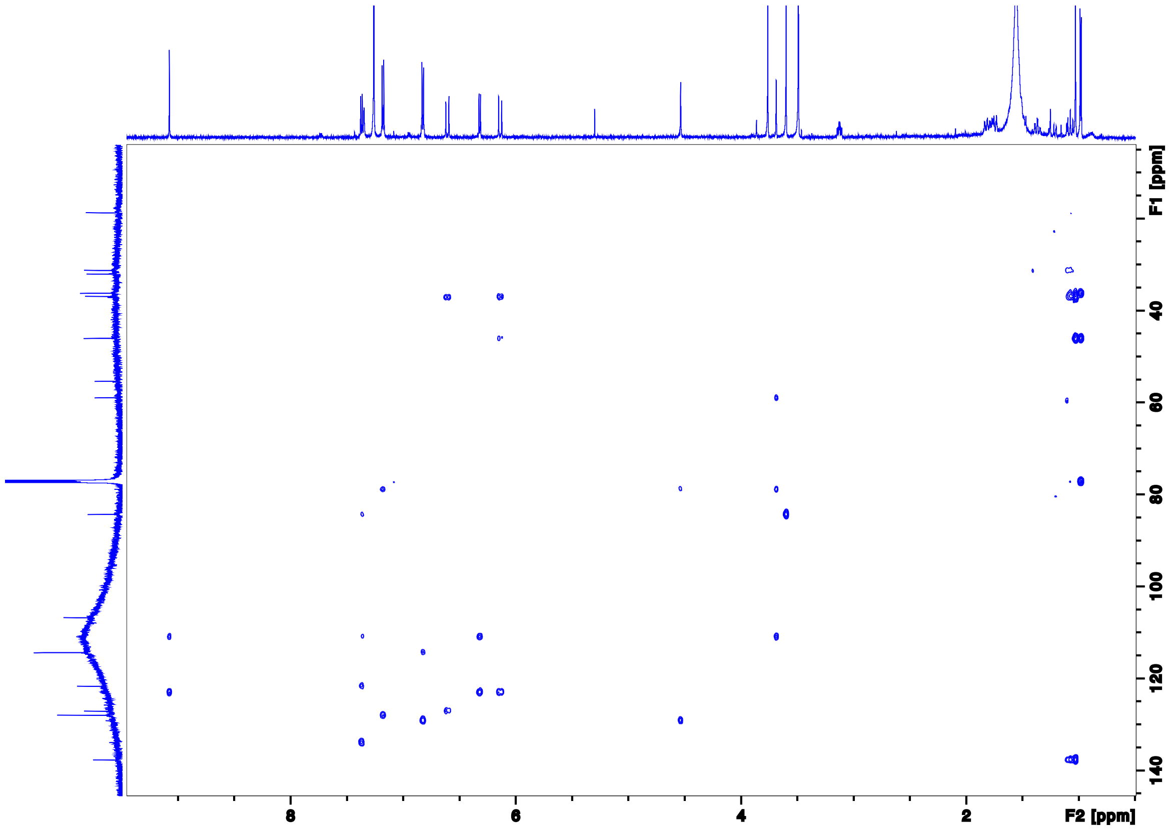

Figure S36. HMBC NMR ($CDCl_3$) spectrum for aflaquinolone I (**12**).

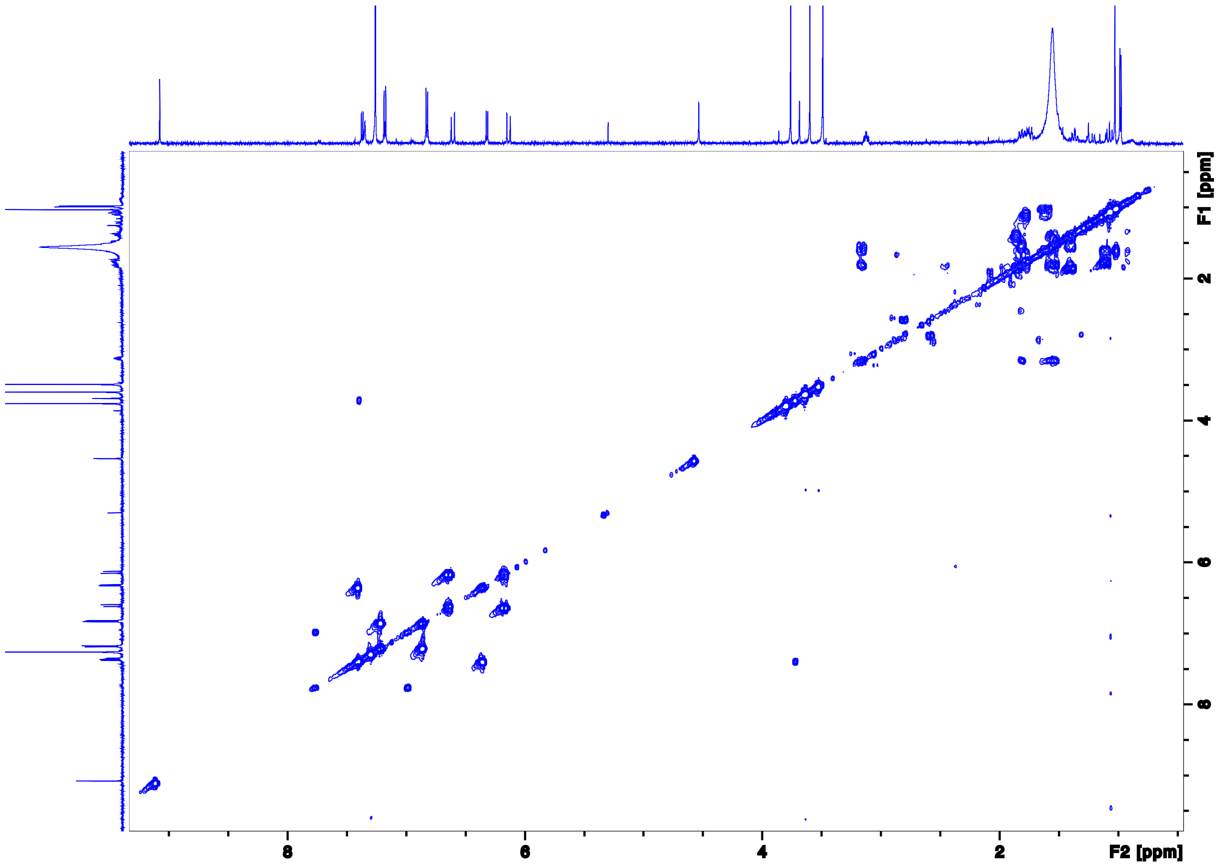

Figure S37. COSY NMR ($CDCl_3$) spectrum for aflaquinolone I (**12**).

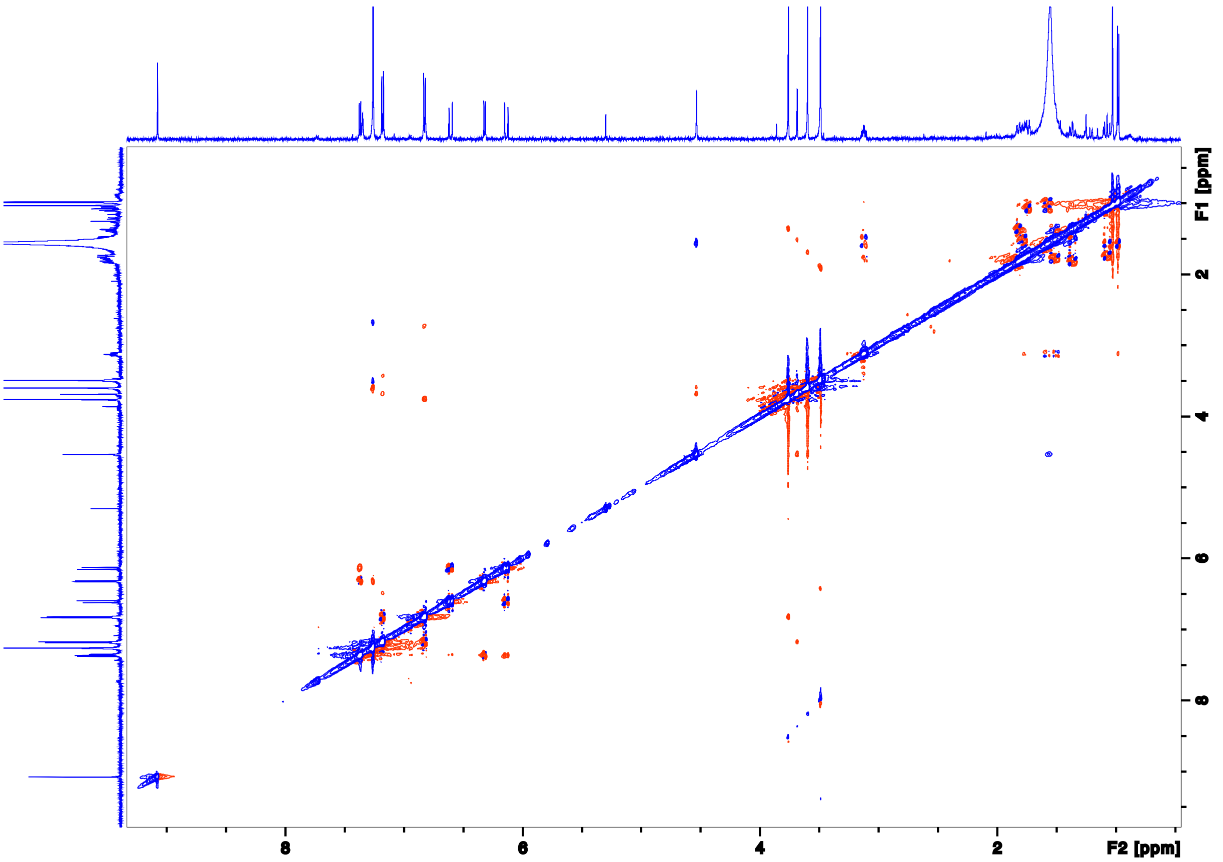

Figure S38. ROESY NMR ($CDCl_3$) spectrum for aflaquinolone I (**12**).

Mass Spectrum Molecular Formula Report

Analysis Info

Analysis Name	D:\Data\Taizong\MRF324_RICE_F21_lh20_f20_vial 12.d	Acquisition Date	4/8/2020 5:32:03 PM
Method	tune-medhigh_AP.m	Operator	a.salim
Sample Name	MRF324_RICE_F21_lh20_f20_vial 12	Instrument / Ser#	micrOTOF 213750.00232
Comment			

Acquisition Parameter

Source Type	ESI	Ion Polarity	Positive	Set Nebulizer	0.5 Bar
Focus	Not active			Set Dry Heater	180 °C
Scan Begin	100 m/z	Set Capillary	4500 V	Set Dry Gas	5.0 l/min
Scan End	1500 m/z	Set End Plate Offset	-500 V	Set Divert Valve	Source

Generate Molecular Formula Parameter

Formula, min.
Formula, max.
Measured m/z — Tolerance — Charge
Check Valence — Minimum — Maximum
Nirogen Rule — Electron Configuration
Filter H/C Ratio — Minimum — Maximum
Estimate Carbon

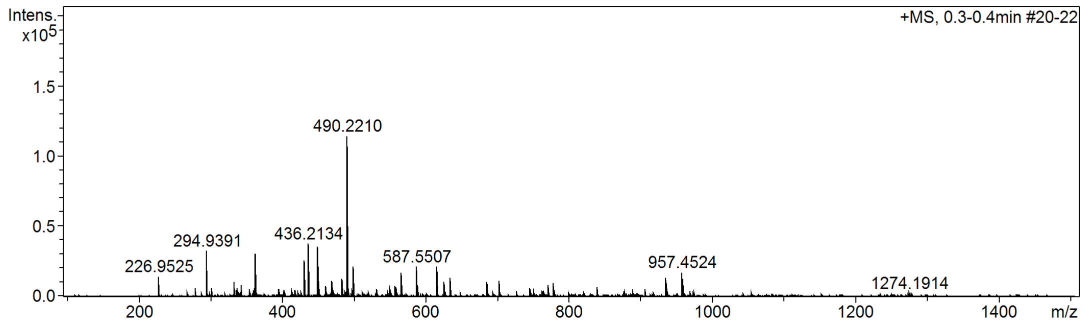

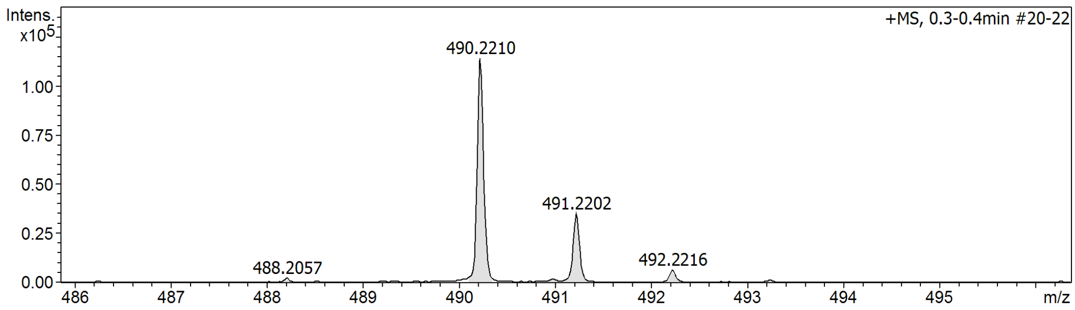

Meas. m/z	#	Ion Formula	m/z	err [ppm]	mSigma	# Sigma	Score	rdb	e⁻ Conf	N-Rule
490.2210	1	C27H33NNaO6	490.2200	2.0	2.9	1	81.19	11.5	even	ok
	2	C24H25N11Na	490.2187	-4.8	5.8	2	30.24	17.5	even	ok
	3	C28H29N5NaO2	490.2213	0.7	9.5	3	100.00	16.5	even	ok
	4	C23H29N7NaO4	490.2173	-7.5	14.4	4	7.03	12.5	even	ok

Bruker Compass DataAnalysis 4.1 printed: 4/8/2020 5:54:16 PM Page 1 of 1

Figure S39. HRMS spectrum and measurement for aflaquinolone I (**12**).

4.7 Terrecyclic acid A (13)

Table S9. 1D and 2D NMR (600 MHz, DMSO-d_6) data for terrecyclic acid A (**13**)

Pos.	δ_H, mult, (J in Hz)	δ_C	COSY	ROESY	HMBC
1		54.5			
2	2.92, dd (10.6, 9.4)	45.9	3a, 3b		8, 10, 12, 13
3	a. 2.55, dd (10.2, 11.7)	41.1	2, 3b	10b, 11, 14	2, 4, 11
	b. 2.45, dd (19.2, 9.4)		2, 3a		1, 4, 5
4		206.6			
5		151.7			
6	a. 5.72, s	114.5	6b	6b	1, 4, 5
	b. 5.17, s		6a	6a	1, 4, 5
7		176.0			
8	2.99, d (8.2)	47.2	9a, 9b	9a, 12a	1, 2, 7, 9, 10, 12
9	a. 2.10, m	22.3	8, 9b, 10a, 10b	8, 15	7, 8, 10
	b. 1.74[A]		8, 9a, 10a, 10b		1, 7, 10, 11
10	a. 1.74[A]	28.9	9, 10b, 11		11
	b. 1.65[B]		9, 10a		8
11	1.88, br s	48.4	10a	10a, 10b, 14, 15	1, 9, 12
12	a. 1.80, d (14.4)	53.1	12b	8, 14, 15	1, 8, 13, 14, 15
	b. 1.63[B], d (14.4)		12a	14	1, 5, 8, 13, 14, 15
13		40.0			
14	1.19, s	34.6		3, 11, 12a, 12b	11, 12, 13, 15
15	1.14, s	27.1		9a, 11, 12a	11, 12, 13, 14

[A-B] Resonances with same letter overlapped.

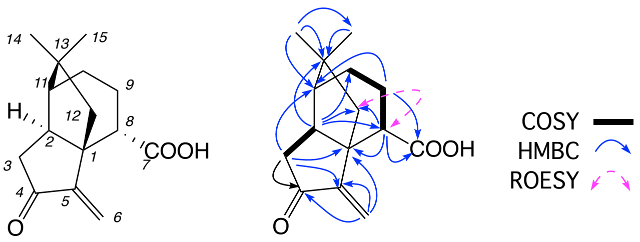

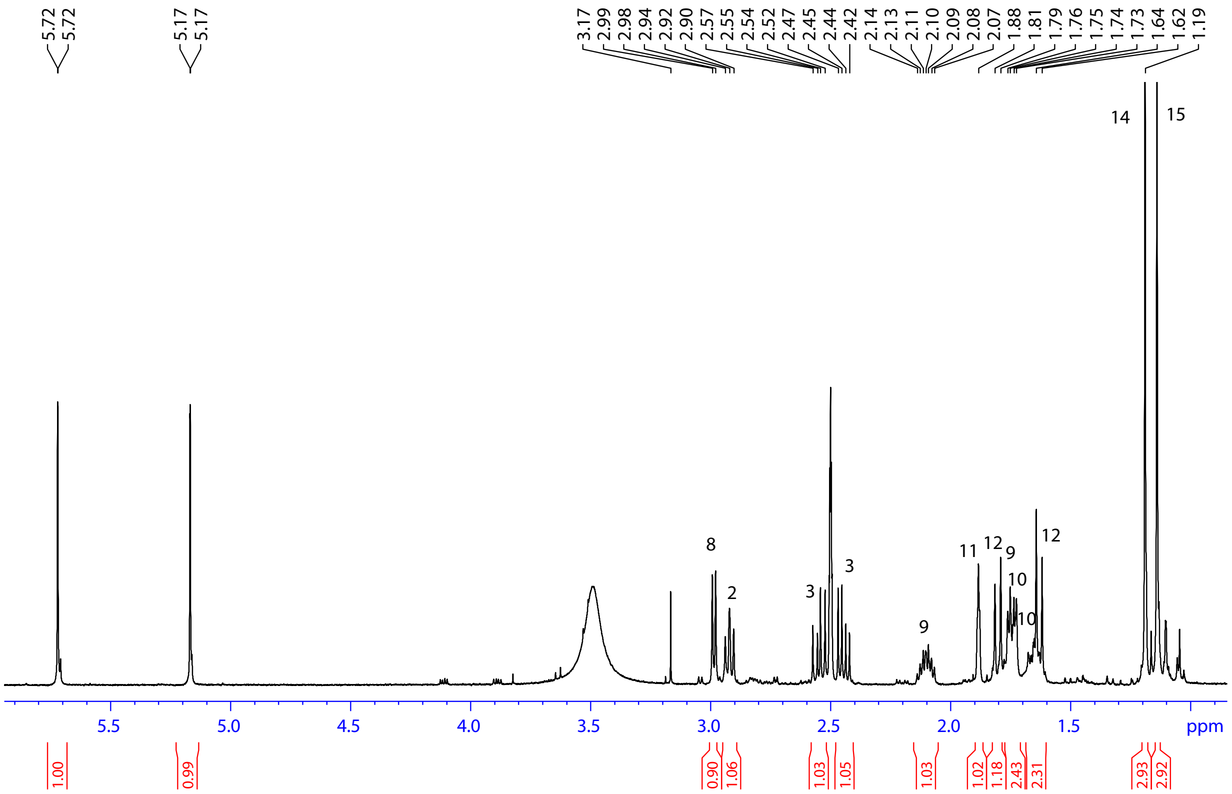

Figure S40. ^{1}H NMR (600 MHz, DMSO-d_6) spectrum for terrecyclic acid A (**13**).

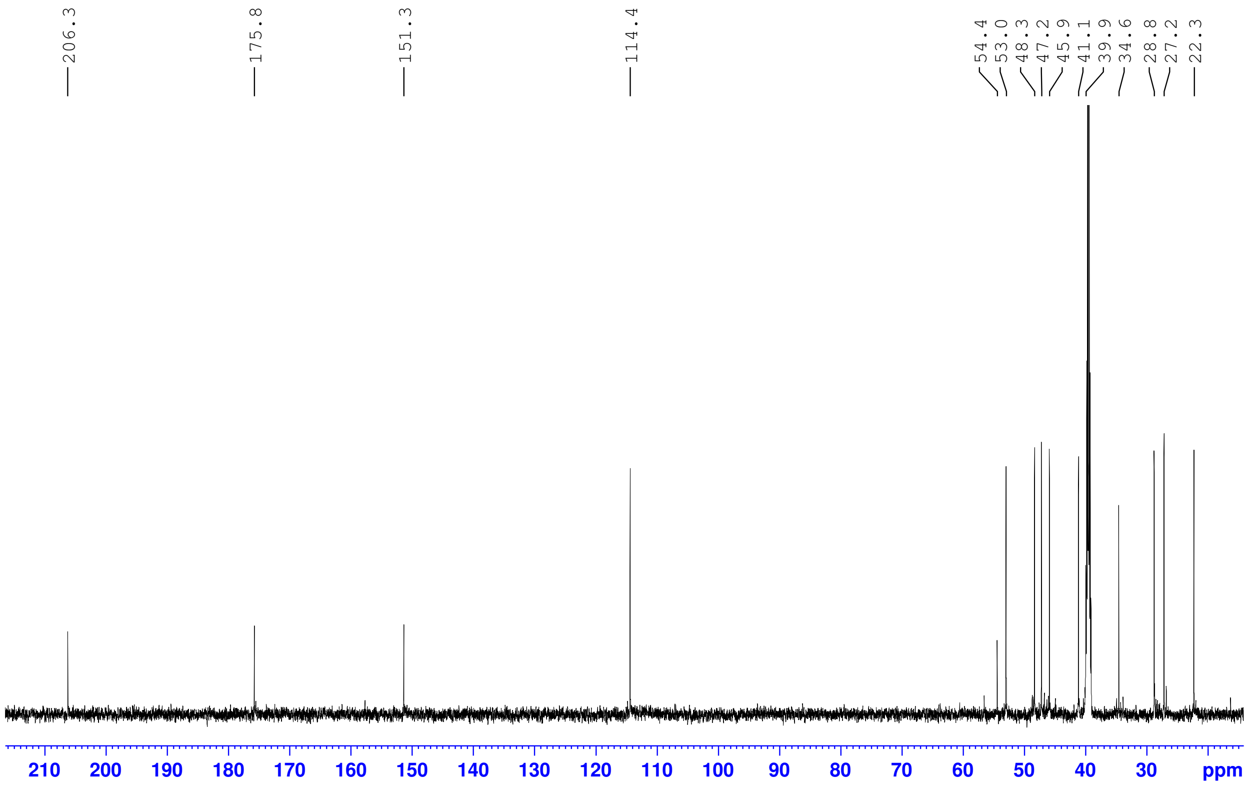

Figure S41. ^{13}C NMR (150 MHz, DMSO-d_6) spectrum for terrecyclic acid A (**13**).

Mass Spectrum Molecular Formula Report

Analysis Info

Analysis Name	D:\Data\Taizong\MRF324_rice_fr17_vial 6.d	Acquisition Date	3/24/2020 10:19:30 AM
Method	tune-medhigh_AP.m	Operator	a.salim
Sample Name	MRF324_rice_fr17_vial 6	Instrument / Ser#	micrOTOF 213750.00232
Comment			

Acquisition Parameter

Source Type	ESI	Ion Polarity	Positive	Set Nebulizer	0.5 Bar
Focus	Not active			Set Dry Heater	180 °C
Scan Begin	100 m/z	Set Capillary	4500 V	Set Dry Gas	5.0 l/min
Scan End	1500 m/z	Set End Plate Offset	-500 V	Set Divert Valve	Source

Generate Molecular Formula Parameter

Formula, min.		
Formula, max.		
Measured m/z	Tolerance	Charge
Check Valence	Minimum	Maximum
Nirogen Rule	Electron Configuration	
Filter H/C Ratio	Minimum	Maximum
Estimate Carbon		

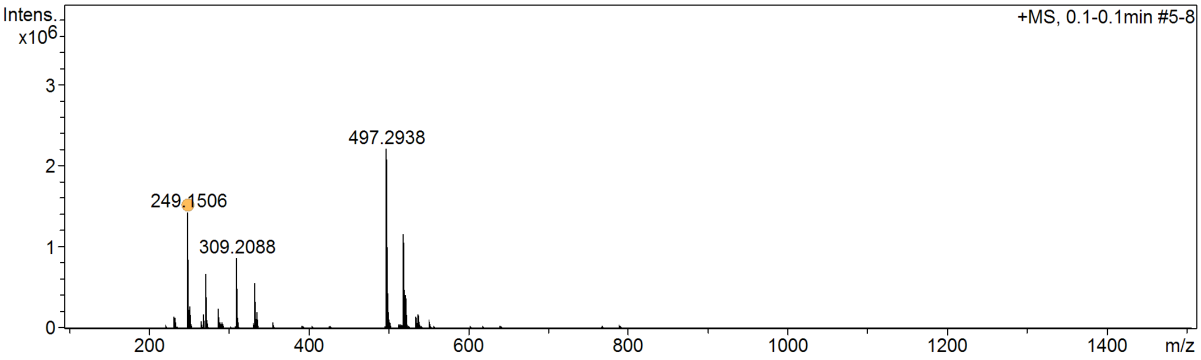

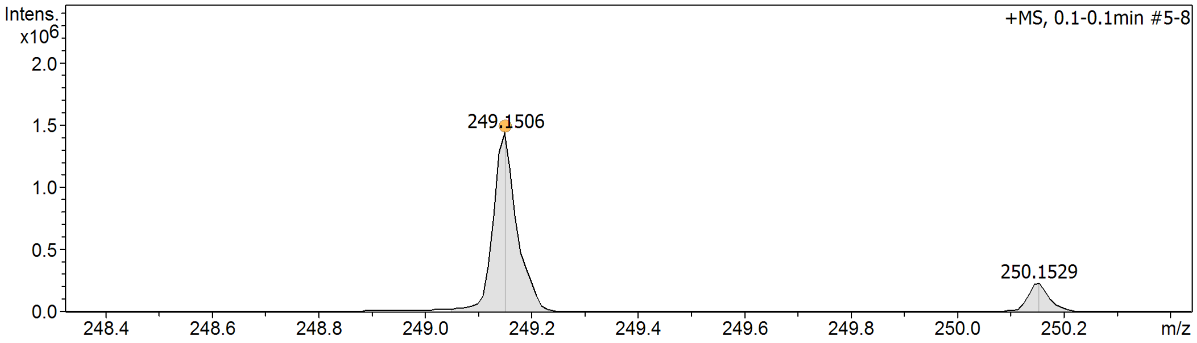

Meas. m/z	#	Ion Formula	m/z	err [ppm]	mSigma	# Sigma	Score	rdb	e¯ Conf	N-Rule
249.1506	1	C15H21O3	249.1485	8.5	96.2	1	100.00	5.5	even	ok

Bruker Compass DataAnalysis 4.1 printed: 3/24/2020 10:25:43 AM Page 1 of 1

Figure S42. HRMS spectrum and measurement for terrecyclic acid A (**13**).

4.8 Aspulvinone Y (14)

Table S10. 1D and 2D NMR (600 MHz, methanol-d_4) data for aspulvinone Y (**14**)

Pos.	δ_H, mult, (J in Hz)	δ_C	COSY	HMBC
1		-		
2		101.6		
3		164.2		
4		142.3		
5	6.38, s	108.0		3, 4, 2″, 6″
1′		122.6		
2′	7.64, d (1.5)	129.7		2, 6′, 4′, 7′
3′		121.7		
4′		154.3		
5′	6.73, d (8.3)	117.6	6′	1′, 4′, 3′
6′	7.62, dd (8.3, 1.5)	127.7	5′	2, 4′, 2′
7′	2.83, t (6.8)	23.1	8′	4′, 3′, 2′, 8′, 9′
8′	1.84, t (6.8)	33.5	7′	3′, 7′, 9′, 10′, 11′
9′		75.2		
10′	1.33, s	26.8		8′, 9′, 11′
11′	1.33, s	26.8		8′, 9′, 10′
1″		126.3		
2″	7.57, s	133.1		5, 4″, 6″, 7″
3″		121.4		
4″		155.0		
5″	6.78, d (8.6)	118.1	6″	1″, 3″, 4″
6″	7.51, d (1.5)	130.9	5″	5, 2″, 4″
7″	a. 3.07, dd (16.4, 5.0)	31.8	7″b, 8″	2″, 3″, 4″, 8″, 9″
	b. 2.78, dd (16.4, 7.3)		7″a, 8″	2″, 3″, 4″, 8″, 9″
8″	3.79, dd (7.3, 5.0)	69.9	7″a, 7″b	3″, 10″, 11″
9″		78.5		
10″	1.29, s	21.1		8″, 9″, 11″
11″	1.35, s	25.6		8″, 9″, 10″

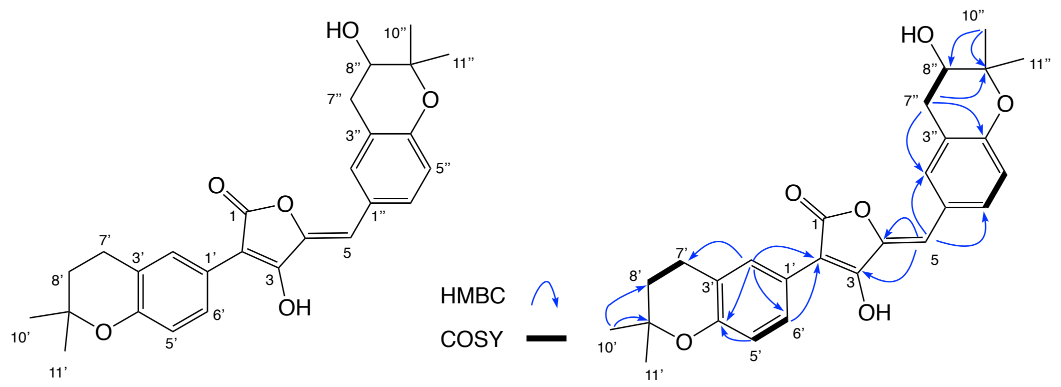

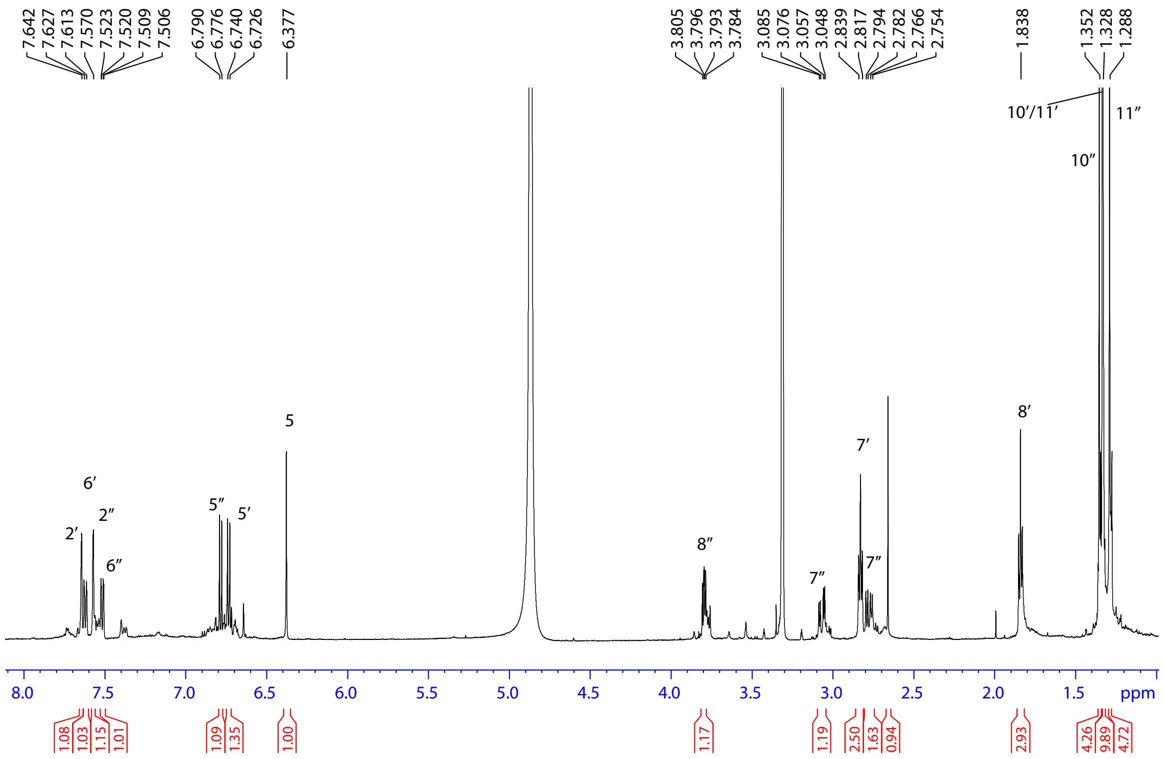

Figure S43. ^{1}H NMR (600 MHz, acetone-d_6) spectrum for aspulvinone Y (**14**).

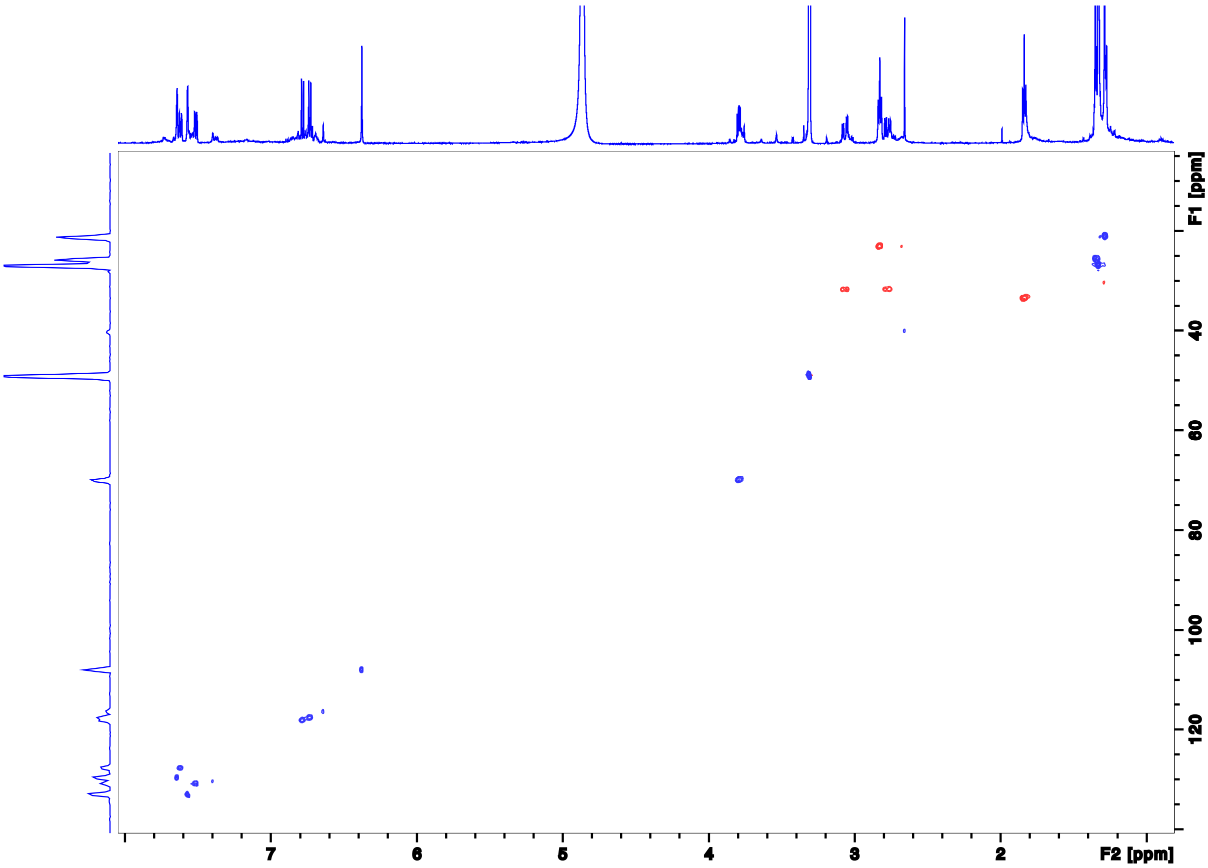

Figure S44. HSQC NMR (600 MHz, acetone-d_6) spectrum for aspulvinone Y (**14**).

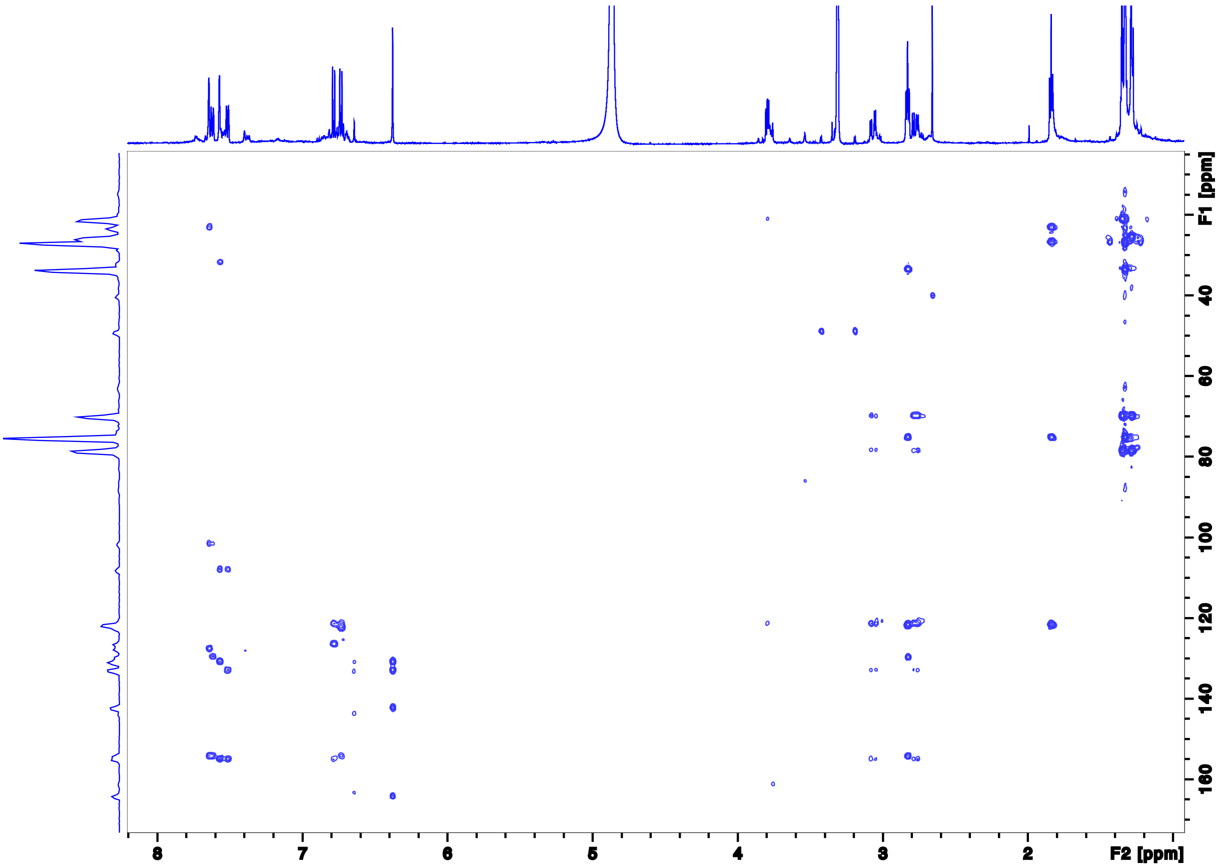

Figure S45. HMBC NMR (600 MHz, acetone-d_6) spectrum for aspulvinone Y (**14**).

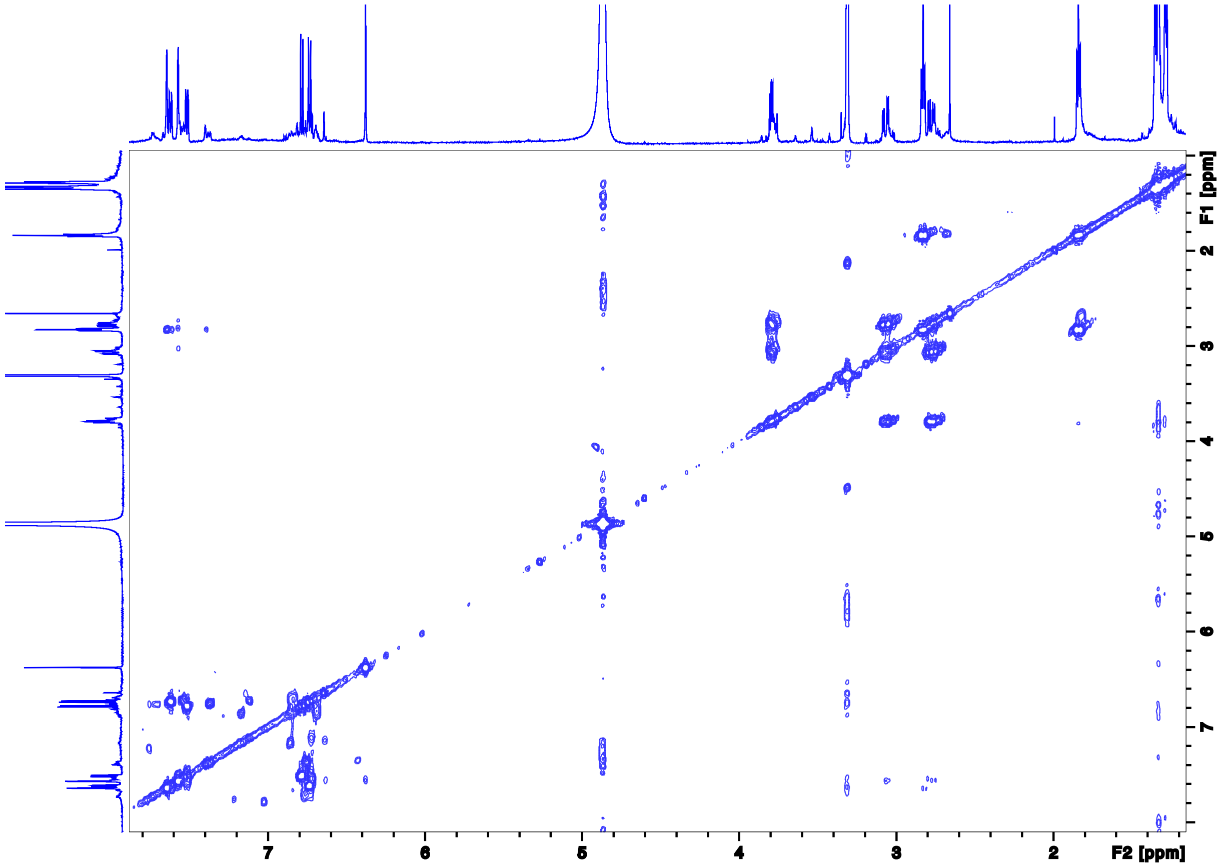

Figure S46. COSY NMR (600 MHz, acetone-d_6) spectrum for aspulvinone Y (**14**).

Mass Spectrum Molecular Formula Report

Analysis Info

Analysis Name	D:\Data\Taizong\MRF324_rice_F27_V14_15.d	Acquisition Date	10/9/2020 3:40:01 PM
Method	tune-medhigh_AP.m	Operator	a.salim
Sample Name	MRF324_rice_F27_V14_15	Instrument / Ser#	micrOTOF 213750.00232
Comment			

Acquisition Parameter

Source Type	ESI	Ion Polarity	Positive	Set Nebulizer	0.5 Bar
Focus	Not active			Set Dry Heater	180 °C
Scan Begin	100 m/z	Set Capillary	4500 V	Set Dry Gas	5.0 l/min
Scan End	1500 m/z	Set End Plate Offset	-500 V	Set Divert Valve	Source

Generate Molecular Formula Parameter

Formula, min.
Formula, max.
Measured m/z — Tolerance — Charge
Check Valence — Minimum — Maximum
Nirogen Rule — Electron Configuration
Filter H/C Ratio — Minimum — Maximum
Estimate Carbon

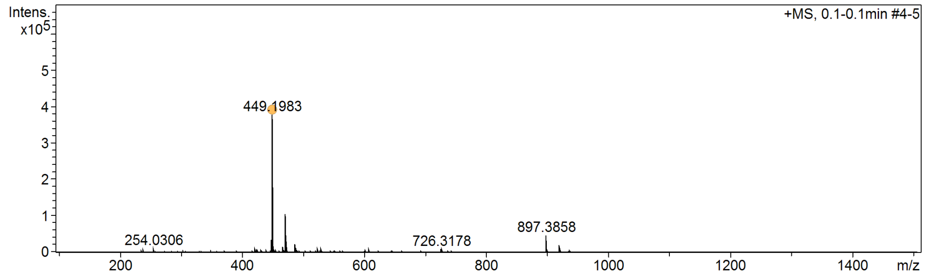

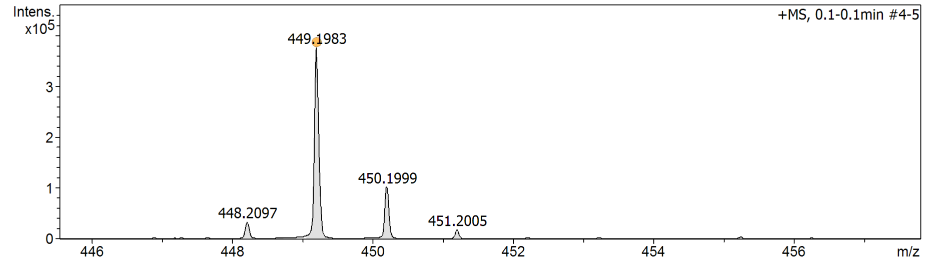

Meas. m/z	#	Ion Formula	m/z	err [ppm]	mSigma	# Sigma	Score	rdb	e⁻ Conf	N-Rule
449.1983	1	C27H29O6	449.1959	5.3	13.1	1	49.33	13.5	even	ok
	2	C24H21N10	449.1945	8.3	13.5	2	12.99	19.5	even	ok
	3	C18H21N14O	449.2017	7.7	16.8	3	12.24	15.5	even	ok
	4	C20H33O11	449.2017	7.8	24.0	4	13.80	4.5	even	ok
	5	C28H25N4O2	449.1972	2.3	24.5	5	100.00	18.5	even	ok
	6	C33H25N2	449.2012	6.6	48.1	6	12.38	22.5	even	ok

Bruker Compass DataAnalysis 4.1 printed: 10/9/2020 3:57:19 PM Page 1 of 1

Figure S47. HRMS spectrum and measurement for aspulvinone Y (**14**).

4.9 Aspulvinone N-CR (15)

Table S11. 1D and 2D NMR (600 MHz, acetone-d_6) data for aspulvinone N-CR (**15**)*

Pos.	δ_H, mult, (J in Hz)	δ_C	HMBC
1			
2		98.4	
3		164.5	
4		140.5	
5	6.24, s	105.7	3, 4, 2″, 6″
1′		111.2	
2′	7.71, s	130.1	2, 6′, 4′, 7′
3′		113.4	
4′		155.1	
5′	6.38, s	104.6	1′, 6′, 4′, 3′
6′		153.7	
7′	2.72, t (6.7)	22.2	4′, 3′, 2′, 8′, 9′
8′	1.80, t (6.7)	33.4	3′, 7′, 9′, 10′, 11′
9′		74.8	
10′	1.30, s	26.9	8′, 9′, 11′
11′	1.30, s	26.9	8′, 9′, 10′
1″		126.7	
2″	7.54, d (2.0)	132.6	5, 4″, 6″,7″
3″		121.5	
4″		154.4	
5″	6.78, d (8.5)	117.6	1″, 3″, 4″
6″	7.55, dd (8.5, 2.0)	130.2	5, 2″, 4″
7″	a. 3.05, dd (16.5, 5.2)	32.0	2″, 3″, 4″, 8″, 9″
	b. 2.78, dd (16.5, 7.8)		2″, 3″, 4″, 8″, 9″
8″	3.82, dd (7.8, 5.2)	69.4	3″
9″		78.3	
10″	1.27, s	20.7	
11″	1.36, s	25.8	

*COSY NMR data was not acquired due to fast decomposition of compound.

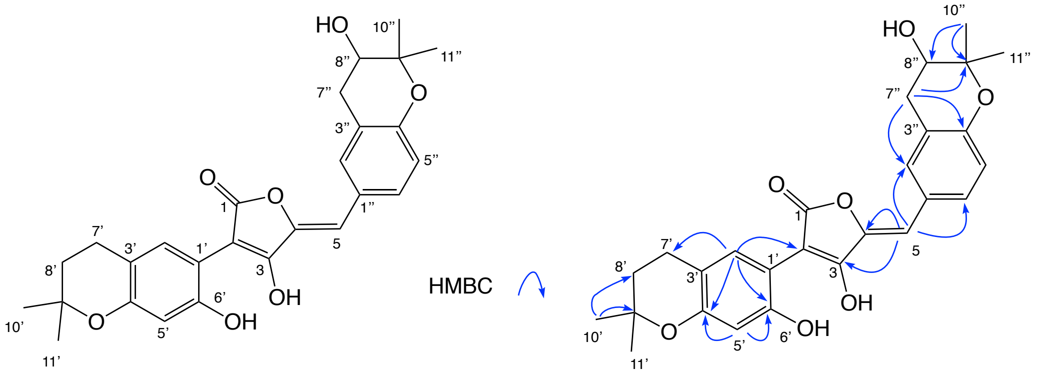

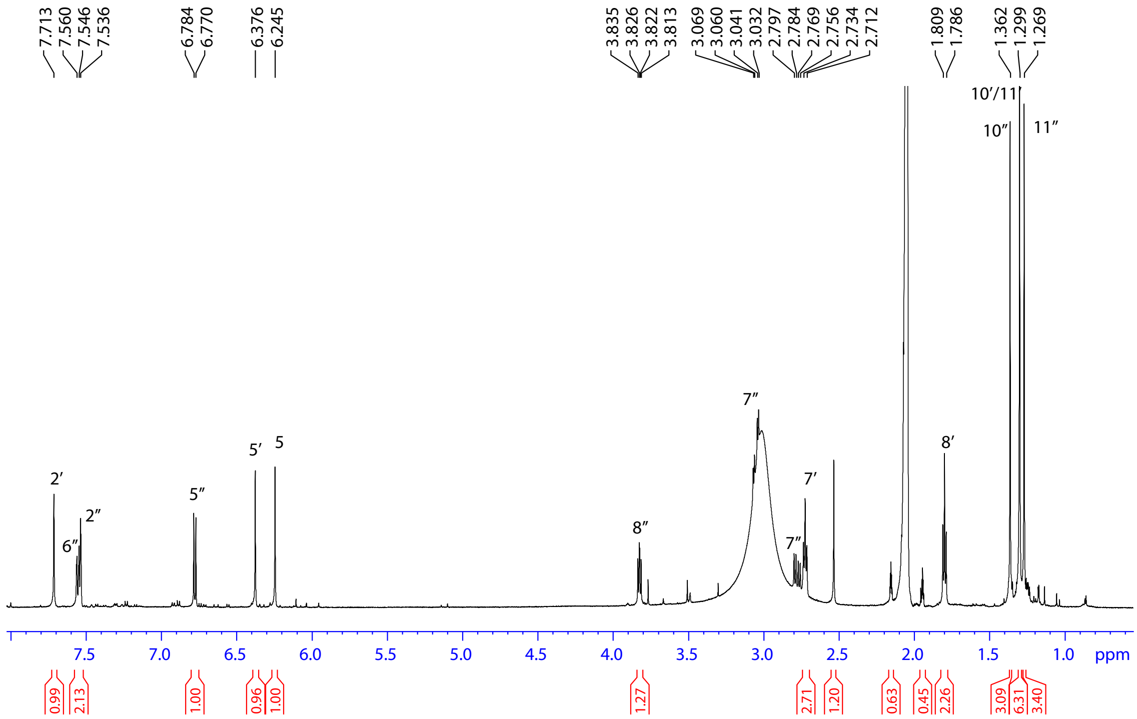

Figure S48. ^{1}H NMR (600 MHz, acetone-d_6) spectrum for aspulvinone N-CR (**15**).

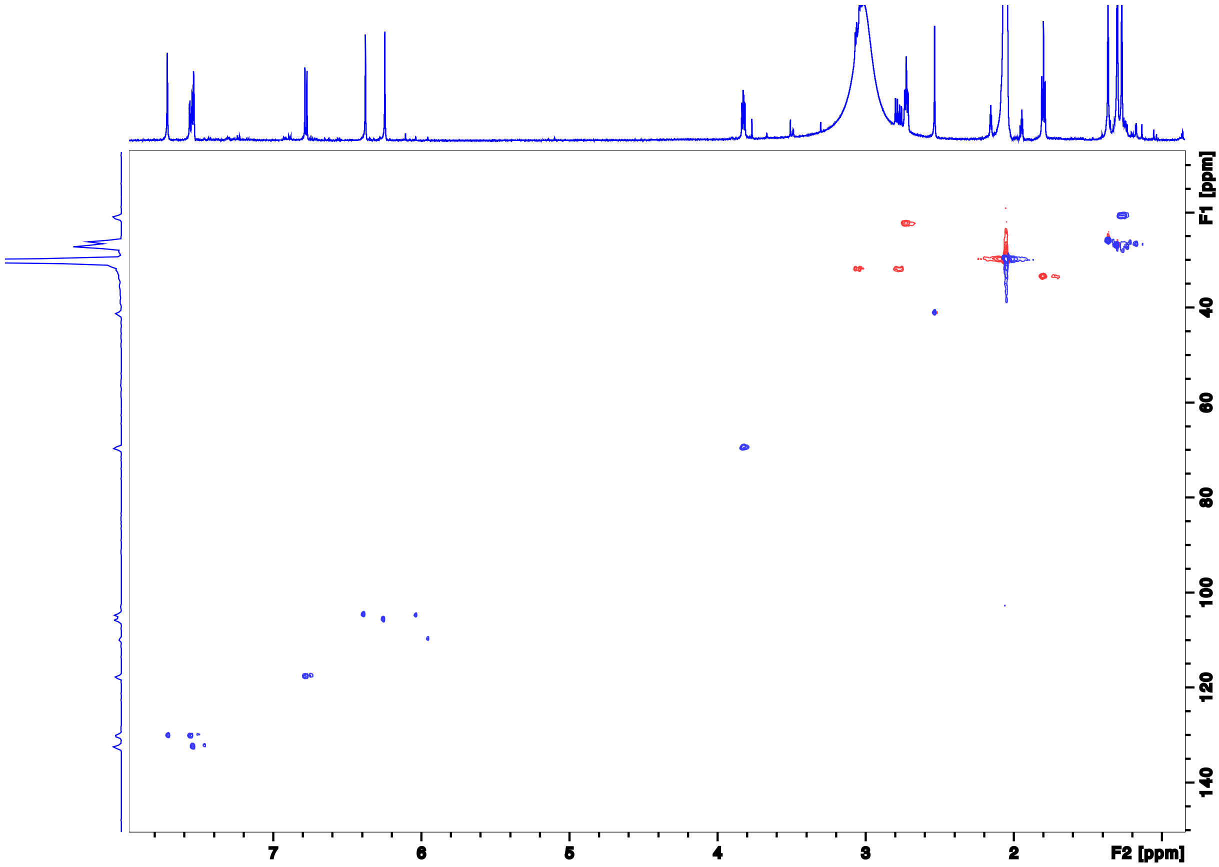

Figure S49. HSQC NMR (600 MHz, acetone-d_6) spectrum for aspulvinone N-CR (**15**).

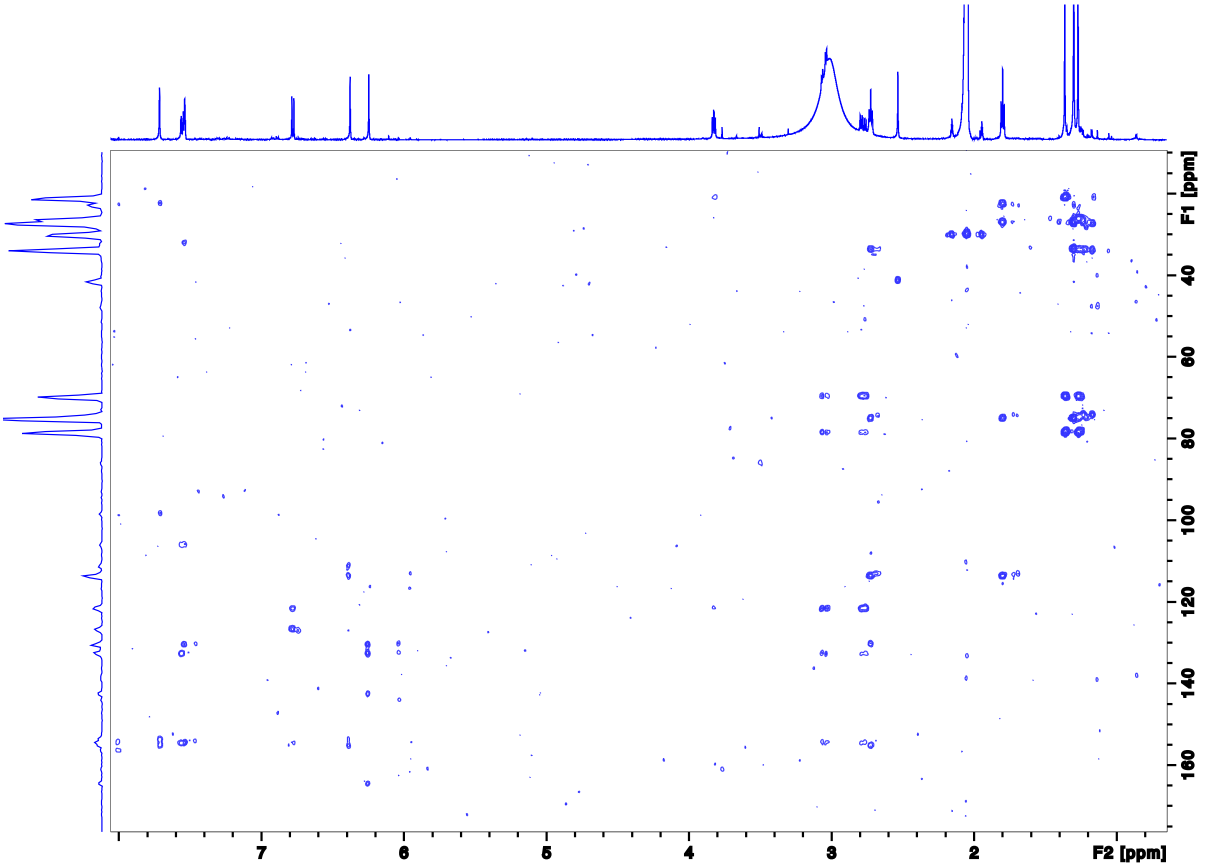

Figure S50. HMBC NMR (600 MHz, acetone-d_6) spectrum for aspulvinone N-CR (**15**).

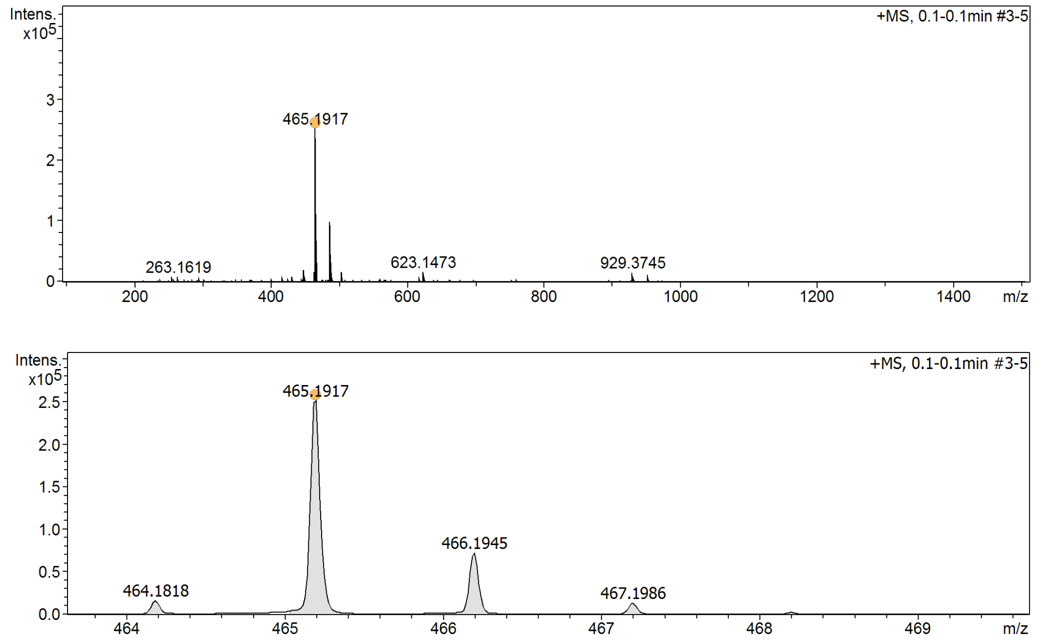

Meas. m/z	#	Ion Formula	m/z	err [ppm]	mSigma	# Sigma	Score	rdb	e⁻ Conf	N-Rule
465.1917	1	C27H29O7	465.1908	-2.0	5.4	1	95.26	13.5	even	ok
	2	C24H21N10O	465.1894	-4.9	7.0	2	36.79	19.5	even	ok
	3	C23H25N6O5	465.1881	-7.8	7.1	3	10.19	14.5	even	ok
	4	C28H25N4O3	465.1921	-0.9	16.8	4	100.00	18.5	even	ok

Figure S51. HRMS spectrum and measurement for aspulvinone N-CR (**15**).

4.10 Aspulvinone B (16)

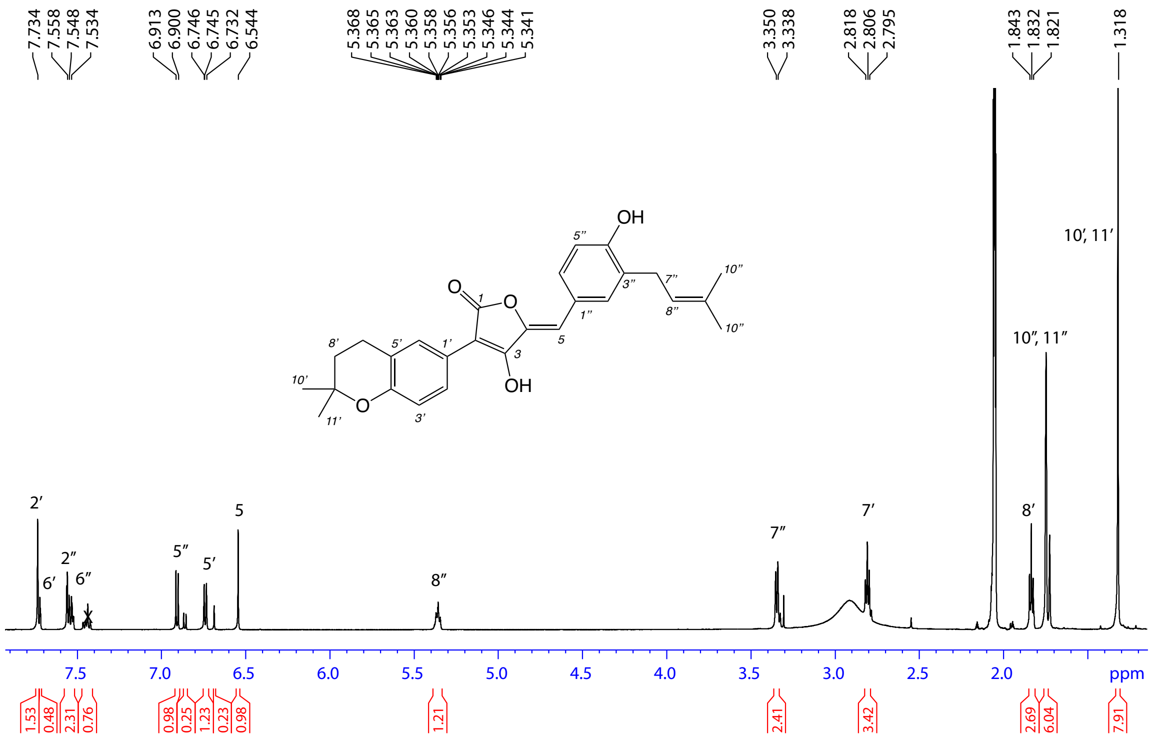

Figure S52. ^{1}H NMR (600 MHz, acetone-d_6) spectrum for aspulvinone B (**16**).

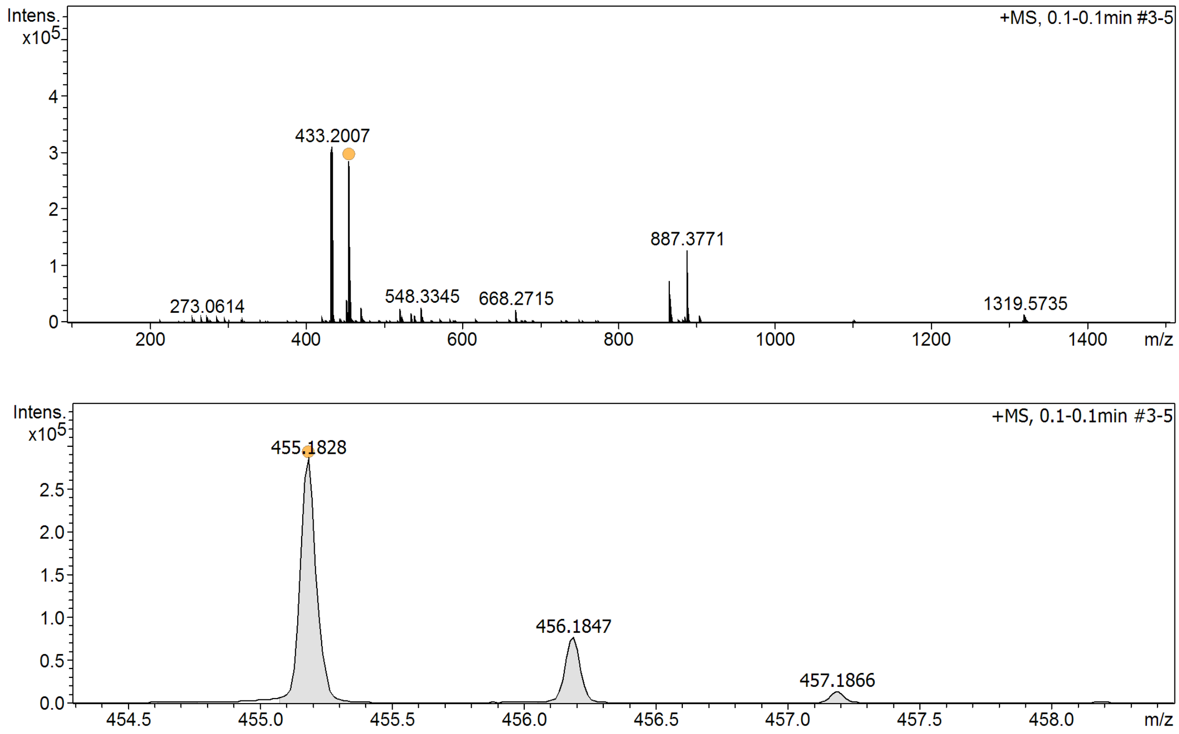

Meas. m/z	#	Ion Formula	m/z	err [ppm]	mSigma	# Sigma	Score	rdb	e⁻ Conf	N-Rule
455.1828	1	C23H24N6NaO3	455.1802	-5.6	3.7	1	25.33	14.5	even	ok
	2	C22H28N2NaO7	455.1789	-8.6	9.0	2	5.89	9.5	even	ok
	3	C27H28NaO5	455.1829	0.3	14.4	3	100.00	13.5	even	ok
	4	C28H24N4NaO	455.1842	3.2	25.9	4	38.28	18.5	even	ok

Figure S53. HRMS spectrum and measurement for aspulvinone B (**16**).

4.11 Aspulvinone D (**17**)

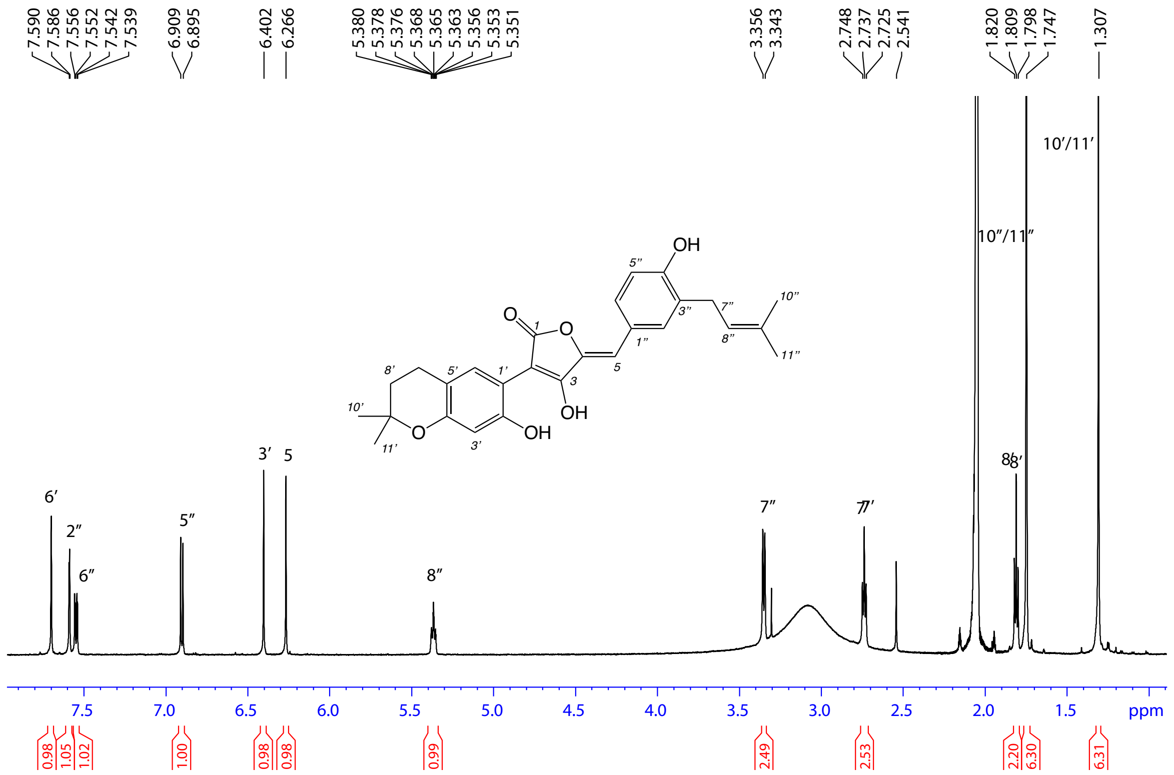

Figure S54. ^{1}H NMR (600 MHz, acetone-d_6) spectrum for aspulvinone D (**17**).

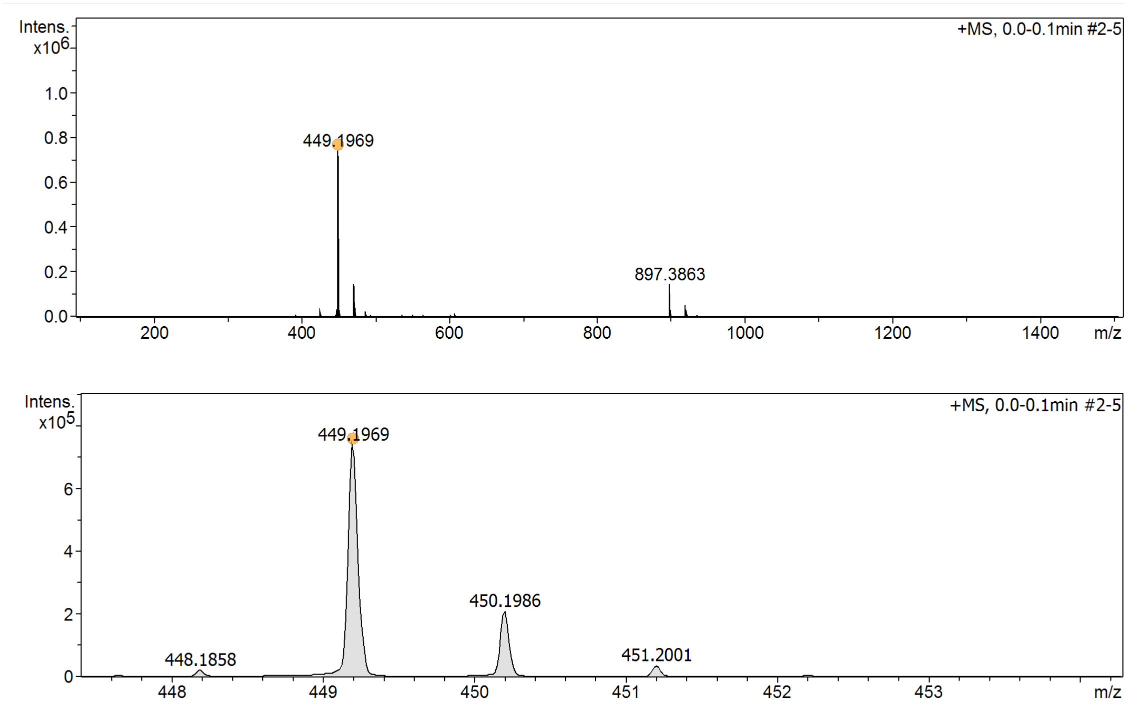

Meas. m/z	#	Ion Formula	m/z	err [ppm]	mSigma	# Sigma	Score	rdb	e¯ Conf	N-Rule
449.1969	1	C23H25N6O4	449.1932	-8.3	4.5	1	9.26	14.5	even	ok
	2	C27H29O6	449.1959	2.3	8.0	2	84.47	13.5	even	ok
	3	C24H21N10	449.1945	-5.3	8.3	3	32.29	19.5	even	ok
	4	C28H25N4O2	449.1972	-0.6	19.2	4	100.00	18.5	even	ok

Figure S55. HRMS spectrum and measurement for aspulvinone D (**17**).

4.12 Aspulvinone H (18)

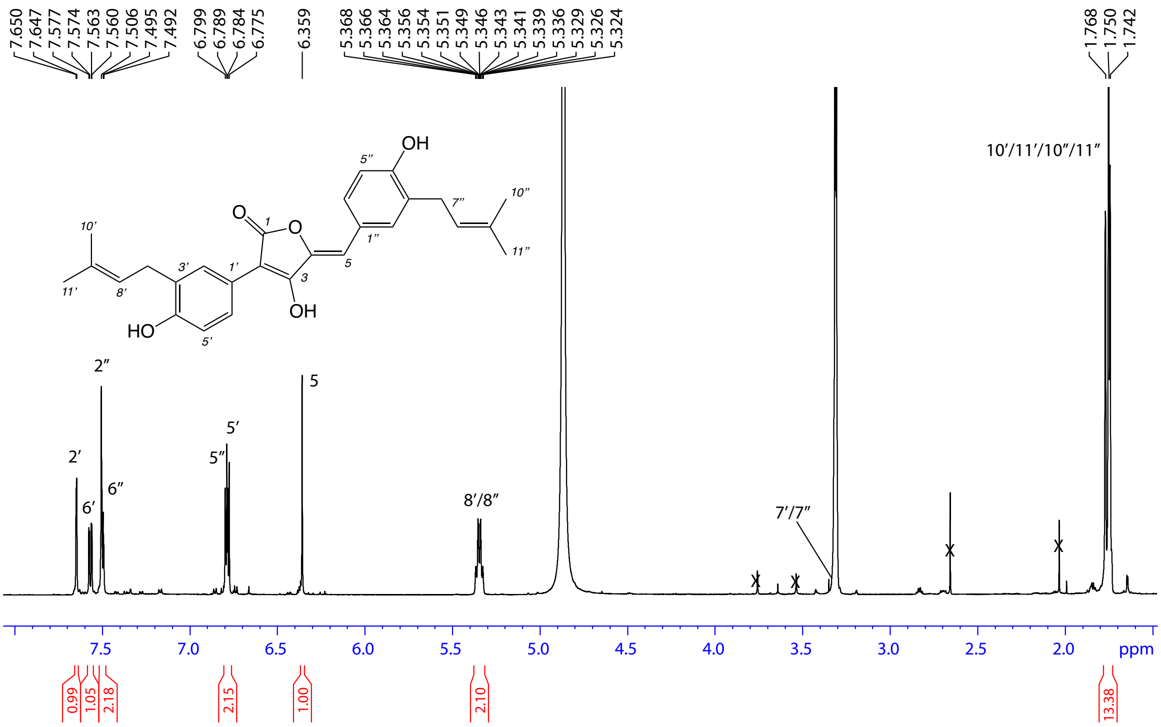

Figure S56. ^{1}H NMR (600 MHz, methanol-d_4) spectrum for aspulvinone H (**18**).

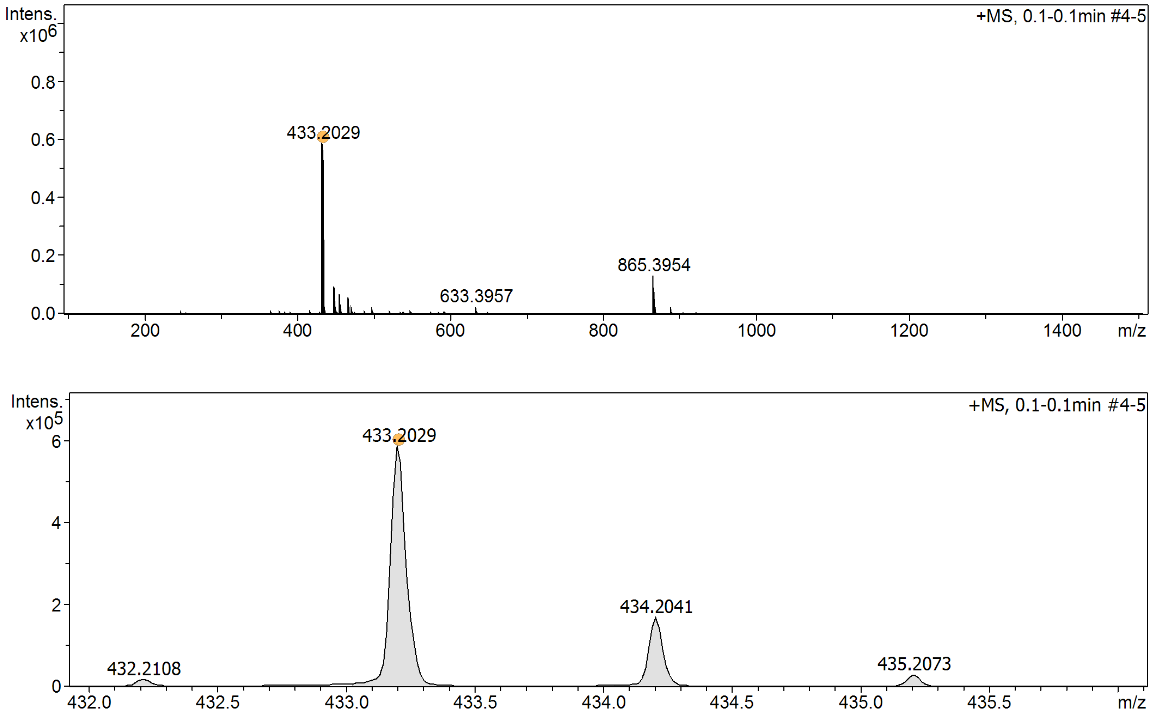

Meas. m/z	#	Ion Formula	m/z	err [ppm]	mSigma	# Sigma	Score	rdb	e¯ Conf	N-Rule
433.2029	1	C27H29O5	433.2010	4.6	6.1	1	53.15	13.5	even	ok
	2	C28H25N4O	433.2023	1.5	17.2	2	100.00	18.5	even	ok
	3	C20H33O10	433.2068	9.0	31.4	3	5.04	4.5	even	ok

Figure S57. HRMS spectrum and measurement for aspulvinone H (**18**).

5. X-ray Crystallography of Aflaquinolone H (11)

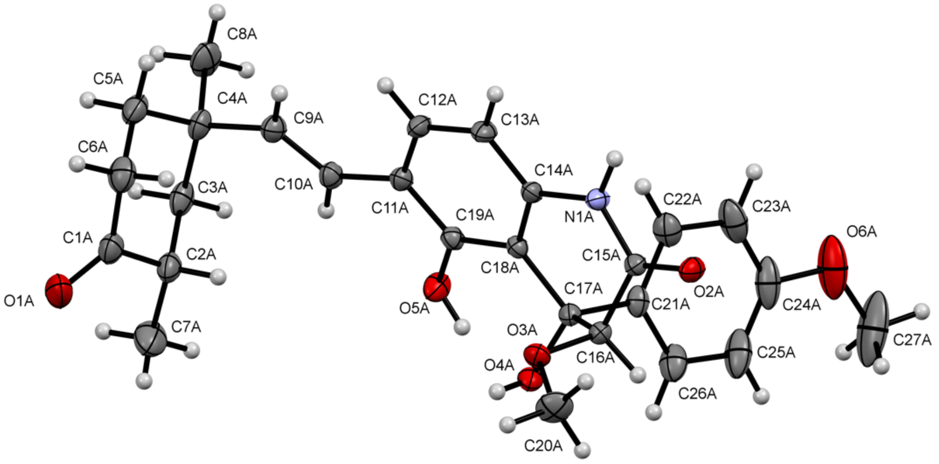

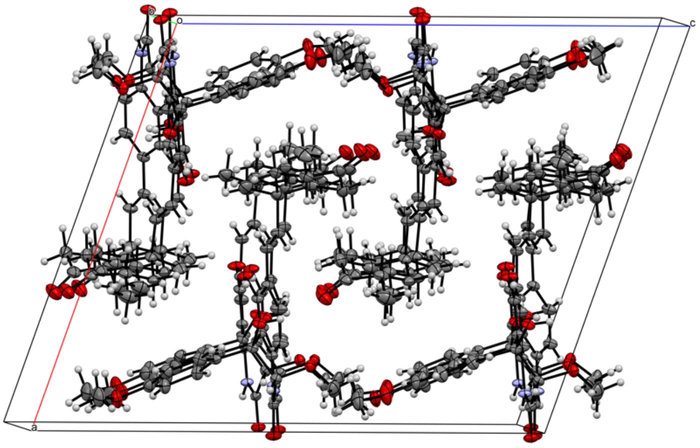

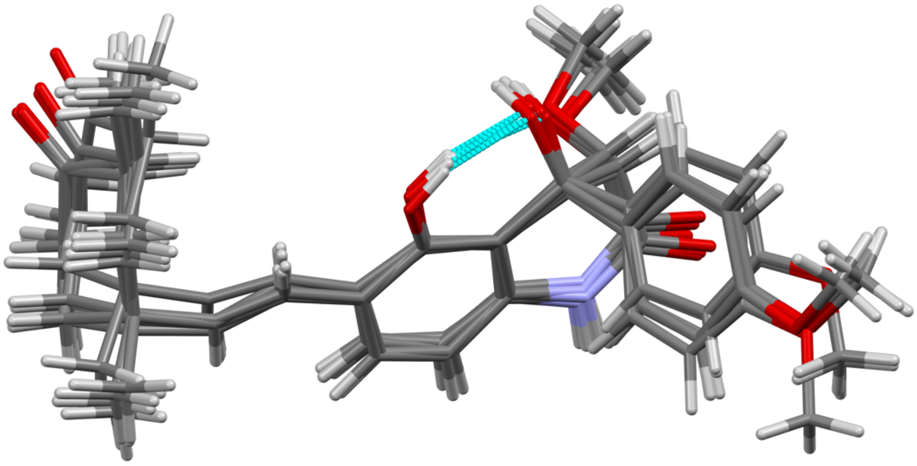

Crystal data and structure refinement for 11.

Identification code	shelx
Empirical formula	$C_{27}H_{31}NO_6$
Formula weight	465.53
Temperature	190(2) K
Wavelength	1.54184 Å

Crystal system	Monoclinic		
Space group	$P2_1$		
Unit cell dimensions	a = 16.8928(5) Å	α= 90°.	
	b = 24.0733(7) Å	β= 109.271(3)°.	
	c = 20.0238(7) Å	γ = 90°.	
Volume	7686.7(4) $Å^3$		
Z	12		
Density (calculated)	1.207 Mg/m^3		
Absorption coefficient	0.694 mm^{-1}		
F(000)	2976		
Crystal size	0.150 x 0.150 x 0.050 mm^3		
Theta range for data collection	3.499 to 62.201°.		
Index ranges	-19<=h<=19, -27<=k<=24, -22<=l<=22		
Reflections collected	86232		
Independent reflections	22470 [R(int) = 0.0662]		
Completeness to theta = 62.201°	98.1 %		
Absorption correction	Semi-empirical from equivalents		
Max. and min. transmission	1 and 0.944		
Refinement method	Full-matrix least-squares on F^2		
Data / restraints / parameters	22470 / 1 / 1873		
Goodness-of-fit on F^2	0.949		
Final R indices [I>2sigma(I)]	R1 = 0.0425, wR2 = 0.0790		
R indices (all data)	R1 = 0.0632, wR2 = 0.0868		
Absolute structure parameter	-0.02(8)		
Extinction coefficient	n/a		
Largest diff. peak and hole	0.144 and -0.169 e.$Å^{-3}$		

Bond lengths [Å] and angles [°] for 11.

C(1A)-O(1A)	1.218(5)	O(1B)-C(1B)-C(6B)	122.1(4)
C(1A)-C(6A)	1.501(7)	O(1B)-C(1B)-C(2B)	121.9(4)
C(1A)-C(2A)	1.512(6)	C(6B)-C(1B)-C(2B)	116.0(4)
C(2A)-C(7A)	1.519(7)	C(1B)-C(2B)-C(7B)	112.1(4)
C(2A)-C(3A)	1.540(7)	C(1B)-C(2B)-C(3B)	110.5(3)
C(3A)-C(4A)	1.535(6)	C(7B)-C(2B)-C(3B)	113.3(4)
C(4A)-C(9A)	1.514(6)	C(4B)-C(3B)-C(2B)	114.4(3)
C(4A)-C(5A)	1.533(6)	C(3B)-C(4B)-C(9B)	112.5(3)
C(4A)-C(8A)	1.535(6)	C(3B)-C(4B)-C(5B)	108.0(3)
C(5A)-C(6A)	1.527(7)	C(9B)-C(4B)-C(5B)	108.9(3)
C(9A)-C(10A)	1.327(6)	C(3B)-C(4B)-C(8B)	110.3(4)
C(10A)-C(11A)	1.472(6)	C(9B)-C(4B)-C(8B)	108.2(3)
C(11A)-C(12A)	1.389(6)	C(5B)-C(4B)-C(8B)	108.8(4)
C(11A)-C(19A)	1.402(5)	C(6B)-C(5B)-C(4B)	112.7(3)
C(12A)-C(13A)	1.378(6)	C(1B)-C(6B)-C(5B)	112.0(4)
C(13A)-C(14A)	1.382(5)	C(10B)-C(9B)-C(4B)	127.7(4)
C(14A)-C(18A)	1.395(5)	C(9B)-C(10B)-C(11B)	125.5(4)
C(14A)-N(1A)	1.421(5)	C(12B)-C(11B)-C(19B)	117.5(3)
C(15A)-O(2A)	1.236(4)	C(12B)-C(11B)-C(10B)	122.3(3)
C(15A)-N(1A)	1.344(5)	C(19B)-C(11B)-C(10B)	120.2(3)
C(15A)-C(16A)	1.513(5)	C(13B)-C(12B)-C(11B)	122.0(3)
C(16A)-O(3A)	1.419(4)	C(14B)-C(13B)-C(12B)	119.1(3)
C(16A)-C(17A)	1.536(5)	C(13B)-C(14B)-C(18B)	121.5(3)
C(17A)-O(4A)	1.424(4)	C(13B)-C(14B)-N(1B)	119.5(3)
C(17A)-C(18A)	1.521(5)	C(18B)-C(14B)-N(1B)	119.0(3)
C(17A)-C(21A)	1.527(5)	O(2B)-C(15B)-N(1B)	122.6(3)
C(18A)-C(19A)	1.396(5)	O(2B)-C(15B)-C(16B)	121.3(3)
C(19A)-O(5A)	1.367(5)	N(1B)-C(15B)-C(16B)	115.9(3)
C(20A)-O(3A)	1.420(5)	O(3B)-C(16B)-C(15B)	107.2(3)
C(21A)-C(26A)	1.383(6)	O(3B)-C(16B)-C(17B)	108.2(3)
C(21A)-C(22A)	1.388(6)	C(15B)-C(16B)-C(17B)	112.3(3)
C(22A)-C(23A)	1.394(7)	O(4B)-C(17B)-C(18B)	110.9(3)
C(23A)-C(24A)	1.374(9)	O(4B)-C(17B)-C(21B)	107.1(3)

C(24A)-C(25A)	1.369(9)	C(18B)-C(17B)-C(21B)	114.2(3)
C(24A)-O(6A)	1.382(6)	O(4B)-C(17B)-C(16B)	107.6(3)
C(25A)-C(26A)	1.393(7)	C(18B)-C(17B)-C(16B)	109.2(3)
C(27A)-O(6A)	1.399(10)	C(21B)-C(17B)-C(16B)	107.6(3)
C(1B)-O(1B)	1.226(5)	C(14B)-C(18B)-C(19B)	118.1(3)
C(1B)-C(6B)	1.496(7)	C(14B)-C(18B)-C(17B)	119.1(3)
C(1B)-C(2B)	1.508(6)	C(19B)-C(18B)-C(17B)	122.8(3)
C(2B)-C(7B)	1.522(6)	O(5B)-C(19B)-C(18B)	121.9(3)
C(2B)-C(3B)	1.531(6)	O(5B)-C(19B)-C(11B)	116.4(3)
C(3B)-C(4B)	1.522(6)	C(18B)-C(19B)-C(11B)	121.7(3)
C(4B)-C(9B)	1.521(5)	C(26B)-C(21B)-C(22B)	118.3(4)
C(4B)-C(5B)	1.534(6)	C(26B)-C(21B)-C(17B)	122.2(3)
C(4B)-C(8B)	1.537(6)	C(22B)-C(21B)-C(17B)	119.3(3)
C(5B)-C(6B)	1.522(7)	C(23B)-C(22B)-C(21B)	119.9(4)
C(9B)-C(10B)	1.323(5)	C(22B)-C(23B)-C(24B)	121.5(4)
C(10B)-C(11B)	1.473(5)	O(6B)-C(24B)-C(25B)	124.3(4)
C(11B)-C(12B)	1.395(5)	O(6B)-C(24B)-C(23B)	116.3(4)
C(11B)-C(19B)	1.402(5)	C(25B)-C(24B)-C(23B)	119.4(4)
C(12B)-C(13B)	1.383(5)	C(24B)-C(25B)-C(26B)	119.6(4)
C(13B)-C(14B)	1.379(5)	C(21B)-C(26B)-C(25B)	121.1(4)
C(14B)-C(18B)	1.397(5)	C(15B)-N(1B)-C(14B)	124.3(3)
C(14B)-N(1B)	1.422(5)	C(20B)-O(3B)-C(16B)	113.8(3)
C(15B)-O(2B)	1.236(4)	C(24B)-O(6B)-C(27B)	118.6(4)
C(15B)-N(1B)	1.340(5)	O(1C)-C(1C)-C(6C)	122.8(4)
C(15B)-C(16B)	1.521(5)	O(1C)-C(1C)-C(2C)	121.0(4)
C(16B)-O(3B)	1.426(4)	C(6C)-C(1C)-C(2C)	116.2(4)
C(16B)-C(17B)	1.528(5)	C(1C)-C(2C)-C(7C)	113.0(4)
C(17B)-O(4B)	1.433(4)	C(1C)-C(2C)-C(3C)	110.4(4)
C(17B)-C(18B)	1.521(5)	C(7C)-C(2C)-C(3C)	112.8(4)
C(17B)-C(21B)	1.526(5)	C(4C)-C(3C)-C(2C)	113.8(3)
C(18B)-C(19B)	1.399(5)	C(9C)-C(4C)-C(3C)	112.7(3)
C(19B)-O(5B)	1.365(4)	C(9C)-C(4C)-C(5C)	109.6(3)
C(20B)-O(3B)	1.428(5)	C(3C)-C(4C)-C(5C)	107.9(3)
C(21B)-C(26B)	1.385(6)	C(9C)-C(4C)-C(8C)	107.9(3)
C(21B)-C(22B)	1.395(6)	C(3C)-C(4C)-C(8C)	109.5(4)
C(22B)-C(23B)	1.373(6)	C(5C)-C(4C)-C(8C)	109.2(4)
C(23B)-C(24B)	1.376(7)	C(6C)-C(5C)-C(4C)	112.3(3)
C(24B)-O(6B)	1.372(5)	C(1C)-C(6C)-C(5C)	111.4(4)
C(24B)-C(25B)	1.371(6)	C(10C)-C(9C)-C(4C)	127.9(4)
C(25B)-C(26B)	1.396(6)	C(9C)-C(10C)-C(11C)	126.0(4)
C(27B)-O(6B)	1.422(7)	C(12C)-C(11C)-C(19C)	117.5(3)
C(1C)-O(1C)	1.221(5)	C(12C)-C(11C)-C(10C)	122.2(4)
C(1C)-C(6C)	1.490(7)	C(19C)-C(11C)-C(10C)	120.3(3)
C(1C)-C(2C)	1.503(6)	C(13C)-C(12C)-C(11C)	122.3(4)
C(2C)-C(7C)	1.519(7)	C(12C)-C(13C)-C(14C)	119.3(4)
C(2C)-C(3C)	1.536(6)	C(13C)-C(14C)-C(18C)	121.1(3)
C(3C)-C(4C)	1.528(6)	C(13C)-C(14C)-N(1C)	119.6(3)
C(4C)-C(9C)	1.509(6)	C(18C)-C(14C)-N(1C)	119.3(3)
C(4C)-C(5C)	1.535(6)	O(5C)-C(15C)-N(1C)	122.8(3)
C(4C)-C(8C)	1.540(6)	O(5C)-C(15C)-C(16C)	122.1(3)
C(5C)-C(6C)	1.527(7)	N(1C)-C(15C)-C(16C)	115.0(3)
C(9C)-C(10C)	1.323(5)	O(4C)-C(16C)-C(15C)	107.9(3)
C(10C)-C(11C)	1.468(5)	O(4C)-C(16C)-C(17C)	107.1(3)
C(11C)-C(12C)	1.387(6)	C(15C)-C(16C)-C(17C)	111.6(3)
C(11C)-C(19C)	1.403(5)	O(3C)-C(17C)-C(21C)	107.8(3)
C(12C)-C(13C)	1.377(6)	O(3C)-C(17C)-C(18C)	110.9(3)
C(13C)-C(14C)	1.381(5)	C(21C)-C(17C)-C(18C)	113.5(3)
C(14C)-C(18C)	1.399(5)	O(3C)-C(17C)-C(16C)	107.8(3)
C(14C)-N(1C)	1.414(5)	C(21C)-C(17C)-C(16C)	108.4(3)
C(15C)-O(5C)	1.237(4)	C(18C)-C(17C)-C(16C)	108.2(3)
C(15C)-N(1C)	1.339(5)	C(14C)-C(18C)-C(19C)	118.1(3)
C(15C)-C(16C)	1.511(5)	C(14C)-C(18C)-C(17C)	118.8(3)
C(16C)-O(4C)	1.429(4)	C(19C)-C(18C)-C(17C)	123.1(3)

C(16C)-C(17C)	1.540(5)	O(2C)-C(19C)-C(18C)	121.4(3)
C(17C)-O(3C)	1.430(4)	O(2C)-C(19C)-C(11C)	117.0(3)
C(17C)-C(21C)	1.519(5)	C(18C)-C(19C)-C(11C)	121.6(3)
C(17C)-C(18C)	1.520(5)	C(22C)-C(21C)-C(26C)	117.8(3)
C(18C)-C(19C)	1.400(5)	C(22C)-C(21C)-C(17C)	120.6(3)
C(19C)-O(2C)	1.365(4)	C(26C)-C(21C)-C(17C)	121.5(3)
C(20C)-O(4C)	1.424(5)	C(21C)-C(22C)-C(23C)	121.5(4)
C(21C)-C(22C)	1.384(5)	C(24C)-C(23C)-C(22C)	119.4(4)
C(21C)-C(26C)	1.389(6)	O(6C)-C(24C)-C(25C)	115.8(4)
C(22C)-C(23C)	1.398(6)	O(6C)-C(24C)-C(23C)	124.6(4)
C(23C)-C(24C)	1.382(6)	C(25C)-C(24C)-C(23C)	119.6(4)
C(24C)-O(6C)	1.369(5)	C(26C)-C(25C)-C(24C)	120.3(4)
C(24C)-C(25C)	1.385(6)	C(25C)-C(26C)-C(21C)	121.3(4)
C(25C)-C(26C)	1.378(6)	C(15C)-N(1C)-C(14C)	124.0(3)
C(27C)-O(6C)	1.428(6)	C(20C)-O(4C)-C(16C)	113.5(3)
C(1D)-O(1D)	1.217(5)	C(24C)-O(6C)-C(27C)	117.8(4)
C(1D)-C(6D)	1.497(7)	O(1D)-C(1D)-C(6D)	122.3(4)
C(1D)-C(2D)	1.509(6)	O(1D)-C(1D)-C(2D)	121.6(4)
C(2D)-C(7D)	1.510(7)	C(6D)-C(1D)-C(2D)	116.1(4)
C(2D)-C(3D)	1.532(6)	C(7D)-C(2D)-C(1D)	113.0(4)
C(3D)-C(4D)	1.531(6)	C(7D)-C(2D)-C(3D)	113.7(4)
C(4D)-C(9D)	1.516(5)	C(1D)-C(2D)-C(3D)	109.7(3)
C(4D)-C(5D)	1.536(6)	C(4D)-C(3D)-C(2D)	114.1(3)
C(4D)-C(8D)	1.535(6)	C(9D)-C(4D)-C(3D)	111.8(3)
C(5D)-C(6D)	1.525(6)	C(9D)-C(4D)-C(5D)	109.3(3)
C(9D)-C(10D)	1.326(5)	C(3D)-C(4D)-C(5D)	107.5(3)
C(10D)-C(11D)	1.466(5)	C(9D)-C(4D)-C(8D)	109.0(3)
C(11D)-C(12D)	1.389(5)	C(3D)-C(4D)-C(8D)	110.5(3)
C(11D)-C(19D)	1.401(5)	C(5D)-C(4D)-C(8D)	108.7(3)
C(12D)-C(13D)	1.378(6)	C(6D)-C(5D)-C(4D)	112.8(3)
C(13D)-C(14D)	1.383(5)	C(1D)-C(6D)-C(5D)	111.5(4)
C(14D)-C(18D)	1.396(5)	C(10D)-C(9D)-C(4D)	127.5(4)
C(14D)-N(1D)	1.415(5)	C(9D)-C(10D)-C(11D)	125.8(4)
C(15D)-O(2D)	1.228(4)	C(12D)-C(11D)-C(19D)	117.0(3)
C(15D)-N(1D)	1.345(5)	C(12D)-C(11D)-C(10D)	122.6(3)
C(15D)-C(16D)	1.518(5)	C(19D)-C(11D)-C(10D)	120.4(3)
C(16D)-O(3D)	1.423(4)	C(13D)-C(12D)-C(11D)	122.6(4)
C(16D)-C(17D)	1.534(5)	C(12D)-C(13D)-C(14D)	119.0(3)
C(17D)-O(4D)	1.431(4)	C(13D)-C(14D)-C(18D)	121.1(3)
C(17D)-C(18D)	1.520(5)	C(13D)-C(14D)-N(1D)	119.1(3)
C(17D)-C(21D)	1.524(5)	C(18D)-C(14D)-N(1D)	119.7(3)
C(18D)-C(19D)	1.395(5)	O(2D)-C(15D)-N(1D)	122.8(3)
C(19D)-O(6D)	1.365(4)	O(2D)-C(15D)-C(16D)	122.1(3)
C(20D)-O(3D)	1.418(5)	N(1D)-C(15D)-C(16D)	114.9(3)
C(21D)-C(26D)	1.382(5)	O(3D)-C(16D)-C(15D)	107.7(3)
C(21D)-C(22D)	1.392(6)	O(3D)-C(16D)-C(17D)	108.5(3)
C(22D)-C(23D)	1.379(6)	C(15D)-C(16D)-C(17D)	112.3(3)
C(23D)-C(24D)	1.386(6)	O(4D)-C(17D)-C(18D)	111.4(3)
C(24D)-C(25D)	1.373(6)	O(4D)-C(17D)-C(21D)	107.2(3)
C(24D)-O(5D)	1.375(5)	C(18D)-C(17D)-C(21D)	113.5(3)
C(25D)-C(26D)	1.388(6)	O(4D)-C(17D)-C(16D)	107.7(3)
C(27D)-O(5D)	1.422(6)	C(18D)-C(17D)-C(16D)	108.8(3)
C(1E)-O(1E)	1.225(6)	C(21D)-C(17D)-C(16D)	108.0(3)
C(1E)-C(6E)	1.496(7)	C(14D)-C(18D)-C(19D)	118.2(3)
C(1E)-C(2E)	1.509(6)	C(14D)-C(18D)-C(17D)	118.7(3)
C(2E)-C(7E)	1.514(7)	C(19D)-C(18D)-C(17D)	123.1(3)
C(2E)-C(3E)	1.535(6)	O(6D)-C(19D)-C(18D)	121.5(3)
C(3E)-C(4E)	1.533(7)	O(6D)-C(19D)-C(11D)	116.5(3)
C(4E)-C(9E)	1.517(6)	C(18D)-C(19D)-C(11D)	122.0(3)
C(4E)-C(8E)	1.522(6)	C(26D)-C(21D)-C(22D)	117.9(3)
C(4E)-C(5E)	1.548(6)	C(26D)-C(21D)-C(17D)	122.6(3)
C(5E)-C(6E)	1.518(7)	C(22D)-C(21D)-C(17D)	119.4(3)
C(9E)-C(10E)	1.318(6)	C(23D)-C(22D)-C(21D)	120.6(4)

C(10E)-C(11E)	1.471(6)	C(22D)-C(23D)-C(24D)	120.5(4)
C(11E)-C(12E)	1.394(6)	C(25D)-C(24D)-O(5D)	124.8(4)
C(11E)-C(19E)	1.406(5)	C(25D)-C(24D)-C(23D)	119.9(4)
C(12E)-C(13E)	1.375(6)	O(5D)-C(24D)-C(23D)	115.3(4)
C(13E)-C(14E)	1.393(5)	C(24D)-C(25D)-C(26D)	119.1(4)
C(14E)-C(18E)	1.386(5)	C(21D)-C(26D)-C(25D)	122.1(4)
C(14E)-N(1E)	1.421(5)	C(15D)-N(1D)-C(14D)	124.3(3)
C(15E)-O(5E)	1.232(4)	C(20D)-O(3D)-C(16D)	114.4(3)
C(15E)-N(1E)	1.342(5)	C(24D)-O(5D)-C(27D)	118.0(4)
C(15E)-C(16E)	1.521(5)	O(1E)-C(1E)-C(6E)	123.1(4)
C(16E)-O(4E)	1.423(4)	O(1E)-C(1E)-C(2E)	120.8(4)
C(16E)-C(17E)	1.535(5)	C(6E)-C(1E)-C(2E)	116.1(4)
C(17E)-O(3E)	1.437(4)	C(1E)-C(2E)-C(7E)	112.9(4)
C(17E)-C(21E)	1.513(5)	C(1E)-C(2E)-C(3E)	109.6(4)
C(17E)-C(18E)	1.523(5)	C(7E)-C(2E)-C(3E)	113.5(4)
C(18E)-C(19E)	1.397(5)	C(2E)-C(3E)-C(4E)	114.8(3)
C(19E)-O(2E)	1.365(4)	C(9E)-C(4E)-C(8E)	108.7(4)
C(20E)-O(4E)	1.421(5)	C(9E)-C(4E)-C(3E)	112.2(3)
C(21E)-C(26E)	1.383(5)	C(8E)-C(4E)-C(3E)	109.9(4)
C(21E)-C(22E)	1.392(6)	C(9E)-C(4E)-C(5E)	109.1(4)
C(22E)-C(23E)	1.382(6)	C(8E)-C(4E)-C(5E)	109.6(4)
C(23E)-C(24E)	1.381(6)	C(3E)-C(4E)-C(5E)	107.3(4)
C(24E)-O(6E)	1.370(5)	C(6E)-C(5E)-C(4E)	112.7(4)
C(24E)-C(25E)	1.374(6)	C(1E)-C(6E)-C(5E)	112.1(4)
C(25E)-C(26E)	1.399(6)	C(10E)-C(9E)-C(4E)	127.2(4)
C(27E)-O(6E)	1.430(6)	C(9E)-C(10E)-C(11E)	126.6(4)
C(1F)-O(1F)	1.224(5)	C(12E)-C(11E)-C(19E)	117.5(3)
C(1F)-C(2F)	1.500(6)	C(12E)-C(11E)-C(10E)	122.7(4)
C(1F)-C(6F)	1.499(7)	C(19E)-C(11E)-C(10E)	119.8(3)
C(2F)-C(7F)	1.514(6)	C(13E)-C(12E)-C(11E)	122.2(4)
C(2F)-C(3F)	1.533(6)	C(12E)-C(13E)-C(14E)	119.0(4)
C(3F)-C(4F)	1.539(6)	C(18E)-C(14E)-C(13E)	121.2(3)
C(4F)-C(9F)	1.514(6)	C(18E)-C(14E)-N(1E)	119.6(3)
C(4F)-C(5F)	1.532(6)	C(13E)-C(14E)-N(1E)	119.2(3)
C(4F)-C(8F)	1.543(6)	O(5E)-C(15E)-N(1E)	123.0(3)
C(5F)-C(6F)	1.522(6)	O(5E)-C(15E)-C(16E)	122.5(3)
C(9F)-C(10F)	1.329(6)	N(1E)-C(15E)-C(16E)	114.4(3)
C(10F)-C(11F)	1.473(5)	O(4E)-C(16E)-C(15E)	108.0(3)
C(11F)-C(12F)	1.386(5)	O(4E)-C(16E)-C(17E)	106.8(3)
C(11F)-C(19F)	1.406(5)	C(15E)-C(16E)-C(17E)	111.7(3)
C(12F)-C(13F)	1.386(5)	O(3E)-C(17E)-C(21E)	107.7(3)
C(13F)-C(14F)	1.381(5)	O(3E)-C(17E)-C(18E)	110.4(3)
C(14F)-C(18F)	1.386(5)	C(21E)-C(17E)-C(18E)	114.1(3)
C(14F)-N(1F)	1.420(5)	O(3E)-C(17E)-C(16E)	107.4(3)
C(15F)-O(5F)	1.237(4)	C(21E)-C(17E)-C(16E)	108.8(3)
C(15F)-N(1F)	1.341(5)	C(18E)-C(17E)-C(16E)	108.3(3)
C(15F)-C(16F)	1.514(5)	C(14E)-C(18E)-C(19E)	118.5(3)
C(16F)-O(4F)	1.427(4)	C(14E)-C(18E)-C(17E)	118.6(3)
C(16F)-C(17F)	1.536(5)	C(19E)-C(18E)-C(17E)	122.8(3)
C(17F)-O(3F)	1.431(4)	O(2E)-C(19E)-C(18E)	121.7(3)
C(17F)-C(21F)	1.515(5)	O(2E)-C(19E)-C(11E)	116.8(3)
C(17F)-C(18F)	1.521(5)	C(18E)-C(19E)-C(11E)	121.5(3)
C(18F)-C(19F)	1.408(5)	C(26E)-C(21E)-C(22E)	118.0(3)
C(19F)-O(2F)	1.362(4)	C(26E)-C(21E)-C(17E)	120.2(3)
C(20F)-O(4F)	1.425(5)	C(22E)-C(21E)-C(17E)	121.7(3)
C(21F)-C(26F)	1.383(6)	C(23E)-C(22E)-C(21E)	121.3(4)
C(21F)-C(22F)	1.393(6)	C(22E)-C(23E)-C(24E)	119.8(4)
C(22F)-C(23F)	1.371(6)	O(6E)-C(24E)-C(25E)	124.4(4)
C(23F)-C(24F)	1.379(7)	O(6E)-C(24E)-C(23E)	115.3(4)
C(24F)-O(6F)	1.375(5)	C(25E)-C(24E)-C(23E)	120.3(4)
C(24F)-C(25F)	1.382(7)	C(24E)-C(25E)-C(26E)	119.5(4)
C(25F)-C(26F)	1.394(6)	C(21E)-C(26E)-C(25E)	121.2(4)
C(27F)-O(6F)	1.420(7)	C(15E)-N(1E)-C(14E)	123.6(3)

		C(20E)-O(4E)-C(16E)	114.0(3)
		C(24E)-O(6E)-C(27E)	117.5(4)
O(1A)-C(1A)-C(6A)	122.7(4)	O(1F)-C(1F)-C(2F)	121.8(4)
O(1A)-C(1A)-C(2A)	121.3(4)	O(1F)-C(1F)-C(6F)	122.1(4)
C(6A)-C(1A)-C(2A)	116.0(4)	C(2F)-C(1F)-C(6F)	116.1(4)
C(1A)-C(2A)-C(7A)	113.0(4)	C(1F)-C(2F)-C(7F)	112.8(4)
C(1A)-C(2A)-C(3A)	109.9(4)	C(1F)-C(2F)-C(3F)	110.8(3)
C(7A)-C(2A)-C(3A)	112.4(4)	C(7F)-C(2F)-C(3F)	113.0(4)
C(4A)-C(3A)-C(2A)	114.3(3)	C(2F)-C(3F)-C(4F)	114.5(3)
C(9A)-C(4A)-C(5A)	109.1(3)	C(9F)-C(4F)-C(5F)	109.1(3)
C(9A)-C(4A)-C(3A)	112.5(3)	C(9F)-C(4F)-C(3F)	112.6(3)
C(5A)-C(4A)-C(3A)	108.0(3)	C(5F)-C(4F)-C(3F)	107.7(3)
C(9A)-C(4A)-C(8A)	107.7(4)	C(9F)-C(4F)-C(8F)	108.2(3)
C(5A)-C(4A)-C(8A)	109.7(4)	C(5F)-C(4F)-C(8F)	109.5(3)
C(3A)-C(4A)-C(8A)	109.9(4)	C(3F)-C(4F)-C(8F)	109.7(4)
C(6A)-C(5A)-C(4A)	112.6(4)	C(6F)-C(5F)-C(4F)	112.5(3)
C(1A)-C(6A)-C(5A)	111.8(4)	C(1F)-C(6F)-C(5F)	112.1(4)
C(10A)-C(9A)-C(4A)	127.5(4)	C(10F)-C(9F)-C(4F)	127.8(4)
C(9A)-C(10A)-C(11A)	126.2(4)	C(9F)-C(10F)-C(11F)	125.0(4)
C(12A)-C(11A)-C(19A)	117.4(3)	C(12F)-C(11F)-C(19F)	117.8(3)
C(12A)-C(11A)-C(10A)	122.6(4)	C(12F)-C(11F)-C(10F)	122.4(3)
C(19A)-C(11A)-C(10A)	119.9(4)	C(19F)-C(11F)-C(10F)	119.8(3)
C(13A)-C(12A)-C(11A)	122.3(4)	C(11F)-C(12F)-C(13F)	122.1(3)
C(12A)-C(13A)-C(14A)	119.0(4)	C(14F)-C(13F)-C(12F)	118.8(3)
C(13A)-C(14A)-C(18A)	121.4(3)	C(13F)-C(14F)-C(18F)	121.8(3)
C(13A)-C(14A)-N(1A)	119.4(3)	C(13F)-C(14F)-N(1F)	118.8(3)
C(18A)-C(14A)-N(1A)	119.2(3)	C(18F)-C(14F)-N(1F)	119.4(3)
O(2A)-C(15A)-N(1A)	122.6(3)	O(5F)-C(15F)-N(1F)	122.5(3)
O(2A)-C(15A)-C(16A)	122.4(3)	O(5F)-C(15F)-C(16F)	121.6(3)
N(1A)-C(15A)-C(16A)	114.9(3)	N(1F)-C(15F)-C(16F)	115.7(3)
O(3A)-C(16A)-C(15A)	107.8(3)	O(4F)-C(16F)-C(15F)	107.7(3)
O(3A)-C(16A)-C(17A)	107.0(3)	O(4F)-C(16F)-C(17F)	107.5(3)
C(15A)-C(16A)-C(17A)	112.3(3)	C(15F)-C(16F)-C(17F)	112.4(3)
O(4A)-C(17A)-C(18A)	110.6(3)	O(3F)-C(17F)-C(21F)	107.0(3)
O(4A)-C(17A)-C(21A)	108.0(3)	O(3F)-C(17F)-C(18F)	111.3(3)
C(18A)-C(17A)-C(21A)	113.9(3)	C(21F)-C(17F)-C(18F)	115.0(3)
O(4A)-C(17A)-C(16A)	107.9(3)	O(3F)-C(17F)-C(16F)	107.6(3)
C(18A)-C(17A)-C(16A)	108.0(3)	C(21F)-C(17F)-C(16F)	107.5(3)
C(21A)-C(17A)-C(16A)	108.4(3)	C(18F)-C(17F)-C(16F)	108.1(3)
C(14A)-C(18A)-C(19A)	118.1(3)	C(14F)-C(18F)-C(19F)	118.2(3)
C(14A)-C(18A)-C(17A)	118.8(3)	C(14F)-C(18F)-C(17F)	119.1(3)
C(19A)-C(18A)-C(17A)	123.0(3)	C(19F)-C(18F)-C(17F)	122.7(3)
O(5A)-C(19A)-C(18A)	121.5(3)	O(2F)-C(19F)-C(18F)	121.9(3)
O(5A)-C(19A)-C(11A)	116.8(3)	O(2F)-C(19F)-C(11F)	117.0(3)
C(18A)-C(19A)-C(11A)	121.7(3)	C(18F)-C(19F)-C(11F)	121.1(3)
C(26A)-C(21A)-C(22A)	118.6(4)	C(26F)-C(21F)-C(22F)	117.8(4)
C(26A)-C(21A)-C(17A)	119.2(4)	C(26F)-C(21F)-C(17F)	122.8(3)
C(22A)-C(21A)-C(17A)	122.1(4)	C(22F)-C(21F)-C(17F)	119.3(3)
C(21A)-C(22A)-C(23A)	120.2(5)	C(23F)-C(22F)-C(21F)	121.1(4)
C(24A)-C(23A)-C(22A)	120.1(5)	C(22F)-C(23F)-C(24F)	120.4(4)
C(25A)-C(24A)-C(23A)	120.4(5)	O(6F)-C(24F)-C(25F)	124.1(5)
C(25A)-C(24A)-O(6A)	124.7(7)	O(6F)-C(24F)-C(23F)	115.9(4)
C(23A)-C(24A)-O(6A)	115.0(7)	C(25F)-C(24F)-C(23F)	120.0(4)
C(24A)-C(25A)-C(26A)	119.6(5)	C(24F)-C(25F)-C(26F)	119.0(4)
C(21A)-C(26A)-C(25A)	121.0(5)	C(21F)-C(26F)-C(25F)	121.6(4)
C(15A)-N(1A)-C(14A)	123.8(3)	C(15F)-N(1F)-C(14F)	123.6(3)
C(16A)-O(3A)-C(20A)	114.6(3)	C(16F)-O(4F)-C(20F)	114.6(3)
C(24A)-O(6A)-C(27A)	117.3(7)	C(24F)-O(6F)-C(27F)	118.3(4)

6. MS/MS fragmentation analysis for aspergillamides related nodes

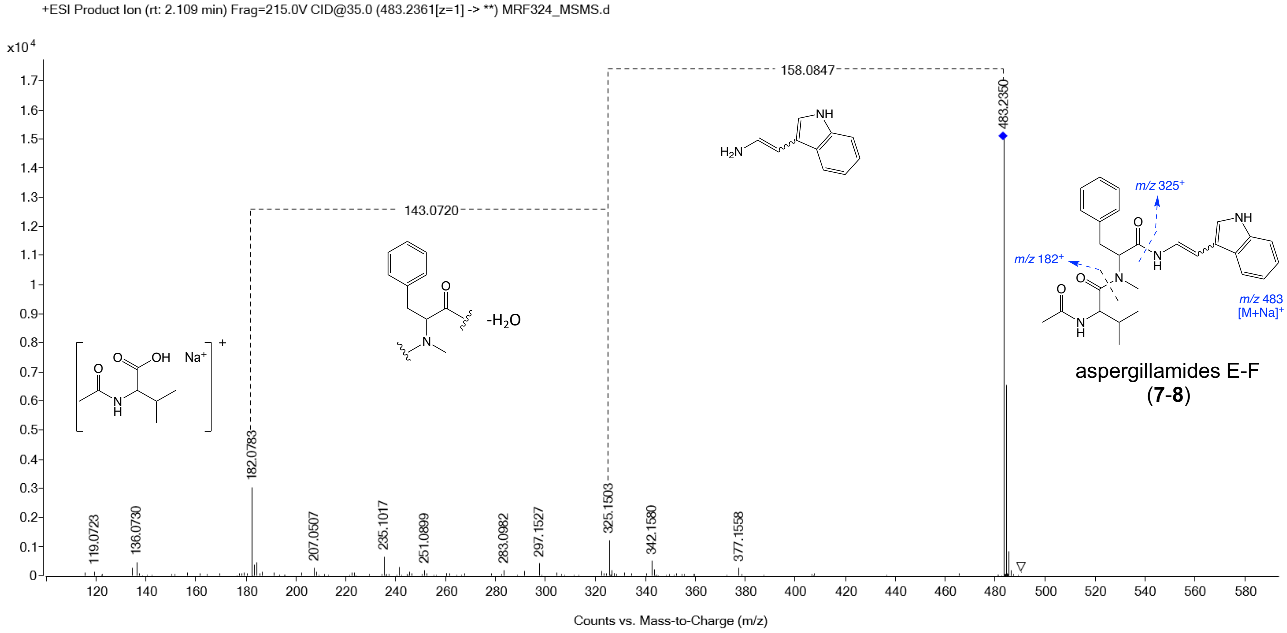

○	MFG		C27 H32 N4 O3	(M+Na)+	483.2360	98.15			-1.35			98.15

Species	m/z	Score (iso. abund)	Score (mass)	Score (MFG, MS/MS)	Score (MS)	Score (MFG)	Score (iso. spacing)	Height	Ion Formula	Lib/DB	RT	RT Diff
(M+Na)+	483.236	97.1	98.28		98.15	98.15	99.16	229558.6	C27 H32 N4 Na O3			

Height (Calc)	Height Sum% (Calc)	Height % (Calc)	m/z (Calc)	Diff (mDa)	Height	Height %	Height Sum %	m/z	Diff (ppm)
224296.9	73.29	100	483.2367	-0.6	229558.6	100	75	483.236	-1.31
69859.8	22.83	31.15	484.2398	-0.4	67961.6	29.61	22.21	484.2394	-0.75
11901.8	3.89	5.31	485.2426	-2.2	8538.2	3.72	2.79	485.2404	-4.6

Figure S58. MS/MS fragmentation and HRMS analysis for aspergillamides E-F (**7-8**).

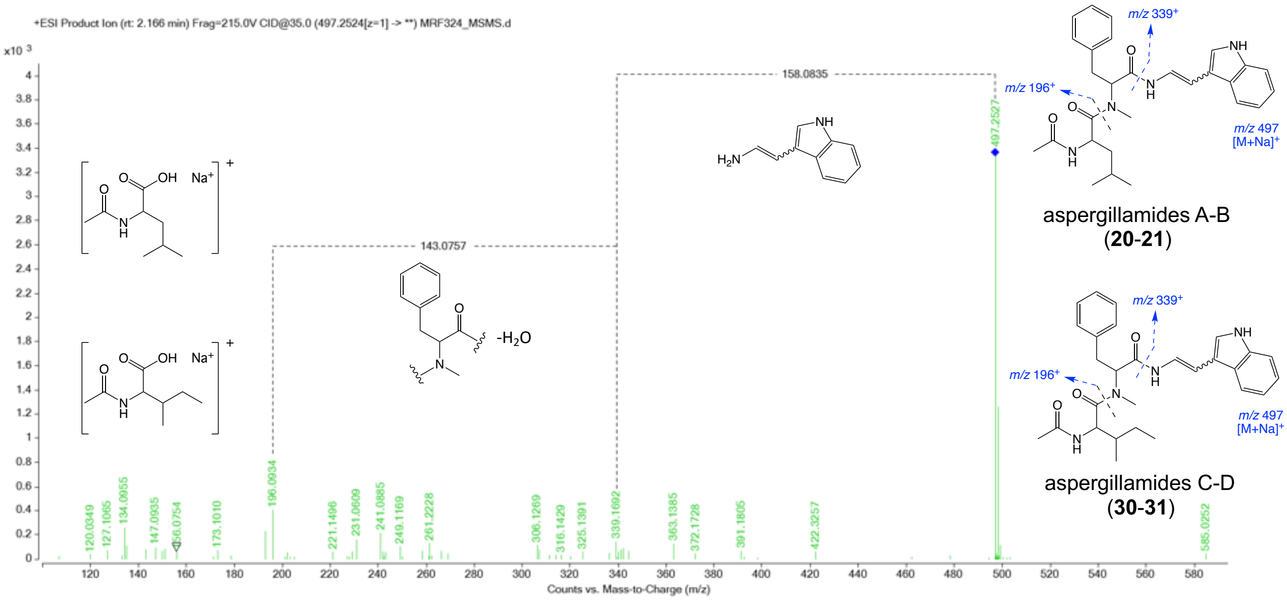

○	MFG		C28 H34 N4 O3	(M+Na)+	497.2527	98.86			0.36			98.86

Species	m/z	Score (iso. abund)	Score (mass)	Score (MFG, MS/MS)	Score (MS)	Score (MFG)	Score (iso. spacing)	Height	Ion Formula	Lib/DB	RT	RT Diff
(M+Na)+	497.2527	98.56	99.87		98.86	98.86	97.17	708312.1	C28 H34 N4 Na O3			

Height (Calc)	Height Sum% (Calc)	Height % (Calc)	m/z (Calc)	Diff (mDa)	Height	Height %	Height Sum %	m/z	Diff (ppm)
700621	72.52	100	497.2523	0.4	708312.1	100	73.31	497.2527	0.8
225955	23.39	32.25	498.2554	-0.5	225614.9	31.85	23.35	498.2549	-0.99
39588.9	4.1	5.65	499.2583	-0.1	32238	4.55	3.34	499.2582	-0.28

Figure S59. MS/MS fragmentation and HRMS analysis for aspergillamides A-D (**20-21**, **30-31**).

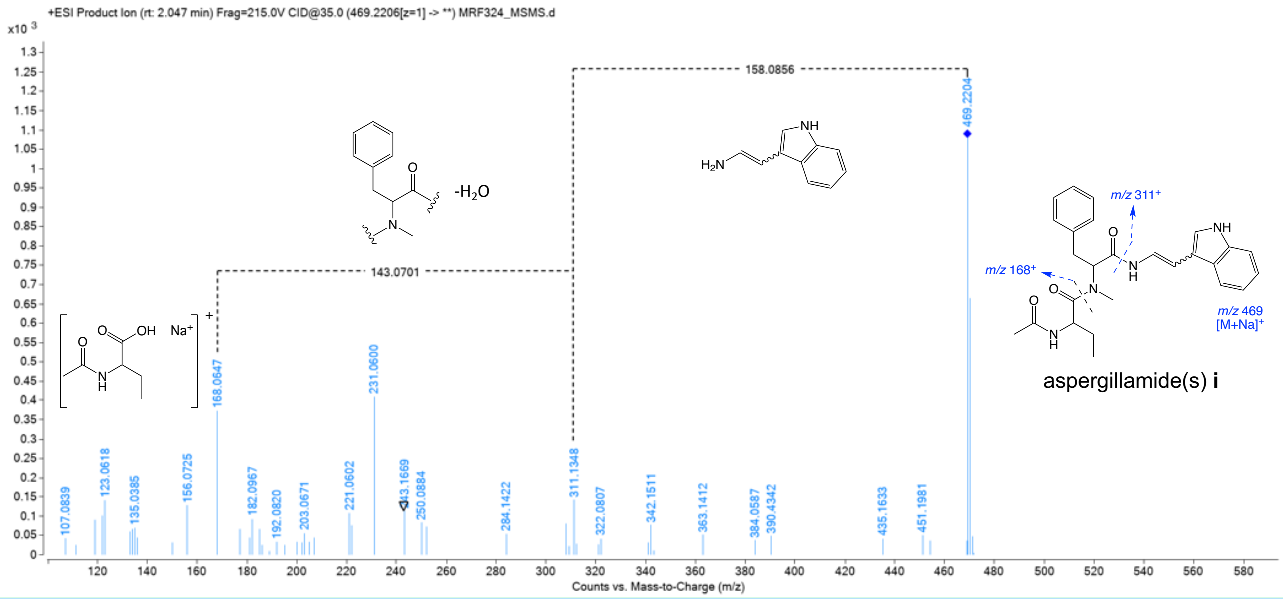

○	MFG		C26 H30 N4 O3	(M+Na)+	469.2206	98.62			-0.99			98.62	

Species	m/z	Score (iso. abund)	Score (mass)	Score (MFG, MS/MS)	Score (MS)	Score (MFG)	Score (iso. spacing)	Height	Ion Formula	Lib/DB	RT	RT Diff
(M+Na)+	469.2206	96.69	99.09		98.62	98.62	100	328584.4	C26 H30 N4 Na O3			

Height (Calc)	Height Sum% (Calc)	Height % (Calc)	m/z (Calc)	Diff (mDa)	Height	Height %	Height Sum %	m/z	Diff (ppm)
320125.8	74.07	100	469.221	-0.4	328584.4	100	76.02	469.2206	-0.94
96170.7	22.25	30.04	470.2241	-0.4	91844.3	27.95	21.25	470.2237	-0.93
15923.6	3.68	4.97	471.2269	-0.5	11791.3	3.59	2.73	471.2264	-1.07

Figure S60. MS/MS fragmentation and HRMS analysis for aspergillamide(s) **i**.

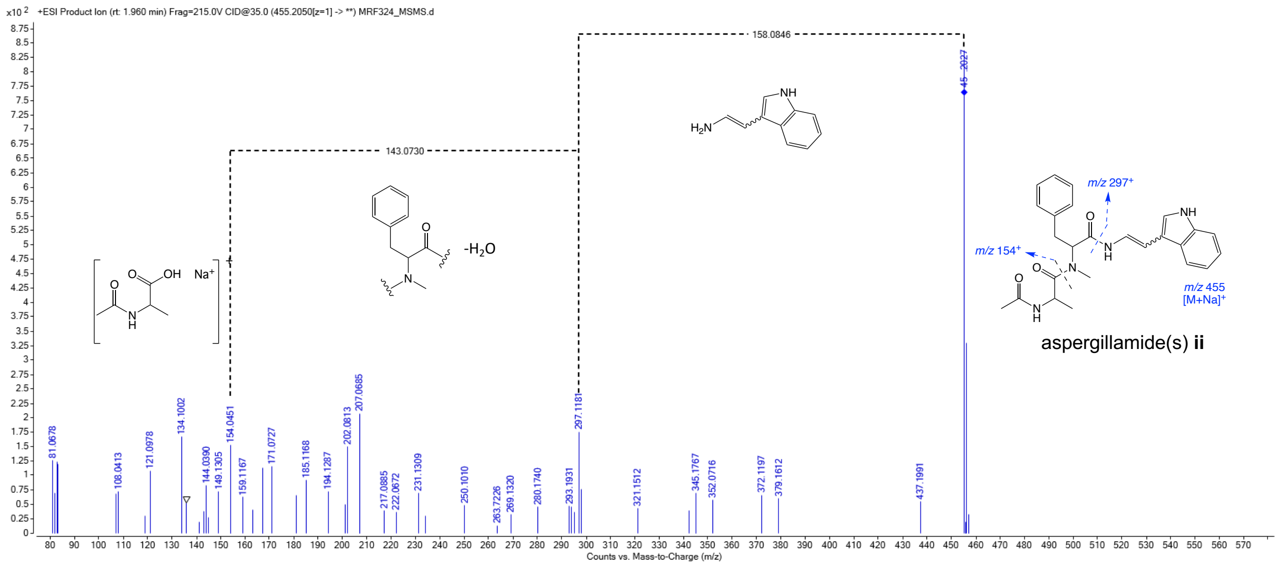

○	MFG		C25 H28 N4 O3	(M+Na)+	455.2050	95.79			-0.9			95.79	

Species	m/z	Score (iso. abund)	Score (mass)	Score (MFG, MS/MS)	Score (MS)	Score (MFG)	Score (iso. spacing)	Height	Ion Formula	Lib/DB	RT	RT Diff
(M+Na)+	455.205	87.25	99.28		95.79	95.79	99.06	130126.5	C25 H28 N4 Na O3			

Height (Calc)	Height Sum% (Calc)	Height % (Calc)	m/z (Calc)	Diff (mDa)	Height	Height %	Height Sum %	m/z	Diff (ppm)
123935.1	74.86	100	455.2054	-0.4	130126.5	100	78.59	455.205	-0.84
35863	21.66	28.94	456.2084	-0.1	31599.5	24.28	19.09	456.2083	-0.32
5768.3	3.48	4.65	457.2112	-2.6	3840.4	2.95	2.32	457.2087	-5.62

Figure S61. MS/MS fragmentation and HRMS analysis for aspergillamide(s) **ii**.

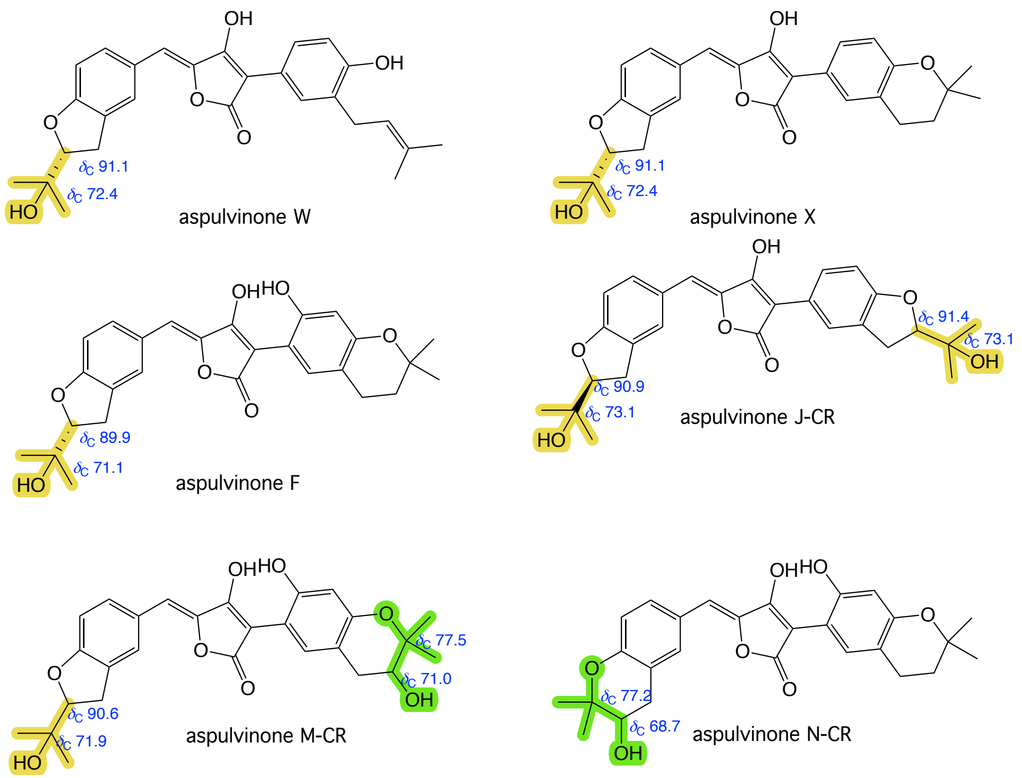

Figure S62. Chemical shifts of C-8″ and C-9″ for dihydrobenzofuran (yellow highlight) and dihydrobenzopyran (green highlight) moiety.
